# The orexin system as a pharmacological target in inflammation: peripheral mechanisms and safety implications

**DOI:** 10.3389/fphar.2026.1831913

**Published:** 2026-08-03

**Authors:** Daniel Stránský, Michal Barabas, Martin Šíma, Ondřej Slanař

**Affiliations:** 1 Department of Pharmacology, First Faculty of Medicine, Charles University and General University Hospital, Prague, Czechia; 2 Department of Anaesthesiology, Resuscitation and Intensive Medicine, First Faculty of Medicine, Charles University and General University Hospital, Prague, Czechia

**Keywords:** corticosteroids, HPA axis, hypocretin, IBD, immune system, orexin A and B, orexin receptors, pharmacokinetics

## Abstract

The orexin (hypocretin) system, composed of the neuropeptides orexin A and orexin B and their receptors OX1R and OX2R, is primarily known for its role in sleep–wake regulation and energy homeostasis. Increasing evidence indicates that orexin signaling also participates in the regulation of inflammatory processes. This review examines the pharmacological relevance of the orexin system in immune modulation, with particular emphasis on potential peripheral mechanisms that may represent novel therapeutic targets. Current evidence suggests that orexins may influence inflammatory responses through both central and peripheral pathways, including modulation of the hypothalamic–pituitary–adrenal axis, autonomic nervous system activity, regulation of adrenal corticosteroid secretion, reduction of oxidative stress, and potential direct effects on immune cells expressing orexin receptors. A major unresolved question is the origin and function of circulating orexin peptides detected in human plasma despite their predominantly central production. We also review the limited pharmacokinetic data available for orexin peptides and evaluate reported plasma concentrations, highlighting substantial methodological variability that complicates interpretation of circulating levels and their potential peripheral sources. Distinguishing between central and peripheral orexin actions is pharmacologically important. While orexin receptor antagonists are already approved for insomnia and agonists are being developed for narcolepsy, their potential effects on immune function warrant further consideration. Conversely, peripheral or immune-specific variants of orexin signaling could represent promising therapeutic targets with reduced central nervous system effects.

## 1 Introduction

Compelling experimental and preclinical evidence indicates that the orexin system participates in the regulation of inflammatory processes. Orexin A has been shown to modulate immune responses through activation of orexin receptors, exerting immunoregulatory and anti-inflammatory effects in both the nervous system and peripheral tissues.

From a pharmacological perspective, an important question is whether these effects are mediated predominantly through central mechanisms or whether peripheral tissues directly contribute to orexin-mediated regulation of inflammation. Central mechanisms may involve modulation of the hypothalamic–pituitary–adrenal (HPA) axis and autonomic nervous system activity, both of which exert profound effects on immune responses. However, pharmacological manipulation of central orexin signaling is associated with significant physiological consequences, including alterations in sleep, cognition, and metabolism, which may limit the therapeutic applicability of centrally acting orexin-based drugs for inflammatory conditions.

In contrast, peripheral orexin signaling pathways could offer opportunities for more selective pharmacological interventions. Evidence for orexin receptor expression in peripheral tissues, including immune cells, adrenal glands, and the gastrointestinal tract, raises the possibility that orexins may directly regulate inflammatory responses outside the central nervous system. If confirmed, such mechanisms could represent novel therapeutic targets with reduced central nervous system side effects.

This narrative review evaluates current knowledge on the relationship between the orexin system and inflammation from a pharmacological perspective. Particular emphasis is placed on distinguishing central and peripheral mechanisms, assessing the therapeutic potential of targeting orexin signaling in inflammatory diseases, and discussing safety considerations associated with currently available orexin-modulating drugs. The review is organized into chapters structured around the following conceptual framework:

- Receptor biology (pharmacodynamics): whether orexin receptors are expressed on immune-related targets and capable of modulating inflammatory signaling.
- Peripheral tissue distribution (extended pharmacodynamic context): whether components of the orexin system are present in immune-relevant tissues, and which of these tissues may contribute to immunoregulation.
- Pharmacokinetics: whether orexins are detectable in circulation, their origin (central vs. peripheral), their ability to cross the blood–brain barrier, their stability in blood, and their physiological concentrations.
- HPA axis involvement (PDPK interface): whether orexins influence the hypothalamic-pituitary-adrenal axis exclusively *via* central mechanisms or also through direct peripheral effects (e.g., adrenal glucocorticoid secretion), including receptor involvement and potential local production.
- Functional and translational evidence (clinical relevance): the extent of evidence supporting immunomodulatory effects of orexins across clinical studies, *in vivo* models, and *in vitro* systems.

These components are integrated in the Discussion, where mechanistic links are proposed and key research gaps are identified. Searched was done between 1 January 2025 and 27 January 2026.

In order to find articles suitable for this review we searched through database of scientific literature: PubMed; Scopus, Web of Science. We searched through these databases using combinations of the following keywords: “adrenal”, “autoimmune”, “blood”, “cells”, “colon”, “corticosteroids”, “corticosterone”, “cortisol”, “dimer”, “distribution”, “DORA”, “ENS”, “enteric nervous system”, “expression”, “gland”, “H295R”, “heart”, “heterodimer”, “HPA”, “hypocretin”, “IBD”, “immune”, “inflammation”, “intestine”, “kidney”, “level”, “liver”, “lung”, “lymphocyte”, “macrophage”, “microglia”, “multiple”, “muscle”, “narcolepsy”, “OX1R”, “OX2R”, “Orexin”, “Orexin A”, “Orexin B”, “ovaries”, “pancreas”, “pharmacokinetics”, “placenta”, “plasma”, “prostate”, “sepsis”, “septic”, “serum”, “sclerosis”, “splice”, “SORA”, “SORA1”, “SORA2”, “spleen”, “stomach”, “stress”, “testis”, “thymus”, “tissue”, “uterus”.

Subsequently, reports describing studies in non-mammalian organisms and animal cancer tissues were excluded. Preprints, conference abstracts, and clinical trial registries were not searched and were therefore not included in this review.

**FIGURE 1:**
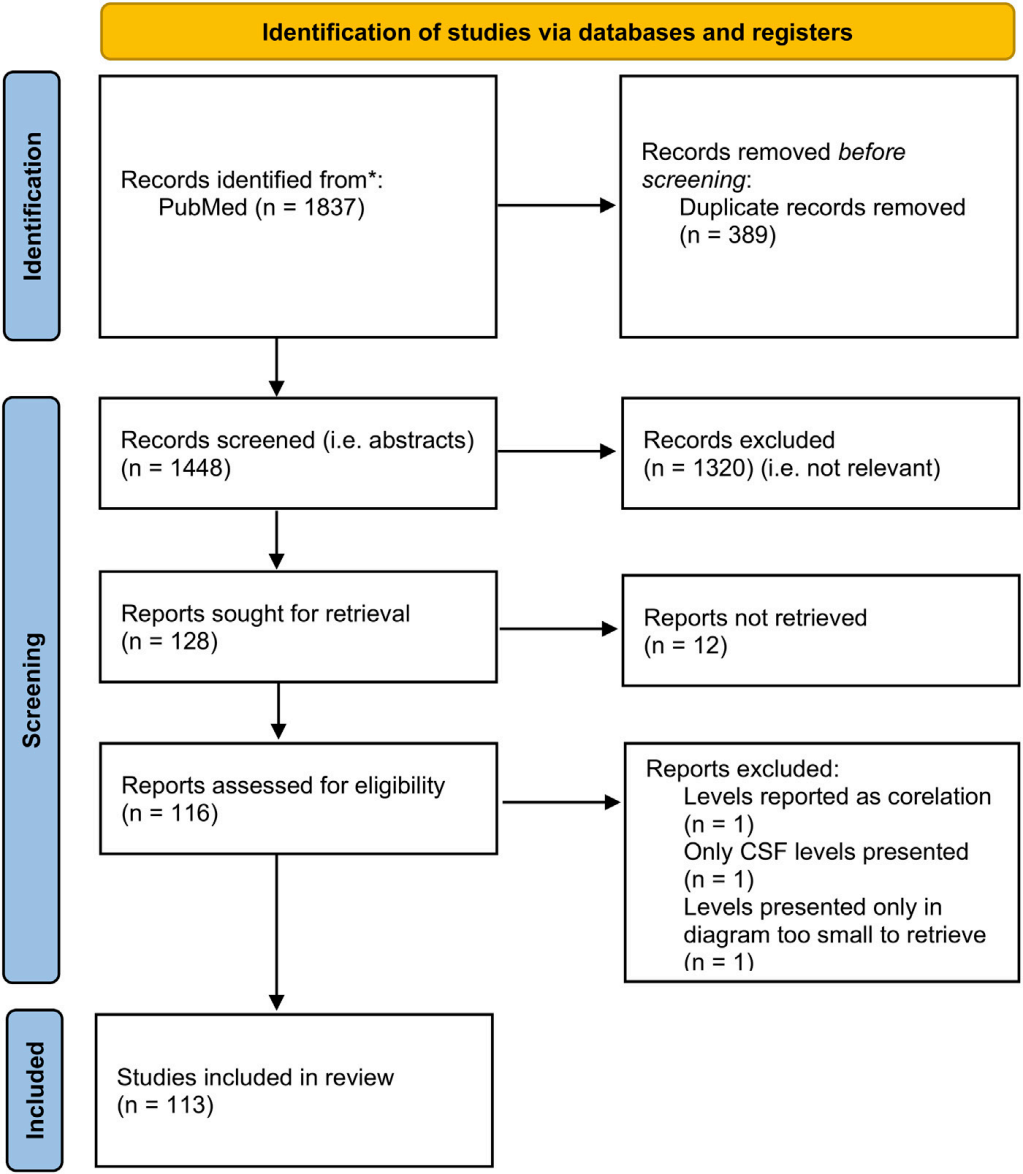
PRISMA 2020 flow diagram of study selection process for systematic review of circulating levels of orexins.

To assess circulating orexins concentrations, a systematic search of PubMed was performed using the keyword combinations “orexin plasma,” “orexin serum,” and “orexin blood.” The search term “orexin plasma” yielded 445 records, of which 66 studies reported measurements of orexin levels in humans. 11 of these studies were excluded because quantitative ranges could not be extracted (e.g., data presented only as correlations or qualitative descriptions) or because the full article could not be retrieved. The search term “orexin serum” returned 303 records; after removal of duplicates identified in the previous search, 43 studies measuring orexin levels in humans were identified, of which 2 were excluded for the same reasons. The search term “orexin blood” yielded 1,089 records and, after exclusion of duplicates from the prior searches, identified an additional 19 human studies, of which 2 were excluded for the same reasons. Summary of included and excluded studies is provided in Supplementary Table S1. Flowchart is presented in Figure 1.

## 2 Orexin system

In 1998 two independent research groups discovered a pair of previously unknown hypothalamic neuropeptides, subsequently named orexins (also termed hypocretins) (de Lecea et al., 1998; Sakurai et al., 1998).

**FIGURE 2:**
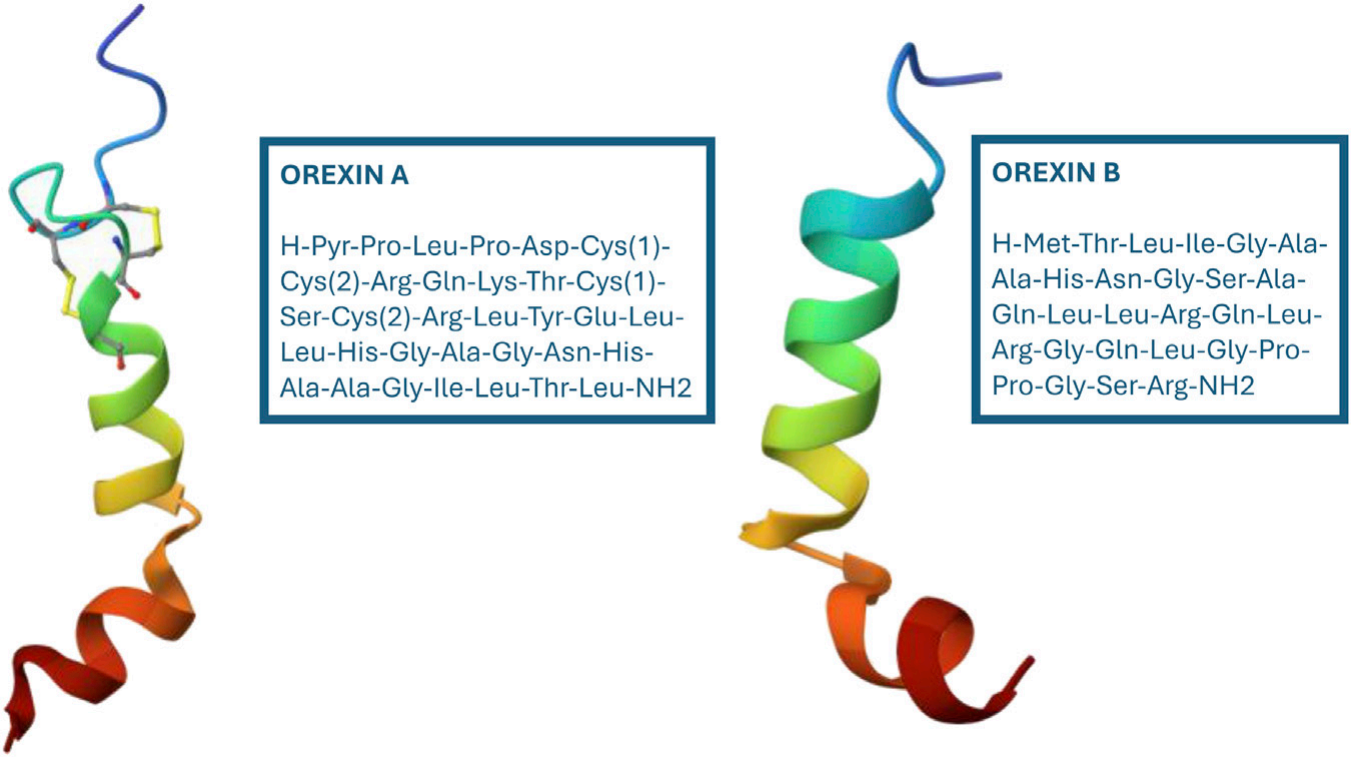
Orexin A and B peptides structures. Images obtained from the RCSB Protein Data Bank (PDB ID: 1R02; 1CQ0), originally based on coordinates published by Kim et al. (2004) and Lee et al. (1999) respectively. For orexin A two more possible structures were discovered (Takai et al., 2006).

The orexin system was initially characterized for its role in regulating food intake, followed subsequently by its function in sleep and wakefulness regulation (Inutsuka and Yamanaka, 2013). Beyond these, orexins have been implicated in diverse physiological processes including stress regulation, reward, nociception, emotional regulation, energy metabolism, and even cardiorespiratory system control (Inutsuka and Yamanaka, 2013; Lawrence, 2017). Two neuropeptides, orexin A (OxA) and orexin B (OxB), derive from a common precursor, prepro-orexin, through proteolytic cleavage (Saitoh and Sakurai, 2023). OxA is a 33-amino acid peptide containing two disulfide bonds that primarily confer peptide stability rather than directly influencing receptor activation (Saitoh and Sakurai, 2023). OxB is a 28-amino acid peptide lacking these disulfide bonds (both peptides structures are depicted in Figure 2) (Saitoh and Sakurai, 2023). Both OxA and OxB activate two G protein-coupled receptors: orexin receptor 1 (OX1R) and orexin receptor 2 (OX2R) (Sakurai et al., 1998). OxA exhibits near-equivalent affinity for OX1R and OX2R, with EC50 values of approximately 30 nM and 34 nM, respectively, whereas OxB preferentially binds OX2R (EC50 ≈ 60 nM), showing markedly reduced affinity for OX1R (EC50 ≈ 2,500 nM) (Sakurai et al., 1998). The orexin system remains evolutionarily very conserved, with the OxA amino acid sequence remaining completely identical in all mammals tested so far (e.g., human, mouse, rat) (Soya and Sakurai, 2020). Critical amino acids residues for OX1R activation are also well conserved in OxA and OxB across various species (Saitoh and Sakurai, 2023).

### 2.1 Receptors

Orexin receptors belong to the G protein-coupled receptor (GPCR) superfamily (Couvineau et al., 2021; Couvineau et al., 2022; Dale et al., 2022; Alain et al., 2021; Kukkonen et al., 2024; Kukkonen and Leonard, 2014; Kukkonen and Turunen, 2021; Wang et al., 2018; Yin et al., 2022). Although the complete mechanisms of orexin receptor signal transduction remain to be fully elucidated, their primary signaling is mediated *via* coupling to the Gq subunit, which activates phospholipase C (PLC), resulting in inositol trisphosphate-dependent intracellular Ca^2+^ mobilization and diacylglycerol generation with further downstream activation of phospholipase A2 (PLA2) (Dale et al., 2022; Kukkonen et al., 2024; Kukkonen and Leonard, 2014; Wang et al., 2018), mammalian target of rapamycin (mTOR) (Kukkonen and Turunen, 2021; Wang et al., 2018; Wang Z. et al., 2014) and protein kinase C (PKC) signaling including phosphorylation of mitogen-activated protein kinases (MAPK) such as extracellular signal-regulated kinases (ERK1/2), and p38 (Couvineau et al., 2021; Couvineau et al., 2022; Dale et al., 2022; Alain et al., 2021; Kukkonen et al., 2024; Kukkonen and Leonard, 2014; Wang et al., 2018) and activation of phospholipase D1 (Dale et al., 2022; Kukkonen et al., 2024; Kukkonen and Leonard, 2014; Kukkonen and Turunen, 2021; Wang et al., 2018). Secondary involvement of Gi and Gs subunits is thought to modulate adenylate cyclase activity, influencing cyclic AMP levels and downstream effectors including protein kinase A (PKA) (Dale et al., 2022; Alain et al., 2021; Kukkonen et al., 2024; Kukkonen and Leonard, 2014; Wang et al., 2018). However, the involvement of Gi and Gs signaling is considerably less well defined, particularly with respect to tissue- and cell type–specific effects and differences between OX1R and OX2R (Kukkonen et al., 2024; Gratio et al., 2025).

Phosphoinositide 3-kinase (PI3K)/protein kinase B (Akt) signaling was also confirmed to contribute to orexin downstream effects but exact mechanism how it is activated remains unsolved (Kukkonen et al., 2024; Kukkonen and Leonard, 2014; Kukkonen and Turunen, 2021). One possibility could be the activation by Gβγ subunit, which is an activator of PIK3/Akt (Chen et al., 2024). A recent study demonstrated that both OX1R agonists and antagonists can activate Gβγ signaling, leading to apoptosis in tumor cells (Gratio et al., 2025). Although the PI3K/Akt pathway is generally associated with anti-apoptotic signaling, under certain conditions it may also contribute to apoptotic processes (Guo et al., 2009; Mostefai et al., 2008).

Additionally, β-arrestin mediated signaling, c-Jun N-terminal kinase (JNK) pathways, and protein tyrosine phosphatase SHP2 contribute to apoptosis regulation and cellular responses (Couvineau et al., 2022; Alain et al., 2021; Kukkonen et al., 2024; Kukkonen and Leonard, 2014; Kukkonen and Turunen, 2021).

In neuronal systems, orexin receptor activation triggers extracellular Ca^2+^ influx predominantly *via* non-selective cation channels (NSCC), sodium-calcium exchangers (NCXs) and transient receptor potential (TRPs) channels (Kukkonen et al., 2024; Kukkonen and Leonard, 2014; Wang et al., 2018). Together with Inward-rectifier potassium channels (KIRs) and TWIK-related acid-sensitive K+ channels (TASKs) regulating inward and outward potassium leak contributes orexin signaling to modulation of neuronal excitability and synaptic transmission (Kukkonen et al., 2024; Kukkonen and Leonard, 2014).

The diverse formation of GPCR heterodimers and the signal bias of orexin receptors contribute to the complexity of signal transduction, resulting in context-dependent cellular responses (Kukkonen et al., 2024; Kukkonen and Turunen, 2021; Hilairet et al., 2003; Chen et al., 2015).

**FIGURE 3:**
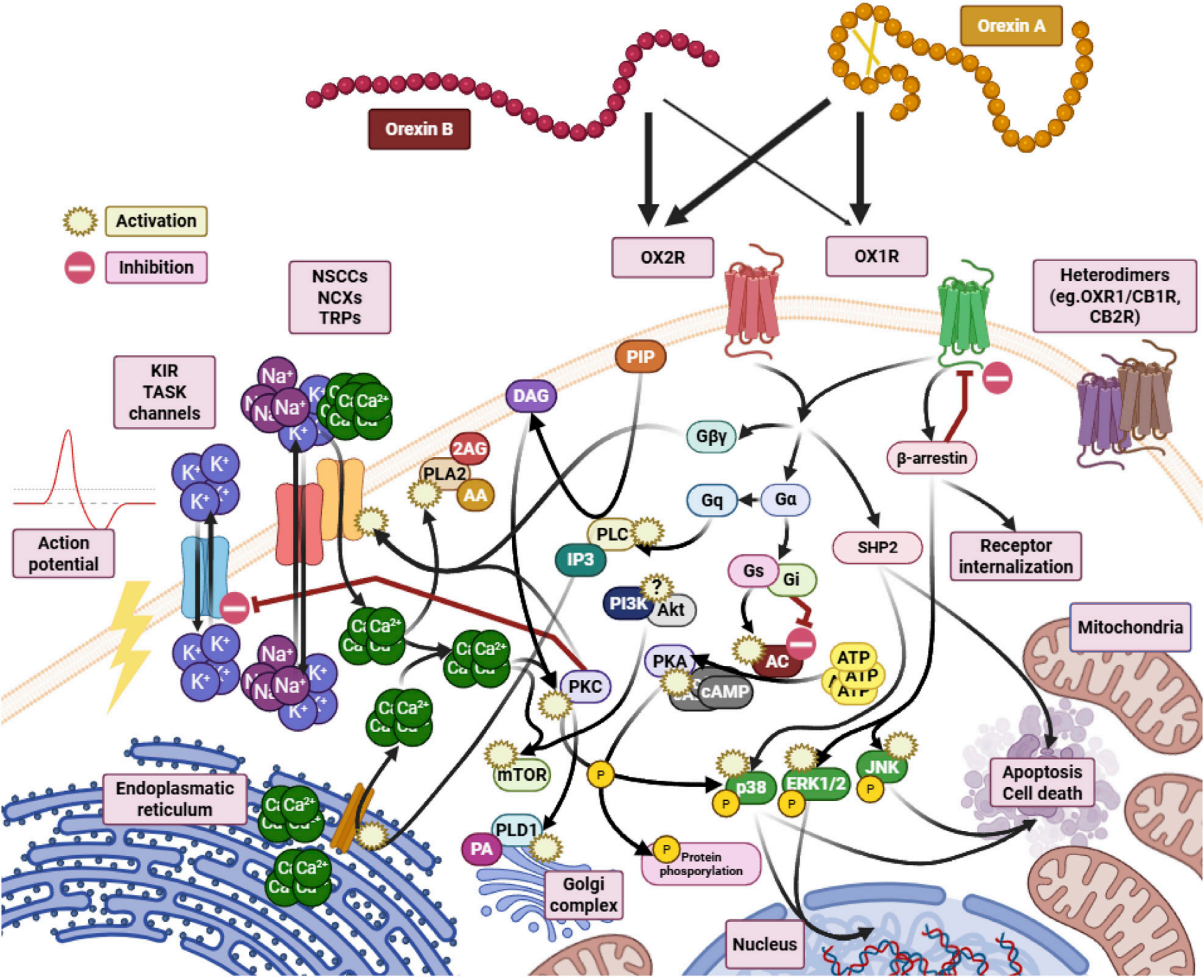
Simplified scheme of possible orexin downstream signalling. 2AG, 2-Arachidonoylglycerol; AA, Arachidonic acid; AC, Adenylyl cyclase; Akt, Protein kinase B; ATP, Adenosine triphosphate; cAMP, Cyclic adenosine monophosphate; CB1R, Cannabinoid receptor 1; DAG, Diacylglycerol; ERK, Extracellular signal-regulated kinase; Gα (Gq, Gs, Gi), Gβγ, G protein-coupled receptor subunits; IP3, Inositol trisphosphate; JNK, c-Jun N-terminal kinase; KIR, Inward-rectifier potassium channel; mTOR, Mammalian target of rapamycin; NSCC, Non-selective cation channel; NCX, Sodium-calcium exchanger; OX1R, Orexin receptor type 1; OX2R, Orexin receptor type 2; PA, Phosphatidic acid; PIP, Phosphoinositide; PI3K, Phosphoinositide 3-kinase; PKA, Protein kinase A; PKC, Protein kinase C; PLA2, Phospholipase A2; PLC, Phospholipase C; PLD1, Phospholipase D1; SHP2, Src homology region two domain-containing phosphatase-2; TASK, TWIK-related acid-sensitive K+ channel; TRP, Transient receptor potential channel. Created in BioRender.com.

Simplified scheme of orexin downstream signaling is depicted in Figure 3.

Functionally, OX2R predominates in regulating circadian rhythms and sleep–wake homeostasis, whereas OX1R, a phylogenetically younger receptor subtype, primarily mediates reward processing, nociception, and stress-related pathways (Lawrence, 2017).

Several studies have suggested a direct action of OxA on immune cells or anti-inflammatory effects in various non-immune cell types (see chapters 5.2.1 and 5.2.2); however, the underlying intracellular signaling mechanisms have not yet been clearly defined. To date, the only signaling cascade that has been directly investigated and described in an inflammatory or immune-related context involves Gq-dependent activation of PLC, subsequent intracellular Ca^2+^ mobilization, downstream modulation of MAPK pathways (p38, JNK, ERK), inhibition of NF-κB signaling, and consequent suppression of pro-inflammatory mediators, including TNF, IL-1β, IL-6, IL-8, and inducible nitric oxide synthase (iNOS) (Chen et al., 2024; Guo et al., 2009; Mostefai et al., 2008). This pathway has been directly implicated in the anti-inflammatory effects of OxA in HT-29 colorectal adenocarcinoma cells, U251 astroglioma cells, and microglial cells (Messal et al., 2018; Guo et al., 2024b; Xu et al., 2021).

As previously mentioned, a recent study reported that both agonists and antagonists can activate Gβγ signaling, which in turn regulates the apoptosis in tumor cells (Gratio et al., 2025). In most animal models, however, orexin receptor antagonists abolish the anti-inflammatory effects of OxA, suggesting that Gβγ is unlikely to be the primary transducer of this anti-inflammatory response (Messal et al., 2018; Guo et al., 2024b; Fatemi et al., 2016; Li et al., 2020).

Interestingly, a recent study has reported anti-inflammatory effects of daridorexant in the brain and lungs following systemic LPS administration in mice (Horiuchi et al., 2026). Since SORAs are typically used to demonstrate blockade of OxA–mediated anti-inflammatory effects, it is possible that DORAs, particularly at high doses (e.g., 108 mg/kg in this study), may induce signaling bias and partially mimic orexin-like effects (Gratio et al., 2025). In addition, given that daridorexant is primarily targeting CNS, a centrally mediated anti-inflammatory mechanism cannot be excluded as an alternative explanation.

In addition to this signaling cascade, several other pathways could theoretically contribute to the direct effects of orexins on immune cells or anti-inflammatory effects in various non-immune cell types, including activation of PLA2 and subsequent production of lipid mediators such as arachidonic acid (Turunen et al., 2010), engagement of the PI3K/Akt - mTOR pathway (Liu et al., 2016; Wan et al., 2017), β-arrestin–mediated signaling (Dale et al., 2022; Dale et al., 2026; Jaeger et al., 2014) and modulation of transient receptor potential (TRP) channels (Nasman et al., 2006; Larsson et al., 2005). Although all of these pathways play important roles in immune cell signaling (Murakami et al., 2016; Xie et al., 2014; Parenti et al., 2016; Shi et al., 2007) and have been described as components of orexin receptor signal transduction, we were unable to identify studies providing direct evidence for their involvement in the direct effects of orexins on immune cells or in the mediation of anti-inflammatory effects in non-immune cell types.

Lastly, we can mention formation of GPCR heterodimers with cannabinoid receptor 1 (CB1R) (Hilairet et al., 2003; Ward et al., 2011; Ellis et al., 2006) and cannabinoid receptor 2 CB2R (Raich et al., 2022). Although CB2R is the predominant mediator of endocannabinoid signaling in the immune system with known anti-inflammatory effect (Turcotte et al., 2016), emerging evidence suggests that CB1R also contributes to immune modulation (Rahaman and Ganguly, 2021).

### 2.2 Therapeutic strategies to modulate orexin signalling

Therapeutic translation of orexin biology has proceeded through two opposite pharmacological strategies: receptor activation and receptor blockade. However, the therapeutic clinical utilization of the orexin system modulation has been very limited so far.

The first group of approved drugs are dual orexin receptor antagonists (DORAs), which are used to treat insomnia, a condition that affects approximately 5%–20% of the European population (Riemann et al., 2017; Bragg et al., 2019; Muehlan et al., 2020; Waters, 2022). By attenuating orexin-driven excitatory input to wake-promoting monoaminergic and cholinergic nuclei, DORAs restore physiological sleep architecture without the global GABAergic suppression characteristic of benzodiazepines and z-hypnotics. This receptor-targeted mechanism accounts for their more favorable safety profile, especially the reduced risk of cognitive impairment, falls, parasomnias, and next-day sedation, consistent with the absence of widespread CNS depression (Muehlan et al., 2020). There were several DORAs which did not make it to the market whether due to lack of efficacy, poor pharmacokinetic profile, adverse effects, or were just quietly discontinued. Notable examples include almorexant, which was discontinued following safety concerns, particularly elevations in liver enzymes (Hoch et al., 2014; Jacobson et al., 2022) and filorexant (Jacobson et al., 2022) or JNJ-48816274 (Kim and Kim, 2024). From the molecules which did reached the market, suvorexant, the first-in-class DORA approved in Japan in 2014, demonstrated dose-dependent suppression of wake-promoting orexin signaling at doses of 5–20 mg nightly (Muehlan et al., 2020; Merck, 2025). Lemborexant, approved in the United States in 2019, displayed a longer half-life with reportedly improved sleep-maintenance efficacy at doses of 5–10 mg (Muehlan et al., 2020; Ardeljan and Hurezeanu, 2021). More recently, daridorexant became the first DORA approved in Europe (2022), with optimized pharmacokinetic/pharmacodynemic relationship to minimize next-day somnolence at 25–50 mg nightly (Muehlan et al., 2020; EMA, 2025). The most recently agent on the market is vornorexant, approved in Japan in 2025, characterized by rapid absorption and the shortest half-life among currently available DORAs, administered at nightly doses of 2.5–10 mg (Chino et al., 2025; Uchiyama et al., 2026). DORAs may represent a suitable therapeutic approach for insomnia in patients with neurodegenerative disorders, including Alzheimer’s disease (Carpi et al., 2024). Given the growing interest in the involvement of the orexin system in the pathogenesis of neurodegenerative diseases (Zhou et al., 2020; Lucey et al., 2018; Holth et al., 2019; Gabelle et al., 2019; Hada et al., 2026; Parhizkar et al., 2025), an early study investigating the impact of DORAs on the onset and progression of Alzheimer’s disease has also been published (Lucey et al., 2023).

Selective OX2R antagonists are under active investigation mainly as treatments for insomnia and psychiatric conditions with sleep disturbances such as major depressive disorder with insomnia symptoms (Jha, 2022). Experimental data also indicate OX2R antagonists may alleviate aggression and anxiety by modulating hypothalamic and habenular circuits implicated in these behaviors (Jha, 2022). For example, seltorexant is a selective OX2R antagonist showing efficacy in improving sleep and depressive symptoms in phase III trials (Jha, 2022; Mesens et al., 2025a). OX2R antagonism reduces orexin-driven wake-promoting signalling pathways, facilitating sleep initiation and maintenance with a potentially milder side effect profile than dual antagonists (Jha, 2022).

Mechanistically, selective OX1R antagonism modulates neural circuits involved in reward, anxiety, compulsive behaviors, and stress responses without markedly promoting sleep (Hopf, 2020). This receptor-specific action may offer therapeutic benefits for neuropsychiatric and addictive disorders without inducing sleepiness or impairing wakefulness (Hopf, 2020). Selective OX1R antagonists have therefore emerged as a candidate class of compounds (Williams et al., 2024). An example is nivasorexant (ACT-539313), the first selective OX1R antagonist to enter clinical trials and shown to reduce binge-eating behavior in phase II studies, with potential applications in substance use disorder, anxiety, and obsessive-compulsive disorders. Other selective OX1R antagonists, such as JNJ-61393215, are in early trials targeting mood and anxiety disorders (Salvadore et al., 2020).

Selective OX2R agonists represent a promising approach for the treatment of narcolepsy type 1, a condition caused by the selective loss of orexin-producing neurons in the lateral hypothalamus (Barateau and Dauvilliers, 2019; Thorpy, 2020). Among these, TAK-925 (danavorexton), a subcutaneously administered non-peptide OX2R agonist, demonstrated promising signs of efficacy in early-phase clinical studies but its development was discontinued due to slow patient recruitment (Weber et al., 2024). A related compound, TAK-994 (firazorexton), an orally available selective OX2R agonist, progressed to phase II trials but was terminated due to safety concerns, specifically drug-induced liver injury (Dauvilliers et al., 2023). More recently, TAK-861 (oveporexton), another oral OX2R agonist, successfully completed phase II clinical trials and advanced to phase III in 2025 (Dauvilliers et al., 2025). Additional OX2R agonists are in earlier stages of development, including ALKS 2680 and ORX750 (phase II) and E2086 (phase I) (Arnett, 2025).

With respect to the mechanistic effects of OX1R stimulation, there are currently no selective OX1R agonists in advanced drug development stages.

### 2.3 Peripheral tissue distribution in humans

Orexin system distribution in the central nervous system (CNS), and in the adrenal gland in humans is well documented (see chapter 3) (Saitoh and Sakurai, 2023; Mazzocchi et al., 2001a; Guo X. et al., 2024; Randeva et al., 2001; Blanco et al., 2002; Frick et al., 2025). However, in humans, beyond the aforementioned compartments the evidence for presence of orexin system is more scarce.

A key question is whether orexin-mediated regulation of inflammation is governed primarily by central mechanisms within the central nervous system (CNS) or whether peripheral tissues also contribute to this process. In particular, it remains unclear whether immune cells themselves participate directly in orexin signalling and whether local paracrine mechanisms may be involved. Interestingly one study with robust methodology found no difference in OxA levels between narcolepsy patients and healthy controls in blood (HM et al., 2021). Very low levels of OxA were detected only in cerebrospinal fluid (CSF) of narcolepsy patients, healthy controls had normal levels. This discrepancy suggests the existence of peripheral sources of OxA independent of central orexinergic neurons. Consequently, several important questions remain unresolved: what is the physiological purpose of peripheral orexin, what tissues produce it, which targets it acts upon, and how its production is regulated. Addressing these questions may also provide important insights into a potential role of the orexin system in the regulation of immune function.

Nakabayashi et al. (2003) reported peripheral OxA distribution in the human gastrointestinal system (namely, tissue of stomach, duodenum, jejunum, ileum, colon, rectum, pancreas) and also in the placenta, but no peripheral OxA presence was detected in other organs (such as lung, kidney, heart, liver, spleen …). The same study reported prepro-orexin mRNA expression in the kidney, placenta, stomach, and ileum, with weaker signals detected in the adrenal gland, pancreas, and colon, while no expression was observed in the liver, spleen, thymus, bronchus, lung, heart, bone marrow, esophagus, duodenum, or aorta (Nakabayashi et al., 2003). In another study OxA was also present in nerve fibres and endocrine cells in stomach, small intestine and pancreas (islet of Langerhans) (Ehrstrom et al., 2005a). Increased OX1R expression was found in the mucosa of patients with ulcerative colitis (Messal et al., 2018). Contrary to previous reports of OxA in gastrointestinal tract (GIT) enteric nervous system one study claims to find no evidence for OxA producing cells in enteric nervous tissue using previously used antibodies (Baumann et al., 2008). OX1R and OX2R expression was reported in human epididymis (Leydig cells, myoid cells of the seminiferous tubules, and Sertoli cells), penis, and seminal vesicle, while expression of prepro-orexin was detected in epididymis and penis (Karteris et al., 2004). Correspondingly, OxA distribution was found in testis in another study (Ozkanli et al., 2018). In another study kidney tissues expressed OxA, OX1R, and OX2R at both peptide and transcript levels in contrast to study by Nakabayashi et al. (2003) where no presence of OxA but only presence of prepro-orexin was found (Takahashi et al., 2006). Both orexin receptors were also found in the human heart (Patel et al., 2018). Prepro-orexin expression was also detected in placenta, but same study did not find any expression of prepro-orexin, OX1R or OX2R in prostate or prostate cancer tissue (Szyszka et al., 2015). Contrary to this finding, another study found expression of OX2R but not expression of prepro-orexin and OX1R in prostate and benign prostatic hyperplasia samples (Malendowicz et al., 2011). Furthermore, OX2R, but not OX1R, was demonstrated in placental tissue (Taximaimaiti et al., 2016) and a study detected OX2R in endometrium (Dehan et al., 2013). In contrast, another study has failed to detect OX1R or OX2R expression in female reproductive tract tissues such as placenta and myometrium (Randeva et al., 2001). Summary of these individual studies is provided in Table 1.

**TABLE 1:** Comparison of studies investigating presence of OX1R, OX2R, prepro-orexin, OxA or OxB in selected human tissues.

Publication	Methodology	Target
			Pituitary	GI tract			Reproductive system					Bone marrow and lymphoid tissue			
				Stomach	Intestines	Pancreas	Uterus	Placenta	Ovaries	Prostate	Testis	Bone marrow	Spleen	Tonsils	Thymus
Arihara et al., 2000	IH	OxA	X												
Blanco et al., 2003	IH, IF		O^a^												
Nakabayashi et al., 2003	IH			O^b^	O^b^	O^c^	X	O^d^	X	X	X		X	X	
Karteris et al., 2004	WB										X				
Ehrstrom et al., 2005a	IH			O^b^	O^b^	O^e^									
Baumann et al., 2008	IH			X	X										
Ozkanli et al., 2018	IH										O^f^				
Blanco et al., 2003	IH, IF	OxB	O^g^												
Karteris et al., 2004	WB										X				
Nakabayashi et al., 2003	PCR	prepro-orexin		O	O^h^	O		O				X			X
Karteris et al., 2004	PCR										X				
Malendowicz et al., 2011	PCR									X					
Szyszka et al., 2015	PCR							O		X^i^					
Blanco et al., 2001	IH, PCR	OX1R	O^j^												
Randeva et al., 2001	PCR						X^k^	X							
Karteris et al., 2004	PCR, WB, IF										O^l^				
Ehrstrom et al., 2005a	IH			O^m^	O^m^										
Malendowicz et al., 2011	WB, PCR									X					
Szyszka et al., 2015	PCR	​								X^i^					
Taximaimaiti et al., 2016	IH							X							
Messal et al., 2018	IH				O^n^										
Blanco et al., 2001	IH, PCR	OX2R	O^g^												
Randeva et al., 2001	PCR						X^k^	X							
Karteris et al., 2004	PCR, WB, IF										O^l^				
Ehrstrom et al., 2005a	IH				O^o^										
Malendowicz et al., 2011	WB, PCR									O					
Dehan et al., 2013	IH, PCR						O^p^								
Szyszka et al., 2015	PCR									X^i^					
Taximaimaiti et al., 2016	IH							O^q^							

GI Tract, Gastrointestinal Tract; IH, Immunohistochemistry; PCR, Polymerase chain reaction; WB, Western blot; OxA, orexin A; OxB, orexin B; OX1R, Orexin receptor type 1; OX2R, Orexin receptor type 2.

^a^ Prolactin (82 ± 5.3%), Thyroid-Stimulating Hormone (18 ± 2.3%), Growth Hormone (10 ± 2.3%), Follicle-Stimulating Hormone (8 ± 2.6%), and Luteinizing Hormone (7 ± 3.2%) positive cells.

^b^ Enteric nervous system and endocrine cells.

^c^ Islet cells - 65.8% of insulin, 22.1% of somatostatin and 9.9% of glucagon positive cells.

^d^ Syncytiotrophoblasts, Decidual cells.

^e^ Islet cells - OxA immunoreactivity detected in insulin and pancreatic polypeptid positive cells but not in somatostatin or glucagon positive cells.

^f^ Leydig cells, weaker in the germ cells in seminiferous tubulus.

^g^ Adrenocorticotropic Hormone positive cells.

^h^ Negative in duodenum.

^i^ Human prostatic cell lines.

^j^ Growth Hormone positive cells.

^k^ Myometrium.

^l^ Leydig cells, testicular peritubular myoid cells, and some Sertoli cells.

^m^ Enteric nervous system cells.

^n^ Epithelium cells (only ulcerative colitis patients).

^o^ Endocrine ells.

^p^ Endometrium.

^q^ Chorionic villi, cytotrophoblast, chorion frondosum, connective tissues

We can compare results from these individual studies to large database such as The Human Protein Atlas (THPA) which integrates data from multiple datasets (Uhlen et al., 2015; Karlsson et al., 2021; Uhlen et al., 2019; Alvez et al., 2025; The Human Protein Atlasa; The Human Protein Atlasb; The Human Protein Atlasc). This summary is provided in Table 2.

**TABLE 2:** Comparison of The Human Protein Atlas (THPA) data regarding presence of OX1R, OX2R or prepro-orexin in selected human tissues.

​		THPA data tissue proteom (Uhlen et al., 2015; The Human Protein Atlasa)	THPA data tissue trasncriptom (Uhlen et al., 2015; The Human Protein Atlasa)	THPA data single cell trancriptom (Karlsson et al., 2021; The Human Protein Atlasa)	THPA data tissue proteom (Uhlen et al., 2015; The Human Protein Atlas ^c^)	THPA data tissue trasncriptom (Uhlen et al., 2015; The Human Protein Atlasc)	THPA data single cell trancriptom (Karlsson et al., 2021; The Human Protein Atlasc)	THPA data tissue proteom (Uhlen et al., 2015; The Human Protein Atlasb)	THPA data tissue trasncriptom (Uhlen et al., 2015; The Human Protein Atlasb)	THPA data single cell trancriptom (Karlsson et al., 2021; The Human Protein Atlasb)
Methodology		IH	RNAseq	RNAseq	IH	RNAseq	RNAseq	IH	RNAseq	RNAseq
Target		Prepro-orexin			OX1R			OX2R		
Pituitary		X	X	O^a^	X	O	O^a^ ^,^ ^b^	X	X	O^a^
GI Tract	Stomach	X	X	O^a^ ^,^ ^b^ ^,^ ^c^	O	X	O^c^ ^,^ ^d^	X	X	O^b^ ^,^ ^c^ ^,^ ^e^
	Small Intestine	X	X	O^a^ ^,^ ^f^ ^,^ ^g^ ^,^ ^h^	O	O	O^g^	X	X	X
	Colon	X	X	O^a^	O	X	X	X	X	O^h^
	Rectum	X	X	O^a^	O	X	O^a^	X	X	X
	Liver	X	X	X	X	X	O^b^	X	X	O^i^
	Pancreas	X	X	O^j^	O	X	X	X	X	X
Reproductive System	Uterus	X	X	X	X	O	O^k^	X	X	X
	Placenta	X	X	X	X	O	O^b^ ^,^ ^l^	X	O	O^m^
	Ovaries	X	X	O^d^	X	X	X	X	O	X
	Prostate	X	X	X	X	X	O^b^	X	X	O^d^
	Testis	X	X	O^n^ ^,^ ^o^	O	O	O^b^ ^,^ ^o^	X	O	O^o^ ^,^ ^p^ ^,^ ^q^
Heart		X	X	X	X	O	O^b^ ^,^ ^d^ ^,^ ^r^	X	O	O^s^
Kidney		X	X	O^t^	O	O	O^u^	X	O	O^t^ ^,^ ^i^
Bone Marrow and Lymphoid Tissue	Bone Marrow	X	O	O	O	O	O	X	X	X
	Spleen	X	X	O	X	O	O	X	X	X
​	Lymph	X	X	X	X	O	O	X	X	X
	Nodes	X	X	X	X	O	O	X	X	X
	Tonsil	X	X	O	O	X	O	X	X	X
	Thymus	X	X	O	X	O	O	X	X	O

Expression level:	Low	Medium	High

For the pituitary, gastrointestinal tract, reproductive system, kidney, and heart, tissue-specific cell types included in the single-cell analysis are detailed in the corresponding footnotes; immune cell‐positive populations were excluded. Conversely, for bone marrow and lymphoid tissues, only immune cell populations were considered. THPA data on immune cells are further discussed in the main text. THPA data on tissue proteome and transcriptome are derived from Uhlen et al. (2015); THPA data on single-cell transcriptome are derived from Karlsson et al. (2021); data on prepro-orexin, OX1R, and OX2R are derived from their respective entries in the THPA – The Human Protein Atlasa; The Human Protein Atlasb; The Human Protein Atlasc.

GI Tract, Gastrointestinal Tract; IH, Immunohistochemistry; PCR, Polymerase chain reaction; ppOx, prepro-orexin; WB, Western blot; OxA, orexin A; OxB, orexin B; OX1R, Orexin receptor type 1; OX2R, Orexin receptor type 2.

^a^ Endocrine cells.

^b^ Endothelial cells.

^c^ Glandular cells

^d^ Mural cells.

^e^ Finroblasts.

^f^ Secretory support cells.

^g^ Enterocytes.

^h^ Goblet cells.

^i^ Ductal cells.

^j^ Islet cells.

^k^ Secretory cells.

^l^ Endocrine cells.

^m^ Reproductive supporting cells.

^n^ Leydig cells.

^o^ Barrier epithelial cells.

^p^ Stromal cells.

^q^ Barrier epithelial cells.

^r^ Endothelial, Mural cells, Adipocytes, Fibroblasts.

^s^ Nephron cells.

^t^ Barrier epithelial cells

A strong case can be made for the presence of OX1R in the GIT, as this is supported by individual studies (Messal et al., 2018; Ehrstrom et al., 2005a) and THPA proteomics and transcriptomics data confirmed the presence there as well as further clinical and preclinical data (see chapter 5). ENS and endocrine cells may also represent sources of peripheral orexins, particularly OxA. While the latter possibility is partially supported by THPA single-cell transcriptomic data, the corresponding tissue-level proteomic and transcriptomic data do not agree with this conclusion. The ENS is not represented in the THPA database; however, evidence from individual studies suggests a potential role for the ENS as a source of peripheral OxA (Nakabayashi et al., 2003; Ehrstrom et al., 2005a). Although these observations are contradicted by the previously mentioned findings of Baumann et al. (2008) (Baumann et al., 2008). Since ENS and GIT endocrine system role in inflammation is well described some contribution of orexin system through these mechanisms could be well plausible and it is further discussed in chapter 5 (Wang et al., 2022; Khan and Ghia, 2010; Populin et al., 2021).

Presence of orexin system in reproductive system seems to be also well supported especially in placenta and testis (Nakabayashi et al., 2003; Karteris et al., 2004; Ozkanli et al., 2018; Szyszka et al., 2015; Taximaimaiti et al., 2016; The Human Protein Atlasc). However, there is no evidence supporting a role in immune regulation; instead, the available data suggest that its primary functions are related to the regulation of hormone synthesis and spermatogenesis (Ozkanli et al., 2018; Costagliola et al., 2024; Yadav et al., 2023; Dobrzyn et al., 2018). Nevertheless, since OxA has been detected in reproductive tissues in some studies (Nakabayashi et al., 2003; Ozkanli et al., 2018), these tissues may represent a potential source of peripheral OxA. Presence of orexin system (mainly the receptors) is also supported in kidneys and heart by both individual studies (Nakabayashi et al., 2003; Takahashi et al., 2006; Patel et al., 2018) and THPA data (The Human Protein Atlasb; The Human Protein Atlasc). No study detected OxA nor the prepro-orexin in the heart thus it can be ruled out as a source of peripheral OxA (Nakabayashi et al., 2003). Since two studies have confirmed the presence of OxA or prepro-orexin in the kidney (Nakabayashi et al., 2003; Takahashi et al., 2006), it may be considered another potential source of peripheral OxA. There is currently no evidence for a direct role of the kidney in orexin-mediated regulation of inflammation; however, given the established involvement of the kidney in immune and inflammatory regulation, such a relationship cannot be excluded and warrants further consideration (Neumann and Tiegs, 2021).

**FIGURE 4:**
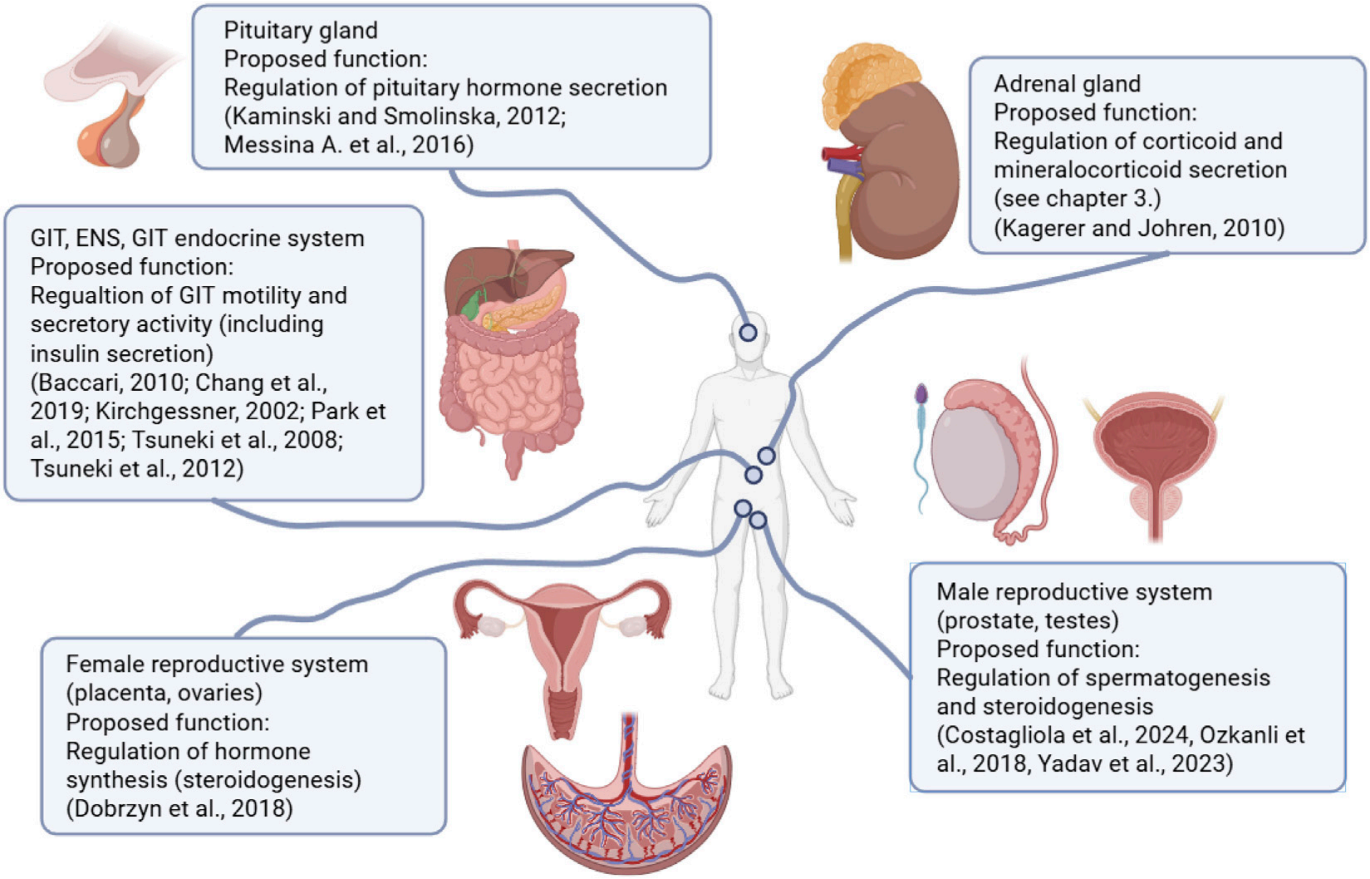
Proposed functional roles of orexin system in peripheral tissues based on data from human and animal experiments. Created in BioRender.com.

Still, orexin system functional significance in many of these tissues remains to be fully characterized. Figure 4 summarizes the human tissues and systems with the strongest evidence for the presence of the orexin system and its proposed roles, based on current data from both human and animal studies.

Regarding the presence of orexin system in immune tissues, only one human study by Nakabayashi et al. (2003) reports no presence of OxA or prepro-orexin in bone marrow, tonsils, spleen or thymus (Table 1). However, presence of the receptors, thus hormonal regulation from other sources, cannot be ruled out. In fact, Becquet et al. (2019) reported the presence of both OX1R and OX2R in murine immune organs, including the thymus, spleen, and cervical and axillary lymph nodes, with receptor expression also detected on CD4^+^ and CD8^+^ T lymphocytes as well as CD11^+^ myeloid cells. Presence of the OX1R or OX2R was also reported in some studies on human and murine immune cells (Messal et al., 2018; Sun et al., 2018). THPA data provides information from tissue proteomics and transcriptomics (Uhlen et al., 2015), single cell transcriptomics (Karlsson et al., 2021) and blood cell transcriptomics (Uhlen et al., 2019). All in combine, these data are not supportive of neither prepro-orexin or OX2R presence on immune cells (Table 2) (The Human Protein Atlasa; The Human Protein Atlasb). However, presence of OX1R on immune cells seems to be much more plausible as it was confirmed with all three methodologies although some inconsistencies are still present (The Human Protein Atlasc). For further discussion regarding functional significance see chapter 5.

Emerging evidence highlights the expression and functional significance of the orexin system in various peripheral cancers beyond the central nervous system. Contrary to discrepancies in peripheral orexin distribution, multiple studies have reported the presence of OxA, prepro-orexin, and orexin receptors, particularly OX1R, within prostate cancer tissues (Valiante et al., 2015; Basar et al., 2013). Expression of OX1R, prepro-orexin, and OxA has been confirmed in pancreatic adenocarcinoma cell lines and tumor samples, implicating the orexin system in pancreatic oncogenesis (Dayot et al., 2018; Suo et al., 2018; Voisin et al., 2022). Similarly, prostate cancer cell lines display endogenous OX1R and prepro-orexin expression (Valiante et al., 2015; Alexandre et al., 2014). OX2R expression has been documented in pheochromocytomas (Blanco et al., 2002; Mazzocchi et al., 2001b) and cervical cancer tissues (Taximaimaiti et al., 2016). Colorectal adenocarcinoma tumors and cell lines express OX1R robustly, whereas healthy colon tissues and samples from irritable bowel syndrome patients show minimal receptor presence (Gratio et al., 2025; Voisin et al., 2011; Rouet-Benzineb et al., 2004). Both OX1R and OX2R, alongside prepro-orexin, have been detected in gastric cancer and adjacent healthy tissues, with OX1R positively identified in gastric cancer cell lines (Hu et al., 2020; Wen et al., 2015; Liu et al., 2015). Beyond malignancies, fibroblast-like synoviocytes isolated from non-cancerous sources also express both orexin receptors, suggesting broader physiological roles (Sun et al., 2018). Collectively, these findings indicate a widespread but heterogeneous expression of orexin peptides and receptors in diverse cancer cells/tissues, supporting emerging perspectives on the orexin system’s involvement in tumor biology and potential as a therapeutic target.

## 3 Pharmacokinetics and circulating orexin peptides

### 3.1 Pharmacokinetics

In total, we have found three studies describing the pharmacokinetics of orexins (Ehrstrom et al., 2005a; Ehrstrom et al., 2004; Dhuria et al., 2009). Summary is provided in Table 3.

**TABLE 3:** Comparison of OxA pharmacokinetic parameters across published studies. Values from the publications were converted to uniform units for simplification of comparison. For Ehrström et al., 2004, corrected values estimated from the published figures are presented (see main text).

Publication	Ehrstrom et al., 2005a	Ehrstrom et al., 2004	Dhuria et al., 2009
Species	Human	Rat	Rat
Method of detection	RIA	RIA	Gamma counting of radiolabelled OxA
Method of administration	i.v. infusion	i.v. infusion	i.v. infusion
Dose [nmol/kg]	1.8	3	∼30–50
Infusion duration [mins]	180	30	14
Infusion rate [pmol/kg/min]	10	100	∼2,400–4,000
Cmax (Css) [pmol/L]	∼200	365 ± 41	48,000

C_max_, maximum concentration; Css, steady-state concentration; Vd, apparent volume of distribution; CL, clearance; T½, half-life.

In a clinical study by Ehrström et al. (2005a) involving six healthy male volunteers, OxA was administered as a continuous intravenous infusion at 10 pmol/kg/min for 180 min. Plasma OxA concentrations were assessed by radioimmunoassay without prior sample extraction, which may influence assay specificity. Although the study was not designed as a formal pharmacokinetic investigation and no pharmacokinetic parameters were reported, approximate values can be recovered from the presented concentration–time profiles (calculating methodology described in Supplementary Material). The elimination half-life (t_1_/_2_) of OxA can be estimated at ∼20 min, systemic clearance at ∼50 mL/min/kg and the apparent volume of distribution (Vd) as ∼1.5 L/kg. The average steady-state plasma concentration achieved during infusion was ∼0.7 ng/mL which is an order of magnitude higher than reported endogenous baseline concentrations (0.01–0.1 ng/mL).

Ehrström et al. (2004) also investigated the plasma pharmacokinetics of OxA following intravenous infusion in rats, employing a 30-min constant-rate infusion protocol at 100 pmol/kg/min. Upon discontinuation of the infusion, the reported terminal t_1_/_2_ was 27.1 ± 9.5 min, with an Vd of 0.34 ± 0.17 L/kg and systemic clearance estimated at 8.4 ± 2.2 mL/min/kg. However, the figures and data provided in this study indicate substantial discrepancies in the Vd and clearance values originally reported (calculating methodology described in Supplementary Material). The recalculated Vd values for individual time points fall within the range of 4.2–6.4 L/kg. A t_1_/_2_ between 20 and 30 min corresponds to the originally reported calculations, but a recalculated clearance between 100 and 200 mL/min/kg substantially differs from the originally reported pharmacokinetic results.

Assessment of dose linearity revealed that OxA displayed linear kinetics up to a total dose of 1,500 pmol/kg, supporting first-order elimination within the tested concentration range. Plasma concentrations were measured using a commercially available radioimmunoassay (RIA) kit (code RK-003 30, Phoenix Pharmaceuticals, Belmont, CA, USA), preceded by column extraction, at standardized sampling points post-infusion (10, 20, and 30 min during infusion and at 10, 20, and 30 min following cessation).

In a comparative study of intranasal (i.n.) and intravenous (i.v.) delivery of OxA to rats, Dhuria et al. (2009) reported a markedly prolonged plasma t_1_/_2_ of 135 min following a single 14-min i.v. infusion (36 µg) (Dhuria et al., 2009). Calculated Vd ranged from 0.8 to 1.2 L/kg, while clearance was between 4 and 6 mL/min/kg, although standard deviations were not reported. The study utilized radiolabeled OxA for precise pharmacokinetic measurement from whole blood. Notably, the absolute bioavailability of intranasal OxA was 14%, consistent with significant breakdown prior to systemic absorption.

Importantly, despite reduced systemic exposure following i.n. delivery, both administration routes resulted in comparable concentrations of OxA in multiple brain regions, including the hypothalamus and cortex. This equivalence in central nervous system distribution indicates high blood–brain barrier (BBB) permeability for OxA, as evidenced by few studies employing both radiolabeled compound tracing and direct peptide measurements in brain parenchyma or CSF (Dhuria et al., 2009; Kastin and Akerstrom, 1999; Ogawa et al., 2016; Deadwyler et al., 2007). Kastin and Akerstrom characterized the rapid BBB transit and high lipophilicity of OxA, suggesting an intrinsic propensity for neural tissue penetration (Kastin and Akerstrom, 1999). Several preclinical and clinical investigations have demonstrated CNS uptake of OxA after i.n. administration, verified either by direct bioanalytical measurement of brain OxA concentrations (Dhuria et al., 2009; Van de Bittner et al., 2018) or indirectly through CNS pharmacodynamic effects, including behavioral, neurophysiological, and autonomic effects (Deadwyler et al., 2007; Weinhold et al., 2014; Meusel et al., 2022; Calva et al., 2019; Calva et al., 2018; Sherman et al., 2021; Baier et al., 2011; Baier et al., 2008; Dhuria et al., 2016; Durairaja et al., 2023).

Analysis of pharmacokinetic parameters across three published studies suggests that OxA exhibits a relatively large apparent Vd that may include plasma proteins, blood cells, vascular endothelium, and/or extravascular tissues. Plasma protein binding has been previously demonstrated for OxA (HM et al., 2021), and represents a methodological consideration in both endogenous and exogenous quantification; protein binding may confound accurate measurement of OxA concentrations in blood. The protein binding could explain large Vd in the preclinical study by Ehrstrom et al. (2004) (Ehrstrom et al., 2005a) compared to another study by Dhuria el al. (Dhuria et al., 2009), who utilized radiolabeled OxA and total gamma counting in whole blood, precluding differentiation between the bound and unbound fractions. Pre-analytical procedures for plasma samples, such as protein precipitation or column extraction, may result in variable recovery of OxA depending on its bound fraction (HM et al., 2021). Nonetheless, explanation of the remaining distribution is still needed. As OxA can cross BBB, we can speculate about its possible distribution to the brain as well as to the other tissues, where OxA has been found naturally (see previous paragraph), namely, gastrointestinal system, enteric nervous system and endocrine system. The extensive distribution of OxA likely corresponds with the reported t_1_/_2,_ which is considerably longer than that of other endocrine peptides such as insulin, oxytocin or glucagon-like peptide-1, which have a short t_1/2_ of between 1 and 10 min (Arnolds et al., 2010; Hui et al., 2002; Tanaka et al., 2018). The prolonged t_1_/_2_ of OxA may reflect slow tissue release, protection against proteolytic cleavage by plasma proteins, and stabilization *via* intramolecular disulfide bonds (Saitoh and Sakurai, 2023). The unusually long t_1_/_2_ reported in the study by Dhuria et al. (2009). (130 min) may resulted from quantification of total blood radioactivity, potentially detecting cleaved peptide in addition to intact OxA, despite prior stability validation.

### 3.2 Blood levels of circulating orexin peptides

Another issue is the precision of endogenous OxA blood levels measurement, as literature data show huge inter study variability of up to seven orders of magnitude ranging from 0.1 pg/mL up to 1 μg/mL (El-Sedeek et al., 2010; Lengvenyte et al., 2024). We systematically reviewed the available evidence and found 113 articles that reported measured OxA levels in blood (plasma, serum) (HM et al., 2021; Malendowicz et al., 2011; El-Sedeek et al., 2010; Lengvenyte et al., 2024; Abulaiti et al., 2008; Adam et al., 2002; Akbulut et al., 2014; Akca et al., 2020; Aksu et al., 2009; Alkan et al., 2025; Almasi et al., 2022; Amin et al., 2017; Arihara et al., 2001; Arslan et al., 2022; Atacan et al., 2023; Basoglu et al., 2010; Baykal et al., 2019; Bilir et al., 2015; Bronsky et al., 2011; Bronsky et al., 2007; Busquets et al., 2004; Caproni et al., 2011; Cintron et al., 2017; Cioffi et al., 2020; Coban et al., 2013; Dalal et al., 2001; Fernandez-Rilo et al., 2023; Forte et al., 2022; Gan et al., 2022; Gencer et al., 2019; Gultuna et al., 2024; Guo et al., 2022; Gupta et al., 2014; Haass-Koffler et al., 2021; Hao et al., 2017; Heinonen et al., 2005; Higuchi et al., 2002; Hu et al., 2023; Huang et al., 2021; Chen et al., 2022; Chen et al., 2019; Chen et al., 2016; Chien et al., 2015; Cho et al., 2017; Choi et al., 2020; Igarashi et al., 2003; Jin et al., 2020; Kacinski et al., 2012; Kawada et al., 2004; Kobylinska et al., 2017; Kolodziejski et al., 2018; Kucukali et al., 2014; Lai et al., 2024; Lanuza et al., 2022; Lee et al., 2021; Li et al., 2021; Li et al., 2023; Liao and Yu, 2005; Liu et al., 2020; Lu et al., 2021; Majmudar et al., 2022; Makela et al., 2018; Manzardo et al., 2016; Messina G. et al., 2016; Mishra et al., 2017; Moharami et al., 2022; Monda et al., 2020a; Monda et al., 2020b; Nakashima et al., 2019a; Nakashima et al., 2019b; Nigro et al., 2024; Nishijima et al., 2003; Orum et al., 2024; Ozsoy et al., 2017; Ozturk et al., 2022; Pan et al., 2021; Park et al., 2022; Qin et al., 2023; Razavinia et al., 2021; Razavinia et al., 2019; Ren et al., 2022; Sakurai et al., 2005; Sandikci et al., 2024; Sanchez-de-la-Torre et al., 2011; Saruhan et al., 2022; Shin et al., 2013; Snow et al., 2002; Stawerska et al., 2016; Steward et al., 2019; Sun et al., 2006; Tang et al., 2017; Tohma et al., 2015; Tomasik et al., 2004; Tomasik and Sztefko, 2009; Tsai and Huang, 2018; Tsuchimine et al., 2019; Turkoglu et al., 2020; Unler et al., 2022; Ustabas Kahraman et al., 2015; Wang et al., 2023; Wang ZH. et al., 2014; Yang et al., 2024; Yang et al., 2020; Yilmaz et al., 2013a; Yilmaz et al., 2013b; Yilmaz et al., 2024; You et al., 2022; Yurt et al., 2013; Zhu et al., 2022; Zhu et al., 2011; Zhu et al., 2020; Ziolkowski et al., 2016). Summary of the available studies is in Tables 4 and 5 and complete list is in Supplementary Table S2. Most of the papers used commercially available kits for OxA detection by 20 different companies, in 10 papers kit manufacturer was not specified and there were 3 articles using authors‘ own in-house developed methodology.

**TABLE 4:** Sex and age group distribution in publications reporting different concentrations of Orexin A. Data are presented as the number of studies in the respective sex or age group with respected frequency in brackets.

Range		10–100 ng/mL	1–10 ng/mL	0.1–1 ng/mL	0.01–0.1 ng/mL	0.001–0.01 ng/mL
No. of studies		7	30	30	39	5
Sex N (%)	Female	1 (14%)	3 (10%)	4 (13%)	8 (21%)	0 (0%)
	Male	4 (57%)	4 (13%)	2 (7%)	4 (10%)	1 (20%)
	Both	2 (29%)	22 (73%)	23 (77%)	27 (69%)	4 (80%)
	NS	0 (0%)	1 (3%)	1 (3%)	0 (0%)	0 (0%)
Age N (%)	<1 year	0 (0%)	1 (3%)	0 (0%)	0 (0%)	0 (0%)
	1–10 years	1 (14%)	4 (13%)	4 (13%)	4 (10%)	0 (0%)
	10–18 years	0 (0%)	0 (0%)	2 (7%)	4 (10%)	0 (0%)
	18–40 years	2 (29%)	4 (13%)	4 (13%)	6 (15%)	1 (20%)
	40–65 years	2 (29%)	16 (53%)	18 (60%)	16 (41%)	3 (60%)
	>65 years	2 (29%)	3 (10%)	0 (0%)	7 (18%)	1 (20%)
	NS	0 (0%)	2 (7%)	2 (7%)	2 (5%)	0 (0%)
Year of publication mean ± SD		2017 ± 6	2018 ± 4	2019 ± 6	2014 ± 7	2014 ± 9

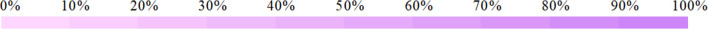

The summary of individual studies is provided in Supplementary Table S1.

NS, not specified.

**TABLE 5:** The distribution of different methodologies in publications reporting different concentrations of Orexin A. Data are presented as number of studies with respective methodologies reported within each category with frequency in brackets.

Range		10–100 ng/mL	1–10 ng/mL	0.1–1 ng/mL	0.01–0.1 ng/mL	0.001–0.01 ng/mL
No. of studies		7	30	30	39	5
Method	ELISA	7 (100%)	28 (93%)	21 (70%)	15 (38%)	2 (40%)
	RIA	0 (0%)	0 (0%)	2 (7%)	22 (56%)	3 (60%)
	Multi	0 (0%)	0 (0%)	4 (13%)	0 (0%)	0 (0%)
	NS	0 (0%)	2 (7%)	3 (10%)	2 (5%)	0 (0%)
Extraction	Rep.	0 (0%)	2 (7%)	5 (17%)	21 (54%)	3 (60%)
	Not Rep.	7 (100%)	28 (93%)	25 (83%)	18 (46%)	2 (40%)
Acidification of sample	Rep.	0 (0%)	0 (0%)	21 (7%)	13 (33%)	3 (60%)
	Not Rep.	7 (100%)	30 (100%)	28 (93%)	26 (67%)	2 (40%)
Use of protease inhibitor	Rep.	1 (14%)	4 (13%)	3 (10%)	12 (31%)	2 (40%)
	Not Rep.	6 (86%)	26 (87%)	27 (90%)	27 (69%)	3 (60%)
Immediate processing of samples	Rep.	0 (0%)	6 (20%)	5 (17%)	9 (23%)	2 (40%)
	Not Rep.	7 (100%)	24 (80%)	25 (83%)	30 (77%)	3 (60%)
Sample type	Plasma	2 (29%)	14 (47%)	8 (27%)	27 (69%)	3 (60%)
	Serum	5 (71%)	16 (53%)	22 (73%)	12 (31%)	2 (40%)

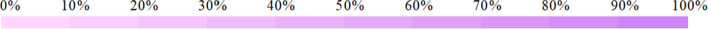

The summary of individual studies is provided in Supplementary Table S1.

NS, not specified; Rep., reported; Multi, multiplex assay.

To facilitate interpretation, studies were stratified into seven categories based on the range of reported OxA concentrations. Specifically, one study reported OxA concentration in the range of 1,000–10,000 ng/mL, seven studies reported OxA levels between 10 and 100 ng/mL, thirty in 1–10 ng/mL, thirty studies in 0.1–1 ng/mL, thirty-nine studies reported concentrations within 0.01–0.1 ng/mL, five studies fell within 0.001–0.01 ng/mL, and one study reported levels between 0.0001 and 0.001 ng/mL. Two extreme outlier studies were excluded from both ends of the spectrum, as these values likely reflect unit conversion errors rather than true physiological or methodological variability (El-Sedeek et al., 2010; Lengvenyte et al., 2024). First, we examined sex and age as potential sources of variability. Neither age group nor sex predominated within any specific concentration range, with both factors being similarly distributed across all ranges (Table 4). Among the seven studies corresponding to the highest range (10–100 ng/mL), four were conducted in male subjects (57%), indicating a slightly higher representation of males compared with other ranges. However, given the limited number of studies in this category and the absence of any consistent pattern across lower ranges, there is no clear association between OxA levels and sex.

Further, disease status was evaluated as a potential source of variability of OxA levels. From all the reviewed studies only one study reported more than a tenfold difference in OxA concentrations between healthy and disease groups. This study compared OxA levels in obese individuals and healthy controls (Heinonen et al., 2005). OxA levels in the obese subjects were significantly elevated relative to the normal-weight control group (75.3 ± 24.1 pg/mL vs. 0.8 ± 0.4 pg/mL, *p* < 0.0001). In contrast, six other studies assessing obese and normal-weight groups did not observe such pronounced differences (equal or more than tenfold) (Bronsky et al., 2007; Hao et al., 2017; Kawada et al., 2004; Kolodziejski et al., 2018; Liao and Yu, 2005; Mishra et al., 2017). Two other studies reported difference between studied groups close to tenfold (never smoker obstructive sleep apnoea patients vs. never smoker healthy control group, 4.0 ± 1.2 vs. 32.9 ± 9.5 and bipolar depression patients vs. healthy control group 107.64 ± 130.63 946.74 ± 1,268.66) (Aksu et al., 2009; Unler et al., 2022). Since no other study found such pronounced difference between healthy and disease group, we believe that diseases are not key contributors to wide range of OxA levels and may contribute to the observed between study variability only to a minor extent.

From several studies, in which it was possible to estimate individual measured values, we could estimate a maximum difference of approximately 20 times between highest and lowest concentrations measured in the respective studies (Malendowicz et al., 2011; Haass-Koffler et al., 2021; Chen et al., 2022; Choi et al., 2020; Kucukali et al., 2014; Makela et al., 2018; Vilke et al., 2019).

Another possible explanation could be variation in levels during the day such as seen in for other hormones (Makela et al., 2018). Only a single study directly addressed this issue with no finding of any pattern (Makela et al., 2018). However, given that this conclusion is based on one study alone, a contribution of daily fluctuations in release to the observed variability cannot be excluded.

Additionally, we analyzed potential associations between methodology and reported OxA values. In the lower concentration ranges RIA was more frequent method compared to ELISA (Table 5). Furthermore, there was also a noticeable trend towards more frequent reporting of sample extraction, sample acidification, or the use of protease inhibitors. This trend may be attributable to the OxA binding to plasma proteins, since extraction methodologies and acidification remove plasma proteins from samples. Consequently, OxA may be co-precipitated with proteins and not released as intended, leading to underestimation of its concentration. Such peptide behavior, including that of OxA, has been previously documented (HM et al., 2021; Kohler et al., 2024). As most RIA kit protocols included acidification step, the associations for RIA and sample preanalytical procedures are not independent. Interestingly, one RIA in house methodology, which reported one of the lowest concentrations of OxA, concluded OxA recovery of around 60% (Makela et al., 2018).

ELISA theoretically has the capacity to measure both free and protein-bound fractions of OxA; however, it may overestimate OxA concentrations due to nonspecific binding by OxA antibodies. A similar limitation applies to RIA, as OxA antibodies are also used within the RIA protocol, potentially contributing to overestimation or inaccurate quantification in both assay types. Indeed, it has been shown that LC-MS/MS-based quantification of OxA invariably yielded lower concentrations than RIA by a factor of up to five, as immunoassays may detect not only intact OxA but also metabolites or precursor forms (Abulaiti et al., 2008). Furthermore, the RIA’s reported precision was poor at low concentrations, compounding uncertainty at the low (endogenous) range relevant for physiological interpretation (Abulaiti et al., 2008).

Preanalytical handling (acidification, extraction, declaration of immediate sample processing or use of protease inhibitors seem to be more prevalent in lower range categories. While it is not surprising to find acidification and extraction in lower range categories (see previous paragraph), given the relatively short t_1_/_2_ of OxA as established in pharmacokinetic studies, it would be anticipated that reports of immediate sample processing, use of protease inhibitors and the utilization of plasma samples would be more prevalent in the higher concentration categories; however, such a pattern was not observed. Possible explanation lies again in RIA protocols where such steps (immediate processing, use of protease inhibitors and utilisation of plasma samples) are recommended thus not surprising to find in lower range categories where RIA is also abundant.

In conclusion, the substantial discrepancies observed in reported OxA blood concentrations are primarily attributable to inconsistencies in study methodologies that detect both bound and unbound fractions, as well as protein losses during the pre-analytical phase. These factors are important because OxA has a relatively high binding affinity to plasma proteins. The pre-analytical factors introduce greater variability and bias in measured OxA levels in serum and plasma than the potential lack of specificity of immunoassay techniques. If we accept that RIA kits (because of their preanalytical handling protocols) often measure primarily the unbound fraction of OxA (i.e., the fraction not precipitated with plasma proteins), then based on their reported concentration ranges we can estimate that the unbound fraction accounts for roughly 10–100 pg/mL in plasma or serum. Applying similar reasoning to ELISA kits, which more commonly measure both bound and unbound OxA, the estimated total plasma/serum concentration would fall in the range of approximately 0.1–10 ng/mL.

**FIGURE 5:**
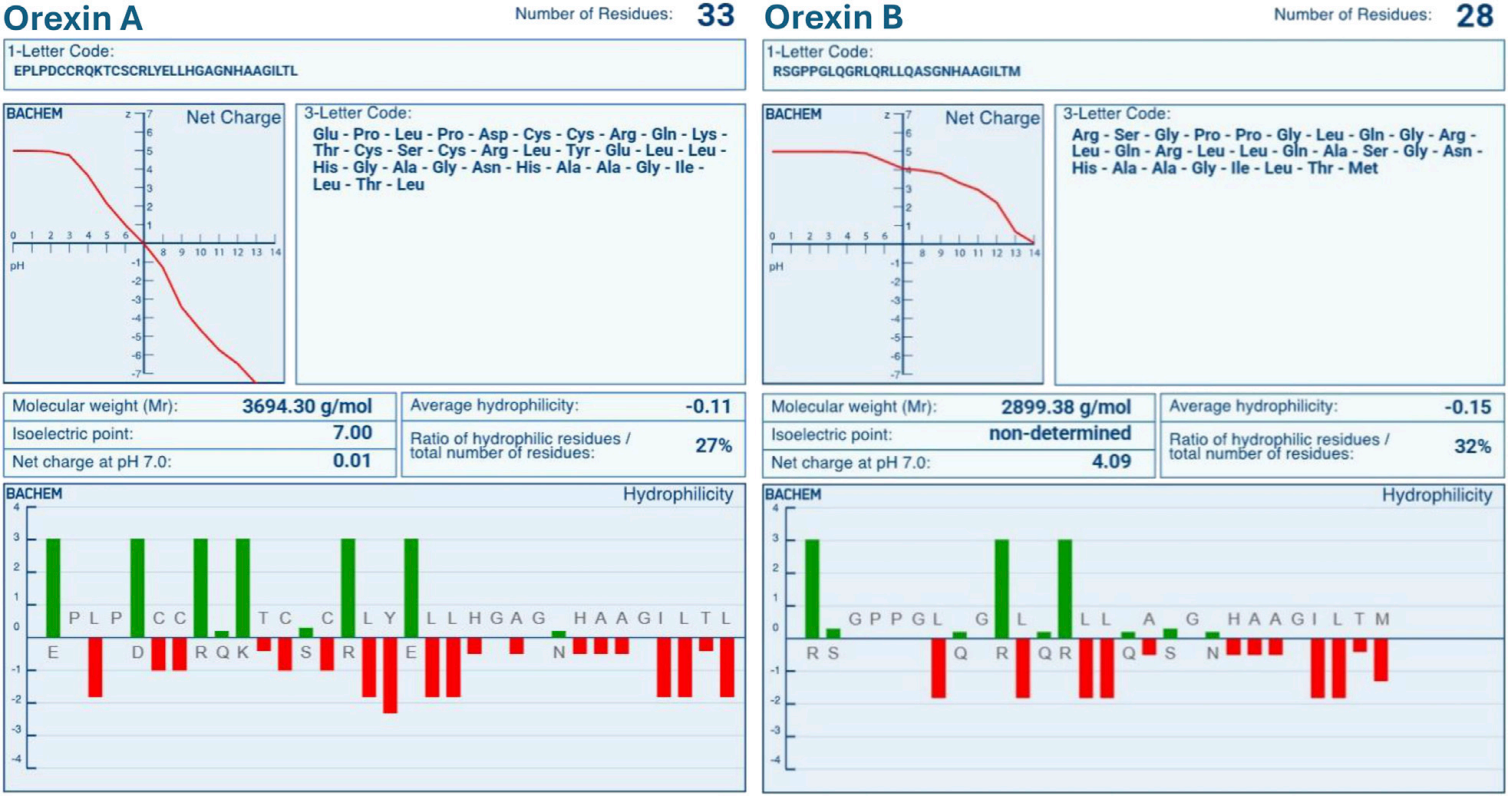
Predicted physicochemical characteristics of orexin peptides. Values were obtained using the Bachem Peptide Calculator (https://www.bachem.com/knowledge-center/peptide-calculator/; accessed 11 October 2025). The calculation for orexin A includes the pyroglutamyl modification of the N-terminal glutamine residue.

Limited evidence suggests that OxB exhibits high degradability in blood and does not readily cross BBB (Kastin and Akerstrom, 1999). In contrast to OxA, OxB lacks disulfide bonds, which are thought to confer structural stability to OxA (Saitoh and Sakurai, 2023). Theoretically, based on its amino acid composition, OxB possesses a net positive charge compared to the neutral OxA, which is anticipated to impede its ability to cross physiological membranes (BACHEM. Peptide Calculator, 2025) (Figure 5). There are five published studies in the literature that measured OxB levels in blood utilizing assay kits from three different manufacturers (Malendowicz et al., 2011; Baykal et al., 2019; Caproni et al., 2011; Tomasik et al., 2004; Wang et al., 2023). The reported OxB levels varied considerably with values ranging from 1 to 10 pg/mL (Baykal et al., 2019) to 10–100 ng/mL (Malendowicz et al., 2011).

## 4 Hypothalamic-pituitary-adrenal (HPA) axis and corticoid secretion

The central effect of orexin on stress, on the HPA axis, and especially on adrenocorticotropic hormone (ACTH) release has been well documented (Lawrence, 2017). Interestingly, in patients with narcolepsy, basal ACTH levels were found to be reduced, whereas pulsatile ACTH secretion persisted. In contrast, cortisol secretion remained unchanged when compared to healthy controls (Kok et al., 2002).

Although functional data and data from animal studies are supportive for orexin system presence in pituitary gland and involvement in regulation of hormone secretion (Kaminski and Smolinska, 2012; Messina A. et al., 2016; Kok et al., 2002; Kok et al., 2004), in humans we have found only three studies with contradictory findings regarding presence of orexin peptides or orexin receptors in the gland (Blanco et al., 2001; Blanco et al., 2003; Arihara et al., 2000) (Table 1). Two studies by Blanco et al. (2001), Blanco et al. (2003) report presence of OxA, OxB, OX1R and OX2R in the anterior pituitary while one study by Arihara et al. (2000) reports no finding of OxA in the pituitary yet not contradicting the receptor presence. The Human Protein Atlas data support the presence of prepro-orexin, OX1R, and OX2R in pituitary endocrine cells based on single-cell transcriptomic analyses (Karlsson et al., 2021; The Human Protein Atlasa; The Human Protein Atlasb; The Human Protein Atlasc), whereas tissue-level transcriptomics support only OX1R expression (Uhlen et al., 2015; The Human Protein Atlasa; The Human Protein Atlasb; The Human Protein Atlasc), and proteomic data do not confirm the presence of any of these components (Uhlen et al., 2015; The Human Protein Atlasa; The Human Protein Atlasb; The Human Protein Atlasc).

Ten publications have investigated the effects of intracerebroventricular administration of OxA on plasma corticosterone or ACTH levels in rodents (Russell et al., 2001; Al-Barazanji et al., 2001; Ito et al., 2008; Moreno et al., 2005; Kuru et al., 2000; Jaszberenyi et al., 2000; Ida et al., 2000; Brunton and Russell, 2003; Chang et al., 2007; Deutschman et al., 2013). In addition, two studies have reported on the effects of OxB using similar measures (Kuru et al., 2000; Jaszberenyi et al., 2000). In summary seven studies found an increased ACTH plasma level after administration of OxA (doses from 0.5 up to 30 µg/animal) (Russell et al., 2001; Al-Barazanji et al., 2001; Moreno et al., 2005; Kuru et al., 2000; Brunton and Russell, 2003; Chang et al., 2007; Deutschman et al., 2013), six studies reported an increase in plasma corticosterone levels after administration of OxA (doses from 0.1 up to 30 µg/animal) (Russell et al., 2001; Moreno et al., 2005; Kuru et al., 2000; Jaszberenyi et al., 2000; Ida et al., 2000; Brunton and Russell, 2003), one study found that selective corticotropin-releasing hormone antagonist abolished OxA and OxB evoked corticosterone release (Jaszberenyi et al., 2000), and one study did not find altered ACTH plasma level after administration of OxA (0.05 and 0.5 µg/animal) (Ito et al., 2008). In one study intracerebroventricular administration of OX2 antagonist together with OxA decreased ACTH plasma levels compared to administration of OxA alone although remained still increased compared to administration of vehicle alone (Chang et al., 2007). Decreased secretion of ACTH upon stress stimuli in OX2R KO mice and mice treated with OX2R antagonist or DORA was also described (Yun et al., 2017). Three studies did not find increased plasma corticosterone levels after administration of OxA (doses from 0.01 up to 0.5 µg/animal) (Ito et al., 2008; Kuru et al., 2000; Jaszberenyi et al., 2000). Two studies reported increase in plasma corticosterone levels after administration of OxB in a dose dependent manner (First study found increase corticosterone after dose of 30 µg/animal but not for doses 0.3 and 3 µg/animal, while second study found increase of corticosterone after doses of 0.4, 0,8 and 1.6 µg/animal but not for 0.08 µg/animal) (Kuru et al., 2000; Jaszberenyi et al., 2000).

There are also five publications describing the effects of subcutaneous administration of OxA (Ogawa et al., 2016; Malendowicz et al., 1999; Malendowicz et al., 2001a; Nowak et al., 2000; Malendowicz et al., 2001b) and four studies describing the effects of subcutaneously administered OxB (Malendowicz et al., 1999; Malendowicz et al., 2001a; Nowak et al., 2000; Malendowicz et al., 2001b) on plasma corticosterone or ACTH levels in rodents. OxA increased ACTH plasma levels in one study (36 μg/kg/rat) (Ogawa et al., 2016; Nowak et al., 2000) but another study (71 μg/kg/rat) reported no effect (Malendowicz et al., 2001a). Nevertheless, OxA increased corticosterone plasma levels in all five studies (after doses of 2 mg/mice, 18 μg/kg/rat, 36 μg/kg/rat and 71 μg/kg/rat) (Ogawa et al., 2016; Malendowicz et al., 1999; Malendowicz et al., 2001a; Nowak et al., 2000; Malendowicz et al., 2001b). OxB did not affect plasma ACTH levels in two study (29 μg/kg/rat and 60 μg/kg/rat) (Malendowicz et al., 2001a; Nowak et al., 2000), but it did increase plasma levels of corticosterone at 29 μg/kg/rat and 60 μg/kg/rat though not at dose of 14.5 μg/kg/rat (Malendowicz et al., 1999; Malendowicz et al., 2001a; Nowak et al., 2000; Malendowicz et al., 2001b).

In one study intraperitoneal (i.p.) administration of 1 µg OxA or 0.8 µg OxB did not increase corticosterone levels in rats (Jaszberenyi et al., 2000). Regarding sex differences no study directly compared ACTH or corticosterone levels in male and female animals after OxA or OxB exposure. Interestingly, one study found no effect on ACTH or corticosterone secretion after intracerebroventricular administration of 0.5 µg OxA in pregnant rats while in virgin rats produced significant increase of ACTH and corticosterone levels (Brunton and Russell, 2003). Summary of these publications is presented in Table 6.

**TABLE 6:** Comparison of *in vivo* studies describing effect of orexins on HPA axis.

Publication	Species (sex)	Method of administration and dose per animal			Repeated dosing Y/N	Effect on ACTH OxA/OxB		Effect on corticosterone OxA/OxB	
		​	OxA	OxB					
^1^ Kuru et al., 2000	Rat (M)	i.c.v.	0.3 µg 3 µg 30 µg	0.3 µg 3 µg 30 µg	No	— **↑** —	— — —	N ↑ ↑	N N ↑
^1^ Ida et al.,2000	Rat (M)	i.c.v.	10 µg 30 µg	X	No	— —		↑ ↑	
Russel et al., 2001	Rat (M)	i.c.v.	10 µg	X	No	↑		↑	
^3^ Jaszberenyi et al., 2000	Rat (M)	i.c.v.	0.01 µg 0.1 µg 1.5 µg 1 µg	0.08 µg 0.4 µg 0.8 µg 1.6 µg	No	— — — —	— — — —	N ↑ ↑ ↑	N ↑ ↑ ↑
Al—Barazanji et al., 2001	Rat (F)	i.c.v.	10 µg 30 µg	X	No	↑ ↑		— —	
Brunton et al., 2003	Rat (F)	i.c.v.	0.5 µg	X	No	↑^a^		↑^a^	
Moreno et al.,2005	Rat (F)	i.c.v.	1 µg	X	No	↑		↑	
Chang et al., 2007	Rat (M)	i.c.v.	3 µg	X	No	↑		—	
Ito et al., 2008	Mouse (M)	i.c.v.	0.05 µg 0.5 µg	X	No	N N		N N	
Deutschman et al., 2013	Mouse (M)	i.c.v.	11 µg	X	No	↑		—	
^2^ Malendowicz et al., 1999	Rat (F)	s.c.	18 μg/kg 36 µg//kg	14.5 μg/kg 29 µg//kg	No	— —	— -	↑ ↑	N ↑
^2^ Nowak et al., 2000	Rat (F)	s.c.	36 µg//kg	29 µg//kg	No	↑	N	↑	↑
^2^ Malendowicz et al., 2001a	Rat (F)	s.c.	71 μg/kg	60 μg/kg	Yes (7days)	N	N	↑	↑
^2^ Malendowicz et al., 2001b	Rat^b^(F)	s.c.	36 µg//kg	29 µg//kg	Yes^c^	—	—	↑	N
Ogawa et al., 2016	Mouse (M)	s.c.	2 mg	X	No^d^	—		↑	
^3^ Jaszberenyi et al., 2000	Rat (M)	i.p.	1 µg	0.8 µg	No	—		N	

i.c.v., intraventricular; s.c., subcutaneous; OxA, orexin A; OxB, orexin B; —, Not tested; N, No change; F, Female; M, Male.

^a^ No effect in pregnant rats.

^b^ Immature rats.

^c^ Three injections 28, 16 and 4h before being sacrificed.

^d^ Dose was administrated *via* s.c. catheter in course of 24 h. Indexed number placed to the left of each reference citation marks same identified collaboration group for the studies with same index number.

All together this data strongly supports that both centrally administrated and peripherally administrated orexin peptides (particularly OxA) exert significant effect on corticosteroid secretion. Since at least OxA was demonstrated to be able to cross BBB (Kastin and Akerstrom, 1999) solely based on these data we cannot conclude whether is this effect mediated centrally through HPA axis regulation on peripherally *via* direct effect on cells in adrenal gland. It was previously demonstrated that central effect could also act *via* sympathetic system as in one study intracerebral administration of OxA increased adrenal sympathetic nerve activity. This effect was abolished by a nonselective orexin receptor antagonist (Korim et al., 2014).

To address this question, a closer examination of the adrenal gland and its cellular composition is required. In 1999, Malendowicz et al. demonstrated that both OxA and OxB directly stimulate glucocorticoid secretion from the rat adrenal cortex, i.e., independently of the HPA axis and ACTH (Malendowicz et al., 1999). Later, the same effect was observed in human adrenal glands (Mazzocchi et al., 2001a; Randeva et al., 2001). The exact mechanism behind receptor activation and signal transduction is challenging, and yet remains to be elucidated. The examination of the anatomical distribution of OX1R, OX2R, prepro-orexin, OxA, and OxB within the adrenal gland reveals considerable inconsistency among published reports. Summary of these papers is provided in Table 7.

**TABLE 7:** Comparison of studies investigating presence of OX1R, OX2R, prepro-orexin, OxA or OxB in adrenal gland or adrenal cortex derived cancer cells.

Publication	Species (Sex)	Source	Method	OX1R		OX2R		Prepro-orexin		OxA		OxB	
				Medulla	Cortex	Medulla	Cortex	Medulla	Cortex	Medulla	Cortex	Medulla	Cortex
^1^ Lopez et al. 1999	Rat (M)	Adrenal gland	PCR, IH	O	X	O	X	X	X	​	​	​	​
^2^ Nanmoku et al. 2000	Rat (NS)	Adrenal gland	NB	X	X	O	O	X	X	​	​	​	​
^3^ Malendowicz et al. 2001a	Rat (F)	Adrenal gland	PCR	X	O	X	O	​	​	​	​	​	​
^3^ Malendowicz et al. 2001b	Rat (F)	Adrenal gland	PCR	X	O	X	O	​	​	​	​	​	​
^4^ Randeva et al. 2001	Human (NS)	Adrenal gland	PCR, FISH, IH	X	X	X	O	​	​	​	​	​	​
^1^ Blanco et al. 2002	Human (B)	Adrenal gland	IH	X	O	O	X	​	​	​	​	​	​
^2^ Nanmoku et al. 2002	Pig (NS)	Adrenal medulla and cortex cells	SB (after PCR)	O	O	​	​	​	​	​	​	​	​
Kawada et al. 2003	Cattle (NS)	Adrenal gland	PCR	O	​	X	​	​	​	​	​	​	​
^3^ Spinazzi et al. 2005a	Human (NS)	Adrenal gland	PCR, WB	​	O	​	O	​	X	​	X	​	X
^3^ Spinazzi et al. 2005b	Rat (M)	Adrenal gland	PCR	​	O	​	O	​	​	​	​	​	​
^3^ Ziolkowska et al. 2005	Human, Rat (NS)	Adrenal gland	PCR	​	O	​	O	​	​	​	​	​	​
Mitsukawa et al. 2022	Rat (M)	Adrenal gland	RL	​	​	X	O	​	​	​	​	​	​

Unspecified adrenal tissue				OX1R		OX2R		Prepro-orexin		OxA		OxB	
^5^ Arihara et al. 2000	Human (B)	Adrenal gland and adenomas	IH	​		​		​		X		​	
^1^ Lopez et al. 2001	Rat (M)	Adrenal gland	PCR	O		O		​		​		​	
^4^ Randeva et al. 2001	Human (NS)	Adrenal gland	WB	X		O		O		O		​	
^4^ Karteris et al. 2001	Human (NS)^a^	Adrenal gland	PCR, WB, FISH, IF	X		O		O		O		​	
^4^ Johren et al. 2003	Rat (B)	Adrenal gland	PCR	O		O		​		​		​	
^5^ Nakabayashi et al. 2003	Human (B)	Adrenal gland	IH: OxA, PCR: ppOx	​		​		O		X		​	
^4^ Karteris et al. 2004	Rat (M)	Adrenal gland	PCR	O		O		X		​		​	
Zhang et al. 2005	Sheep (M)	Adrenal gland	PCR	O		X		​		​		​	
^3^ Szyszka et al. 2015	Human (NS)	Adrenal gland	PCR	O		O		​		​		​	

Cortex derived cancer cells				OX1R		OX2R		Prepro-orexin		OxA		OxB	
^1^ Blanco et al. 2002	Human (B)	Adrenocortical adenoma	IH	O		X		​		​		​	
^3^ Spinazzi et al. 2005a	Human (NS)	Adrenocortical adenoma	PCR, WB	O		O		O		O		X	
^4^ Ramanjaneya et al. 2008	Human (F)	Human H295R cell line	PCR, WB	O		O		​		​		​	
^4^ Wenzel et al. 2009	Human (F)	Human H295R cell line	PCR	X		O		​		​		​	
Chang et al. 2014	Human (F)	Human H295R cell line	PCR	O		X		​		​		​	
Soejima et al. 2023	Human (F)	Human H295R cell line	PCR	O		O		​		​		​	

B, Both sexes; F, Females; M, Males; NS, Not specified; FISH, Fluorescence *in situ* hybridization; IH, Immunohistochemistry; NB, Northern blot; PCR, Polymerase chain reaction; ppOx, prepro-orexin; RL, Radiolabelling; SB, Southern blot; WB, Western blot; OxA, orexin A; OxB, orexin B; OX1R, Orexin receptor type 1; OX2R, Orexin receptor type 2; ppOX, pre-proorexin; X, Not investigated.

^a^ Fetal adrenal gland. Indexed number placed to the left of each reference citation marks same identified collaboration group for the studies with same index number.

Regardless species and sex, seven papers report OX1R localisation in cortex of adrenal gland (Blanco et al., 2002; Malendowicz et al., 2001a; Malendowicz et al., 2001b; Spinazzi et al., 2005a; Nanmoku et al., 2002; Ziolkowska et al., 2005), while three papers report did not confirm OX1R presence in the cortex (Randeva et al., 2001; Lopez et al., 1999; Nanmoku et al., 2000). Eight papers found OX2R in cortex (Randeva et al., 2001; Malendowicz et al., 2001a; Malendowicz et al., 2001b; Spinazzi et al., 2005a; Ziolkowska et al., 2005; Nanmoku et al., 2000; Mitsukawa and Kimura, 2022; Spinazzi et al., 2005b; Kawada et al., 2003), while two papers did not (Blanco et al., 2002; Lopez et al., 1999). Similar situation is in medulla, three papers confirmed OX1R presence (Nanmoku et al., 2002; Lopez et al., 1999; Kawada et al., 2003), while five papers did not (Randeva et al., 2001; Blanco et al., 2002; Malendowicz et al., 2001a; Malendowicz et al., 2001b; Nanmoku et al., 2000). Regarding OX2R, three papers reported OX2R in the medulla (Blanco et al., 2002; Lopez et al., 1999; Nanmoku et al., 2000), while five papers did not confirm its presence (Randeva et al., 2001; Malendowicz et al., 2001a; Malendowicz et al., 2001b; Mitsukawa and Kimura, 2022; Kawada et al., 2003). Five studies described OX1R in unspecified adrenal tissue (Randeva et al., 2001; Karteris et al., 2004; Szyszka et al., 2015; Lopez et al., 2001; Johren et al., 2003; Zhang et al., 2005), similarly as six papers reported OX2R (Randeva et al., 2001; Karteris et al., 2004; Szyszka et al., 2015; Lopez et al., 2001; Johren et al., 2003; Karteris et al., 2001), while two studies could not confirm the presence of OX1R (Randeva et al., 2001; Karteris et al., 2001) and one study OX2R (Zhang et al., 2005). Five studies found OX1R in human cortical adenomas or human cell line H295R derived from adrenal cortex (Blanco et al., 2002; Spinazzi et al., 2005a; Soejima et al., 2023; Ramanjaneya et al., 2008; Chang et al., 2014) and four studies reported OX2R presence (Spinazzi et al., 2005a; Soejima et al., 2023; Ramanjaneya et al., 2008; Wenzel et al., 2009). One study did not find any presence of OX1R (Wenzel et al., 2009) and two studies did not find any presence of OX2R (Blanco et al., 2002; Chang et al., 2014) in H295R cell line.

Taking together, only one study contradicted presence of either OX1R or OX2R in cortex (Lopez et al., 1999) ten other studies reported either presence of one of them or both (Randeva et al., 2001; Blanco et al., 2002; Malendowicz et al., 2001a; Malendowicz et al., 2001b; Spinazzi et al., 2005a; Nanmoku et al., 2002; Ziolkowska et al., 2005; Nanmoku et al., 2000; Mitsukawa and Kimura, 2022; Spinazzi et al., 2005b; Kawada et al., 2003). Similarly, all papers investigating presence of these receptors in unspecified adrenal tissue or cortex derived cancer cell lines found at least one of these receptors or both (Randeva et al., 2001; Karteris et al., 2004; Szyszka et al., 2015; Spinazzi et al., 2005a; Lopez et al., 2001; Johren et al., 2003; Zhang et al., 2005; Karteris et al., 2001; Soejima et al., 2023; Ramanjaneya et al., 2008; Chang et al., 2014; Wenzel et al., 2009). Data from The Human Protein Atlas (THPA) reports the presence of OX1R supported by tissue proteomic and transcriptomic data (Uhlen et al., 2015; The Human Protein Atlasc); however, this finding is not confirmed by single-cell transcriptomic analyses (Karlsson et al., 2021; The Human Protein Atlasc). In contrast THPA data do not support the presence of OX2R or prepro-orexin in the human adrenal gland based on tissue proteomic, transcriptomic, or single-cell transcriptomic analyses (Uhlen et al., 2015; Karlsson et al., 2021; The Human Protein Atlasa; The Human Protein Atlasb).

The presence of orexin receptors in adrenal gland and their functional significance is further supported by the direct effect of OxA or OxB on corticosteroid secretion from adrenocortical cells, where the literature provides a more consistent and coherent picture. Although one study reported no direct effect of either OxA or OxB on corticosterone secretion from isolated rat adrenal glands (Jaszberenyi et al., 2000), nine independent studies support the involvement of OX1R as the primary receptor mediating direct effect on corticosteroid secretion (Mazzocchi et al., 2001a; Guo X. et al., 2024; Malendowicz et al., 1999; Nanmoku et al., 2002; Ziolkowska et al., 2005; Soejima et al., 2023; Ramanjaneya et al., 2008; Chang et al., 2014; Ramanjaneya et al., 2009), while only two studies with weaker level of evidence identified OX2R (Randeva et al., 2001; Wenzel et al., 2009). Comparison of these studies with respected evidence for their conclusions is presented in Table 8.

**TABLE 8:** Comparison of *in vitro* studies describing effect of orexins on direct corticosteroid release.

Publication	Species	Source	Receptor responsible for the corticosteroid release	Findings
Malendowicz et al., 1999	Rat	Adrenal gland	OX1R	More intense effect on corticosteroid secretion with OxA (nonselective OXR agonist) than OxB (OX2R selective agonist).
Mazzocchi et al., 2001a	Human	Adrenal gland	OX1R	OxA (nonselective OXR agonist) but not OxB (OX2R selective agonist) increased cortisol secretion from dispersed cultured cells.
Nanmoku et al., 2002	Pig	Primary adrenal medulla and cortex cells	OX1R (week evidence)	OxA increased cortisol secretion in porcine adrenal cortex cells. OxB not tested. OX1R mRNA found but OX2R mRNA not tested.
Ziolkowska et al., 2005	Human	Adrenal gland	OX1R	OxA (nonselective OXR agonist) but not OxB (OX2R selective agonist) increased cortisol secretion from dispersed cultured cells.
Ramanjaneya et al., 2008	Human	Human adrenocortical carcinoma H295R cell line	OX1R	Increased expression and production of StAR (Steroidogenic acute regulatory protein) after OxA or OxB treatment was abolished by addition of SORA1 (SB334867).
Ramanjaneya et al., 2009	Human	Human adrenocortical carcinoma H295R cell line	OX1R	Increased activation of ERK1/2 and p38 after OxA or OxB treatment was abolished by addition of SORA1 (SB334867), OxA but not OxB resulted in significant increase in cortisol secretion.
Chang et al., 2014	Human	Human adrenocortical carcinoma H295R cell line	OX1R	SORA1 (SB674042) abolished increased secretion of cortisol after OxA treatment.
Soejima et al., 2023	Human	Human adrenocortical carcinoma H295R cell line	OX1R (week evidence)	Treatment with adrenocortical bone morphogenetic protein (BMP) that induces steroidogenesis downregulated the expression of OX1R but not that of OX2R whereas OxA supressed BMP-receptor signalling pathway (evidence for mutual fine tuning). OxB not tested.
Guo et al., 2024a	Human	Human adrenocortical carcinoma H295R cell line	OX1R	SORA1 (SB334867) abolished increased secretion of cortisol and protein changes in cells after OxA treatment.
Randeva et al., 2001	Human	Adrenal gland	OX2R (week evidence)	Only OX2R found. OxA increased cAMP release and IP3 accumulation.
Wenzel et al., 2009	Human	Human adrenocortical carcinoma H295R cell line	OX2R (OX1R)	Much higher expression of OX2R than OX1R. Downregulation of OX2R after OxA exposure. Contrarily OxA exhibited greater effect on mRNA of steroidogenesis genes than OxB (almost 10X lower EC50).

OxA, orexin A; OxB, orexin B; OX1R, Orexin receptor type 1; OX2R, Orexin receptor type 2.

Together these data strongly support presence and functional importance of OX1R in adrenal gland. While OX2R presence is also possible, the evidence for functional significance regarding corticosteroid secretions is much weaker. Important question is how to explain and interpret observed discrepancy of receptor distribution in literature. It does not appear to be attributable to sex or species differences as no consistent pattern emerges from the available data, however it is conceivable that the presence of alternative orexin receptor variants, arising from genomic sequence diversity or differential splicing, may underlie these inconsistencies. Indeed, the existence of such splice variants with tissue-specific expression has already been documented previously (Chen and Randeva, 2004; Chen et al., 2006; Chen and Randeva, 2010). If an alternative splice variant is expressed in the adrenal gland, it may escape detection by polymerase chain reaction when primers are designed to amplify the hypothalamic isoform. However, depending on sequence similarity and primer design, cross-detection of different splice variants of OX1R or OX2R, or even previously uncharacterized receptor variants, remains theoretically possible. Using direct sequencing of PCR-amplified prepro-orexin from hypothalamus, stomach, colon, and kidney samples, one study confirmed that the sequence was identical to the registered *Homo sapiens* prepro-orexin mRNA sequence identified in the hypothalamus (Nakabayashi et al., 2003). However, this finding does not exclude the possibility of tissue-specific differences at the receptor level in other tissues. Similar issue may arise with antibodies used in immunohistochemistry or Western blotting, where minor structural alterations in the protein can, but do not necessarily have to, impair antibody binding. Finally, tissue-specific homo- or heterodimerization of these receptors, as previously reported, could further contribute to the observed variability (Chen et al., 2015; Ellis et al., 2006; Wang C. et al., 2014; Jantti et al., 2014; Wan et al., 2020; Zhang et al., 2022). The potential existence of alternative variants of prepro-orexin or OxA peptides may also have important pharmacological implications. If such variants exhibit tissue-specific expression or functional differences, they could represent attractive therapeutic targets. Selective modulation of peripheral variants of the orexin system could allow pharmacological intervention in inflammatory or metabolic processes while minimizing undesired central nervous system effects, or conversely enable central modulation without affecting peripheral physiology.

Situation is much more unclear regarding the production of orexin peptides. Only three studies describe the presence of prepro-orexin or OxA in unspecified human adrenal tissue (Randeva et al., 2001; Nakabayashi et al., 2003; Karteris et al., 2001) and one study in human adrenocortical adenoma (Spinazzi et al., 2005a). One study did not find OxA presence in human adrenal cortex (Spinazzi et al., 2005a) and two studies did not find OxA presence in unspecified human adrenal tissue (Nakabayashi et al., 2003; Arihara et al., 2000). Four studies did not find any presence of prepro-orexin in human and rat adrenal gland (one study investigated only cortex) (Karteris et al., 2004; Spinazzi et al., 2005a; Lopez et al., 1999; Nanmoku et al., 2000). We did not find any study, which would confirm presence of OxB in adrenal glands of any species, including human adrenal gland or human adrenocortical adenoma with one study explicitly contradicting such finding (Spinazzi et al., 2005a). Data from The Human Protein Atlas do not support the presence of prepro-orexin in the human adrenal gland based on tissue proteomic, transcriptomic, or single-cell transcriptomic analyses (Uhlen et al., 2015; Karlsson et al., 2021; The Human Protein Atlasa). It is currently difficult to draw a definitive conclusion as to whether OxA is produced in the adrenal gland, as the available evidence remains conflicting. Similar methodological issues to those described for orexin receptors may also apply, including the possible existence of alternative variants of prepro-orexin or OxA peptides that could complicate detection. Consequently, the question remains unresolved. Should future evidence argue against the presence of OxA in the adrenal gland, alternative peripheral sources contributing to circulating OxA levels in humans would need to be considered.

## 5 Functional role of the orexin system in inflammation regulation

### 5.1 Clinical data

Although narcolepsy itself possesses a well-recognized autoimmune component (Deloumeau et al., 2010; Hartmann et al., 2016; Bonvalet et al., 2017; Gerashchenko and Shiromani, 2004), evidence increasingly suggests that the orexin system is not only affected by immune processes but may also play a modulatory role in immune regulation, particularly through its influence on neuroendocrine pathways.

For example, inhibition of orexinergic neurons has been shown to precipitate inflammation-associated lethargy, highlighting a functional link between orexin signaling and behavioral responses to immune activation (Grossberg et al., 2011). Both animal and human studies have demonstrated reductions in CSF orexin levels during inflammatory states, although the specific isoform measured was not specified (Ogawa et al., 2022; Ogawa et al., 2021). Importantly, these observations are consistent with the hypothesis that the orexin system interacts with the HPA axis to modulate responses to inflammatory and immune stress. Nonetheless, conflicting findings have been reported; for instance, one study found no correlation between CSF OxA concentrations and clinical severity indices, such as APACHE II scores and C-reactive protein levels, in patients with systemic inflammatory response syndrome (SIRS) (Akaishi et al., 2022), indicating that the precise dynamics between orexin signaling, neuroendocrine regulation, and immune function remain to be fully elucidated.

Few studies have suggested possible link to orexin system in autoimmune diseases (Couvineau et al., 2021; Messal et al., 2018; Kucukali et al., 2014; Moharami et al., 2022; Sandikci et al., 2024; Saruhan et al., 2022; Turkoglu et al., 2020; Suzuki et al., 2018; Kanbayashi et al., 2009; Scott et al., 2020). Notably, a higher prevalence of idiopathic intestinal inflammation has been reported among patients with narcolepsy (Couvineau et al., 2021), patients with inflammatory bowel disease experienced more severe narcoleptic symptoms compared to healthy controls (Scott et al., 2020), and increased OX1R expression has been documented in the colonic mucosa of individuals with ulcerative colitis (Messal et al., 2018). Several studies have also demonstrated reduced serum or CSF OxA concentrations in various autoimmune disorders. The mean OxA serum levels were significantly lower in multiple sclerosis patients compared to controls (0.41 ng/mL vs. 0.69 ng/mL; p < 0001) (Moharami et al., 2022), and lower serum OxA levels compared to healthy controls were also found in 41 patients with primary Sjögren’s syndrome (Sandikci et al., 2024). Among multiple sclerosis patients, those presenting with optic neuritis as the initial clinical manifestation exhibited markedly lower serum OxA levels than those without optic neuritis (0.88 ng/mL vs. 1.26 ng/mL; p < 0.05) (Turkoglu et al., 2020). One Japanese case series reported seven neuromyelitis optica (NMO) and multiple sclerosis patients with concomitant narcolepsy having all reduced OxA levels in CSF (Kanbayashi et al., 2009). However, findings are not uniformly consistent; a case-control study reported higher CSF OxA concentrations in patients with relapsing–remitting multiple sclerosis when compared to healthy controls, with no corresponding differences in serum levels (Saruhan et al., 2022). Reduced serum OxA concentrations have also been documented in autoimmune encephalitis with sleep-related symptoms, as well as in NMO patients, compared to healthy controls but also narcolepsy patients (Kucukali et al., 2014). Notably, within the NMO cohort, only those with antibodies against neuronal surface or synaptic antigens (such as N-Methyl-D-Aspartate Receptor and Leucine-Rich, Glioma Inactivated 1) exhibited significantly lower serum OxA levels. Furthermore, a case report described four systemic lupus erythematosus patients with central nervous system involvement and excessive daytime sleepiness, in whom CSF OxA concentrations were found to be reduced during the acute phase in two cases with subsequent normalization paralleling clinical improvement. One patient had lower CSF level in acute phase while OxA level was not measured after clinical improvement, and another maintained normal levels throughout (Suzuki et al., 2018).

In patients with epilepsy, a negative correlation between OxA concentrations and pro-inflammatory cytokines TNF, IL-6 and IL-1β was reported (Li et al., 2023).

Regarding current approved or investigated therapies none of them reports immunological side effects (Mesens et al., 2025a; Dauvilliers et al., 2023; Dauvilliers et al., 2025; Karppa et al., 2020; Kishi et al., 2025; Mignot et al., 2022; Herring et al., 2017; McElroy et al., 2023; Schmidt et al., 2025; Mesens et al., 2025b; van Lemmen et al., 2025). This is true for current DORA approved therapy for insomnia (Karppa et al., 2020; Kishi et al., 2025; Mignot et al., 2022; Herring et al., 2017), newly investigated selective OX1R antagonist like nivasorexant (ACT-539313) and JNJ-61393215 (McElroy et al., 2023; Schmidt et al., 2025), selective OX2R antagonist seltorexant (Mesens et al., 2025a; Mesens et al., 2025b) or selective OX2R agonists like TAK 925 (danavorexton), TAK 994 (firazorexton) and TAK-861 (oveporexton) (Dauvilliers et al., 2023; Dauvilliers et al., 2025; van Lemmen et al., 2025). Although orally available TAK 994 (firazorexton) was discontinued in phase II due to drug-induced liver injury concerns (Dauvilliers et al., 2023).

In summary, direct evidence from clinical studies supporting the involvement of the orexin system in human inflammation remains limited. The majority of available studies are constrained by small sample sizes and rely primarily on correlations between OxA serum or plasma concentrations and disease markers or clinical status. As illustrated by the findings discussed above, caution is warranted when interpreting orexin concentrations in blood, given the potential for methodological variability and confounding factors.

### 5.2 Preclinical data

#### 5.2.1 In vitro

Current evidence for the expression of OX1R and OX2R in immune cells remains limited. One study documented the presence of both receptors in the thymus, spleen, and cervical and axillary lymph nodes of mice, as well as on CD4^+^ and CD8^+^ T lymphocytes and CD11^+^ myeloid cells (Becquet et al., 2019). Additional research has identified OX1R expression in total immune cells isolated from the lamina propria in a dextran sulfate sodium (DSS) colitis mouse model, as well as in human Mono-Mac-6 and HT-29 immune cell lines (Messal et al., 2018). In humans, both OX1R and OX2R have been detected in one study on isolated monocytes (Sun et al., 2018) while one study claim to find OX1R on mononuclear cells of ulcerative colitis patients in colon tissue samples (Messal et al., 2018). However, to date, there is no evidence supporting expression of prepro-orexin or direct secretion of OxA or OxB from immune cells.

Regarding the direct immunomodulatory effect of orexins, previous work has shown the inhibition of inflammatory cytokine secretion (TNF, IL-6, MCP-1) after T cell isolates from lamini propria in DSS-induced colitis mice were incubated with OxA, while INF-γ and IL-12p70 were not significantly affected, and an insignificant reduction was observed for IL-10 (Messal et al., 2018). PBMC isolated from DSS mice and treated with lipopolysaccharide (LPS) plus 1 μM OxA produced less TNF and IL-6 compared to the control without OxA (Messal et al., 2018). Mechanistic investigations in Mono-Mac-6 and HT-29 cell lines further revealed that OxA attenuated IL-8 production by modulating NF-κB signaling, and that this anti-inflammatory effect, especially the inhibition of TNF-induced NF-κB activity in HT-29 cells, could be abolished by a selective phospholipase C inhibitor. Based on these results the authors assumed that the inhibitory effect of OxA on NF-κB activation is mediated through the phospholipase C → Ca^2+^ signalling pathway.

In an oxygen-glucose deprivation/reperfusion model in U251 astroglioma cells OX1R expression was markedly increased from baseline, which was reported to be minimal in untreated cells. Treatment with OxA reversed the upregulation of pro-inflammatory mediators, including IL-1β, TNF, IL-6, and inducible nitric oxide synthase (iNOS), primarily *via* the MAPK and NF-κB p65 signaling pathways (Xu et al., 2021). Co-treatment with SORA1 (SB-334867) abolished these anti-inflammatory effects of OxA. In a co-culture model of Caco-2 cells and primary murine cortical microglia, 30 min pretreatment with 0.2 μM OxA prior to LPS stimulation reduced ROS production and accumulation in microglia (Tunisi et al., 2019). This effect was abolished by co-treatment with the 10 μM SORA1 SB334867 aded 15 min before OxA. In LPS treated microglial BV2 cells pretreatment of cells in 100 nM OxA for 1 h resulted in significant reduction of TNF production (Xiong et al., 2013). In another study using the BV2 microglial cell line, both palmitic acid and LPS increased the expression of OX1R, but not OX2R, whereas pretreatment with 300 nM OxA decreased the expression of IL-6 and iNOS. However, TNF levels in the supernatant were not significantly altered compared with vehicle-treated cells (Duffy et al., 2015).

OX1R expression, but not OX2R, was also found in human fibroblast-like synoviocytes (Sun et al., 2018). Interestingly, the expression was lower in rheumatoid arthritis patients compared to healthy controls. In these cells, OxA reduced the expression of IL-1β, IL-6, IL-8, MMP3 and MMP13, reduced generation of reactive oxygen species and suppressed NF-κB signalling pathway compared to vehicle treated cells.

Treatment with OxB in LPS-stimulated mouse lung microvascular endothelial cells led to reduced markers of barrier damage, evidenced by an increase in transepithelial/transendothelial electrical resistance, upregulation of the Zona Occludens-1 gene, and downregulation of ROCK2, the key gene involved in inflammatory signalling in acute lung injury (Wang et al., 2024a). Protection of tight junction barrier integrity following LPS exposure was also observed in experiments using Caco-2 cells, where 30 min pretreatment with 0.2 μM OxA prior to LPS stimulation attenuated tight junction disruption (Tunisi et al., 2019). Co-treatment with the 10 μM SORA1 SB334867 added 15 min before OxA, abolished this protective effect.

OxA also mitigated oxidative stress in an *in vitro* model of hepatitis B in human hepatocytes, alongside protection of mitochondria from collapse and reduction of the inflammatory cytokines IL-8, TNF, and CXCL2 (Wang et al., 2019). Consistently, OxA attenuated oxidative stress in an *in vitro* model of inflammation in human endothelial cells with reduced production of IL-1β and IL-18 (Zhang et al., 2019). Additional studies have supported OxA’s ability to reduce oxidative stress in various neuronal cell models (Duffy et al., 2016; Sokolowska et al., 2014; Butterick et al., 2012).

#### 5.2.2 In vivo

##### 5.2.2.1 Ischemia-reperfusion injury

The application of orexins in experimental models of inflammation was first reported in 2008 by Bulbul et al., who investigated the gastroprotective effects of OxA in a rat model of ischemia–reperfusion injury (Bulbul et al., 2008). Gastric injury was induced by clamping the celiac artery for 30 min, followed by intravenous infusion of OxA at 1.8 μg/kg/min during the 60-min reperfusion period. OxA administration led to a significant reduction in gastric lesion index, evidenced by fewer erosions in the gastric mucosa. Additionally, treatment decreased markers of oxidative stress, exemplified by lower levels of thiobarbituric acid–reactive substances (an index of lipid peroxidation), as well as reduced gastric myeloperoxidase activity, indicative of diminished neutrophil activation. Notably, OxA did not affect gastric mucus content or mucosal prostaglandin E2 concentrations compared to vehicle infused group.

##### 5.2.2.2 Sepsis

Ogawa et al. (2016) have shown that both centrally and peripherally administered OxA improved survival of mice in septic shock. Treatment with OxA led to decreased production of pro-inflammatory cytokines IL-17, IFN-γ, IL-6 and TNF and increased circulating levels of corticosterone and catecholamines, while no activation of macrophages by OxA was noted. Peripherally administered OxA passed through BBB under septic conditions (Ogawa et al., 2016). These positive effects of OxA on survival in the septic shock model have been confirmed by additional independent studies (Takekawa et al., 2019; Igarashi et al., 2020).

In a mouse model of sepsis-associated lung injury, a marked reduction of serum OxB and pulmonary expression of OX2R was observed, while serum OxA concentrations and pulmonary expression of OX1R remained unchanged compared with healthy control group (Wang et al., 2024a). A single administration of OxB (dose was not specified) improved 7-day survival and reduced Modified Mouse Clinical Assessment Score for Sepsis compared to vehicle treated controls. In addition, OxB ameliorated histopathologic scores of lung injury, reduced serum and bronchoalveolar lavage fluid concentrations of IL6, TNF and CXCL15, and decreased total cell and neutrophil content in bronchoalveolar lavage fluid compared to vehicle treated group. Additionally, in a cecal ligation and puncture model of murine sepsis, intracerebroventricular administration of 11 µg OxA restored temperature, heart rate, and respiratory rate to baseline values, in contrast to saline-treated animals in which these parameters remained altered (Deutschman et al., 2013). Administration of 40 μg/kg i.p OxA 1 h before LPS exposure protected against LPS-induced disruption of intestinal tight junction barrier integrity in mice, as evidenced by preserved occludin immunoreactivity in epithelial cells (Tunisi et al., 2019). This protective effect was abolished by pretreatment with the 30 mg/kg i.p. SORA1 SB334867 administered 30 min before OxA.

Interestingly, another study demonstrated that pretreatment of mice with daridorexant (100 mg/kg daily for 3 days) exerted beneficial effects on i.p. LPS-induced inflammation in both the brain and lungs (Horiuchi et al., 2026). In particular, daridorexant treatment reduced hypothalamic expression of several pro-inflammatory genes, including Cxcl1, Icam1, Ccl2, Ccl7, Tnfaip3, and Tnfaip2, compared with the vehicle-treated group. Similarly, in the lungs, daridorexant decreased the expression of multiple pro-inflammatory genes, such as Cxcl1, Ccl2, Ccl7, and Tnf, which was consistent with reduced protein levels of cytokines and chemokines including CXCL1, CXCL10, CXCL13, G-CSF, and TIMP-1.

##### 5.2.2.3 Neuroinflammation

The anti-inflammatory effects of OxA have also been established in models of neural tissue inflammation. Several studies have shown attenuation of ischemia-induced neuroinflammation in rodent brain (Xu et al., 2021; Li et al., 2020; Xiong et al., 2013; Kitamura et al., 2010; Yuan et al., 2011; Zhu M. et al., 2024; Zhu et al., 2025; Modi et al., 2017). Xiong et al. (2013) showed in the middle cerebral artery occlusion model (MCAO) that both endogenous and exogenously administered OxA provided protection against post-ischemic brain injury, as evidenced by reduced serum TNF levels in OxA-pretreated mice and enhanced post-ischemic upregulation of OX1R on microglial cells (Xiong et al., 2013). This finding is consistent with earlier reports documenting increased OX1R expression on glial cells (Irving et al., 2002) and within the ischemic cortex (Zhu M. et al., 2024) following ischemic injury. Xu et al. (2021) reported the anti-inflammatory effects of OxA in MCAO model mediated *via* NF-κB inhibition and MAPK signalling pathways. OxA treatment attenuated the increase of serum pro-inflammatory factors (IL-1β, TNF, IL-6 and iNOS), decreased translocation of p65 (NF-κB signalling pathway) and downregulated the phosphorylation of ERK and p38 MAPK compared to vehicle treated MCAO rats. Based on their *in vitro* data OX1R was proposed to mediate these effects. In contrast, another paper reported decreased OX1R mRNA and protein level expression in the whole hypothalamus in the rat model of ischemic stroke at high altitude (4,000 m above sea level), induced by the MCAO combined with hypobaric hypoxia, relative to sham operated control (Zhu et al., 2025). In this model, administration of OxA (100 μg/kg) immediately after arterial occlusion led to improvements in microcirculatory blood flow and velocity, enhanced microcirculation oxygen saturation, lower neurological deficit scores, reduced cerebral infarct size, decreased oxidative stress as indicated by increased superoxide dismutase activity and decreased malondialdehyde (a marker of lipid peroxidation) in the cortex and lower serum IL-6 and TNF levels compared to vehicle-treated controls. In another work with rat MCAO model OxA (30 μg/kg) intracerebroventricular administration resulted in increased gene expression of endothelial nitric oxide synthase (eNOS), which is considered beneficial in ischemic injury (Chen et al., 2017; Greco et al., 2018). Levels of neuronal and inducible nitric oxide synthase (nNOS and iNOS, respectively), typically associated with deleterious effects (Chen et al., 2017; Greco et al., 2018), were unchanged relative to vehicle-treated animals (Zhu M. et al., 2024). Interestingly, in transgenic rats overexpressing OxA, both eNOS and iNOS gene expression were upregulated, while nNOS remained similar to vehicle controls. In a rat model of neuropathic pain induced by chronic constriction injury (CCI) of the sciatic nerve, OX2R, but not OX1R, expression was reduced in spinal cord tissue, while intraperitoneal administration of OxB (0.1 and 0.2 μM) attenuated inflammation, as evidenced by decreased gene and protein expression of IL-6, TNF, COX-2, and iNOS, reduced NO release and PGE2 production, suppression of JNK phosphorylation and p65 (NF-κB subunit) protein levels, as well as reduced microglial activation compared with the vehicle treated group (Zhu Z. et al., 2024). Furthermore, in rat model of cardiac arrest followed by cardiopulmonary resuscitation, intranasal administration of 1.8 µg OxA administered 30 min after the return of spontaneous circulation reduced mRNA expression of IL-1β, TNF, iNOS and CD11b in the hippocampus, IL-1β, iNOS in the hypothalamus and IL-1β in the striatum (Modi et al., 2017). In a hypobaric hypoxia model, Hcrt knock-in (prepro-orexin knock-in) transgenic rats exhibited reduced microglial activation, restoration of PI3K/AKT/mTOR pathway gene expression, and decreased expression of both IL-1β and IL-6 at the gene and protein levels in the cortex (Zhu et al., 2026).

In a mouse model of sepsis-associated encephalopathy (SAE), intranasal administration of OxA at doses starting from 50 μg/kg to 500 μg/kg attenuated SAE manifestations, including reduced mortality, mitigated cognitive and emotional deficits, decreased cerebral oedema, preserved BBB integrity, and reduced ultrastructural brain damage (Guo et al., 2024b). Mechanistically, OxA treatment suppressed the expression of the IL-1β and TNF, inhibited microglial activation, downregulated the expression of the Rras and RAS proteins, and reduced the phosphorylation of P-38 and c-Jun N-terminal kinase. Interestingly, the protective effects of OxA were not reversed by SORA1, whereas SORA2 antagonized these effects. In another SAE model study by the same author, intranasal single administration of OxA (250 μg/kg) improved cognitive function, decreased neuronal apoptosis, reduced serum inflammatory markers (TNF, IL‐1β), and reduced oxidative stress as well as microglial and astrocytic activation within the hippocampus compared to vehicle treated group (Guo et al., 2024c). In a recent study, injection of prepro-orexin antisense lentiviral vector into lateral hypothalamus prior to i.p. administration of LPS resulted in significant reduction of IL1α and IL1β in lateral hypothalamus but did not affect levels of plasma TNF and IL6 (Frick et al., 2025). In seasonal affective disorder (SAD) model in rats i.c.v. administration of 17.8 µg OxA resulted in higher expression of anti-inflammatory cytokines IL-4 and IL-10, lower expression of IL-6 and CD11b, reduced infiltration of astrocytes and microglia in the cortex, and a reduced complexity of microglia (Costello et al., 2026). The effect of OxA on neuroinflammation has also been investigated in a model of experimental autoimmune encephalitis (EAE) (Fatemi et al., 2016; Becquet et al., 2019). OxA improved the course of EAE, decreased the expression of the inflammatory parameters IL-12, MMP-9, and iNOS, and increased the expression of the anti-inflammatory parameters TGF-β1 and myelin basic protein. The administration of SORA1 SB-334867 exacerbated the disease severity (Fatemi et al., 2016). Becquet et al. (2019) demonstrated an improvement in the course of EAE even after peripheral administration of OxA (i.p.), accompanied by decreased expression of the chemokines CCL2 (MCP-1) and CXCL10 (IP-10) and cytokines IFN-γ, IL-17, TNF, IL-10 and, unexpectedly, the anti-inflammatory TGF-β (Becquet et al., 2019).

A summary of neuroinflammation-related animal model studies is provided in Table 9.

**TABLE 9:** Overview of preclinical studies investigating the anti-inflammatory effects of orexin A and orexin signaling in models of neuroinflammation and brain injury.

Publication	Model	Species	Dose of OxA	Main findings
Irving et al., 2002	MCAO	Mice	X	Increased OX1R expression, but not OX2R, in microglia within the brain.
Xiong et al., 2013	MCAO	Mice	7.1 µg i.c.v.	Reduced macrophage/microglial infiltration and decreased expression of inflammatory markers in the brain.
Xu et al., 2021	MCAO	Rat	30 μg/kg i.c.v.	Reduced pro-inflammatory marker expression and astrocyte activation, accompanied by inhibition of the NF-κB and MAPK signaling pathways.
Zhu et al., 2025	MCAO + hypobaric hypoxia	Rat	100 μg/kg i.p.	Reduced serum IL-6 and TNF levels.
Zhu et al., 2026	Hypobaric hypoxia	Rat	Hcrt (prepro-orexin) knock-in transgenic rat	Reduced microglial activation, restored PI3K/AKT/mTOR pathway gene expression, and decreased IL-1β and IL-6 expression at both the mRNA and protein levels in the cortex.
Zhu et al., 2024b	CCI of sciatic nerve	Rat	300–600 ug i.p. (OxB)	OX2R, but not OX1R, expression was reduced in spinal cord tissue. Reduced microglial activation, reduced IL-6, TNF, COX-2, iNOS, NO, PGE2, p-JNK, and p65 levels.
Modi et al., 2017	Asphyxial-cardiac arrest	Rat	1.8 µg i.n.	Reduced mRNA expression of IL-1β, TNF, iNOS, and CD11b in the hippocampus; IL-1β and iNOS in the hypothalamus; and IL-1β in the striatum.
Guo et al., 2024b	CLP‐induced SAE	Mice	50–500 μg/kg i.n.	Reduced expression of pro-inflammatory markers in the brain and inhibited microglial activation. Downregulated Rras and RAS protein expression and reduced phosphorylation of p38 and JNK.
Guo et al., 2024c	CLP‐induced SAE	Mice	250 μg/kg i.n.	Reduced microglial and astrocyte activation, inhibited P-ERK and NF-κB signaling, and decreased IL-1β and TNF-α release.
Tunisi et al., 2019	i.P. LPS induced systemic inflammation	Mice	40 μg/kg i.p	Reduced microglial activation.
Frick et al., 2025	i.P. LPS induced systemic inflammation	Rat	Prepro-orexin antisense lentiviral vector injection into lateral hypothalamus	No significant effect on plasma TNF or IL-6 levels, but significantly reduced IL-1α and IL-1β levels in the lateral hypothalamus.
Costello et al., 2026	SAD	Rat	17.8 µg i.c.v.	Increased IL-4 and IL-10 expression, decreased IL-6 and CD11b expression, reduced astrocyte and microglial infiltration in the cortex, and decreased microglial complexity.
Fatemi et al., 2016	EAE	Mice	1.1 and 106.9 µg i.c.v.	Reduced clinical scores and decreased the expression of iNOS, MMP9, and IL-12, while increasing TGF-β and myelin basic protein (MBP) expression in spinal cord. Treatment with SORA1 produced opposite effects.
Becquet et al., 2019	EAE	Mice	100–300 μg i.p./r.o.	Reduced pathogenic CD4^+^ T-lymphocyte infiltration, reduced CNS expression of chemokines and cytokines, reduced demyelination, astrogliosis, and microglial activation.

CCI, chronic constriction injury; CLP, cecal ligation and puncture; i.c.v., intracerebroventricular; IL, interleukin; i.n., intranasal; i.p., intraperitoneal; MAPK, mitogen-activated protein kinase; MCAO, middle cerebral artery occlusion; OX1R, orexin one receptor; OX2R, orexin two receptor; OxA, orexin A; SAD, sleep apnea disorder; SAE, sepsis-associated encephalopathy; r.o., retroorbital.

Conversely, in a rat high-fat diet model, daily i.p. administration of SORA1 (SB334867 5 mg/kg/day) for 12 weeks resulted in decreased number of microglial cells in the hippocampus (Dong et al., 2024). Similarly, in another study, treatment with lemborexant (30 mg/kg daily) in a mouse model of tauopathy resulted in a significant reduction of reactive microgliosis and decreased disease-associated inflammatory microglial phenotype linked to tau pathology (Parhizkar et al., 2025). This is in contrast to multiple studies reporting that OxA or OxB administration reduces microglial activation (Guo et al., 2024b; Becquet et al., 2019; Tunisi et al., 2019; Xiong et al., 2013; Duffy et al., 2015; Zhu Z. et al., 2024; Zhu et al., 2026; Guo et al., 2024c; Costello et al., 2026; Synchikova and Korneva, 2023; An et al., 2017; Duffy et al., 2019). Some studies have even reported complete abolition of the OxA-induced effect following co-treatment with the SORA1 (Tunisi et al., 2019). We have already mentioned anti-inflammatory effect of daridorexant after i.p. administration of LPS in mice (Horiuchi et al., 2026) and interestingly there are some other studies supporting the beneficial effects of orexin antagonists in neurodegenerative diseases (Raich et al., 2022; Hada et al., 2026; Lucey et al., 2023).

##### 5.2.2.4 Inflammatory bowel disease

*In vivo* studies have demonstrated that long-term i.p. administration of OxA significantly ameliorates inflammation in a murine DSS colitis model, as indicated by reduced body weight loss and lower disease activity index scores compared to controls (Messal et al., 2018). OxA treatment was associated with decreased expression of pro-inflammatory cytokines (TNF, IL-6, MCP-1), an effect also observed in colitis models involving IL-10 and NOX-1 knockout mice, where reductions in IFN-γ, IL-17, IL-1α, and IL-1β were additionally documented. OX1R is strongly implicated as the primary receptor for these effects, as they were not observed when SORA1 SB-408124 was co-administered in both models and were absent in DSS OX1R −/− KO mice. A recent study compared the efficacy of infliximab (7 μg/kg) and OxA (1 μmol/kg) in a murine DSS colitis model (Blais et al., 2023). Both treatments significantly attenuated inflammatory markers in blood compared to control group, namely, restored MPO levels and reduced LPS-binding protein (LBP), TNF and IL-6 levels. Similar findings were obtained from colon samples where both treatments reduced the expression of TNF, IL-6 and IL-10 and restored expression of tight-junction marker occludin and the mucin-secreting cell markers Muc2 and Klf4 compared with the controls. Histological analysis revealed reduced immune cell infiltration and decreased DSS-induced epithelial hyperproliferation in both treatment groups relative to control.

##### 5.2.2.5 Other models

In the adjuvant-induced arthritis rat model, 8 days of i.v. OxA (30 mg/kg) treatment led to significant reduction in pain sensitivity, nerve growth factor, a key mediator of inflammatory and chronic pain, alongside increased body weight and elevated neuropeptide Y compared to vehicle treated group (Mohamed and El-Hadidy, 2014). In another study, administration of SORA1 (SB-334867, 10 mg/kg) resulted in decreased phagocytic activity, as measured by both the percentage of phagocytosing peritoneal macrophages and the number of particles engulfed, relative to untreated animals (Izgut-Uysal et al., 2011). With respect to the gastroprotective actions of OxA, two studies in rat gastric ulcer models demonstrated increased superoxide dismutase mRNA expression following OxA treatment (Szlachcic et al., 2013), and showed that the protective effects of OxA were abrogated by OX1R antagonism, cyclooxygenase (COX-1/COX-2) inhibition, or nitric oxide synthase inhibition (Brzozowski et al., 2008).

#### 5.2.3 Summary of preclinical data

It may be speculated that different disease models involve distinct mechanisms underlying the anti-inflammatory effects of orexins. Although central mechanisms may plausibly contribute across all models, some evidence suggests that these effects can be restricted to the CNS. In a recent study injection of a prepro-orexin antisense lentiviral vector into the lateral hypothalamus prior to intraperitoneal LPS administration significantly reduced inflammatory markers in the lateral hypothalamus but did not affect inflammatory markers in plasma (Weber et al., 2024). Moreover, substantial evidence demonstrates direct effects of orexins on immune-related cells supporting the plausibility of direct immunomodulatory actions. The situation is further complicated by the fact that the commonly used OxA is capable of crossing the BBB (Kastin and Akerstrom, 1999), meaning that even peripherally administered OxA may exert its effects through central mechanisms. Conversely, intracerebroventricular (i.c.v.) or intranasal (i.n.) administration of OxA should theoretically restrict its action primarily to the CNS, however, these routes of administration are predominantly used in studies investigating neuroinflammatory models where peripheral inflammation is not usually evaluated. In few studies employing peripherally administered orexins in neuroinflammatory models (Becquet et al., 2019; Tunisi et al., 2019; Zhu et al., 2025; Zhu Z. et al., 2024), peripheral mechanisms may have also contributed to the observed effects such as involvement of peripheral corticosteroids regulated by the orexin system, particularly in sepsis-associated encephalopathy, where orexin administration reduced brain edema (Guo et al., 2024b) similarly to corticosteroid treatment (Blais et al., 2023). The direct effects on microglia are well documented (Guo et al., 2024b; Becquet et al., 2019; Tunisi et al., 2019; Xiong et al., 2013; Duffy et al., 2015; Zhu Z. et al., 2024; Zhu et al., 2026; Guo et al., 2024c; Costello et al., 2026; Synchikova and Korneva, 2023; An et al., 2017; Duffy et al., 2019) and in theory may be mediated by orexins originating from both central and peripheral sources. Lastly, studies in physiological tissue barriers, including the intestinal and pulmonary, suggest that orexins may support tight junction integrity (Tunisi et al., 2019; Wang et al., 2024a; Blais et al., 2023). If similar mechanisms occur at the BBB, orexins could theoretically reduce immune cell infiltration into the CNS.

In sepsis models observed anti-inflammatory effect could be mediated centrally through orexin-induced activation of the HPA axis, as proposed by Ogawa et al. (2016) (Ogawa et al., 2016), importantly he demonstrated this effect with peripherally administrated OxA thus making plausible that central contribution of HPA axis is actual mediated from peripheral OxA secretion. Because OxA was administrated peripherally a contribution of direct effect on corticosteroid secretion could be also likely as demonstrated in chapter 4. In addition, antioxidative effects, as demonstrated in ischemia–reperfusion injury models, are also plausible explanation (Bulbul et al., 2008).

Finally, in IBD models multiple mechanisms are also likely to contribute. Although direct effects on immune cells have been demonstrated in at least some of these models (Messal et al., 2018), orexin-mediated regulation *via* the enteric nervous system and the gastrointestinal endocrine system is also a plausible mechanism (de Jonge, 2013), given the previously discussed evidence for the presence of the orexin system and potential OxA production in the gastrointestinal tract, particularly within ENS and endocrine cells (Messal et al., 2018; Ehrstrom et al., 2005a).

There is also some evidence suggesting beneficial effects of orexin receptor antagonists, including the DORA daridorexant (Horiuchi et al., 2026), lemborexant (Parhizkar et al., 2025) and the SORA1 compounds (Dong et al., 2024; Izgut-Uysal et al., 2011). However, as these studies remain limited in number and highly heterogeneous with respect to methodology, compounds used, and disease models, the overall significance of these findings remains difficult to interpret. In theory, these paradoxical effects could be explained by distinct CNS-mediated mechanisms of orexin signaling, receptor signaling bias (Gratio et al., 2025), or the use of high doses of the compounds (Horiuchi et al., 2026). Multiple studies reporting beneficial effects of orexin receptor antagonists have (Parhizkar et al., 2025) demonstrated reduced activation of phagocytic cells (microglia/macrophages) (Dong et al., 2024; Izgut-Uysal et al., 2011). Therefore, it is possible that this signaling pathway is mediated through a mechanism commonly activated by both agonists (OxA) and antagonists *via* Gβγ signaling, as previously reported (Gratio et al., 2025).

Summary of all preclinical studies supporting either OX1R or OX2R as transducer of orexins anti-inflammatory effects is in Table 10.

**TABLE 10:** Comparison of studies supporting either OX1R or OX2R as transducers of orexins anti-inflammatory effect.

Publication	Disease model	Species	Suggested receptor responsible for the effect	Findings
Irving et al., 2002	MCAO	Rat	OX1R	Increased OX1R mRNA and protein expression following MCAO while OX2R mRNA and protein expression did not change.
İzgüt-Uysal et al., 2011	Starved rat	Rat	OX1R	SORA1 SB-334867 caused decrease phagoctyosis and number of phagocytosed particles.
Fatemi et al., 2016	EAE	Mouse	OX1R	SORA1 (SB-334867) increased inflammatory markers in EAE.
Messal et al., 2018	DSS induced colitis IL-10/NOX1^dKO^ colitis	Mouse	OX1R	Diminished OxA anti-inflammatory effect when SORA1 SB-408124 was co-administered in both models (DSS and IL-10/NOX1^dKO^) and in DSS model in OX1R −/− KO mice.
Li et al., 2020	ICH	Mouse	OX2R	SORA1 (SB-334867) did not reversed OxA anti-inflammatory effect while SORA2 (JNJ-10397049) did.
Guo et al., 2024b	CLP (SAE)	Mouse	OX2R	SORA1 (SB-334867) did not reversed OxA anti-inflammatory effect while SORA2 (JNJ-10397049) did.
Wang et al., 2024b	CLP (ALI)	Mouse	OX2R	Greatly reduced expression of OX2R in pulmonary tissue and reduced serum OxB levels. Single administration of OxB increased survival and reduced markers of ALI.
Zhu et al., 2024b	CCI of sciatic nerve	Rat	OX2R	OX2R, but not OX1R, expression was reduced in the spinal cord tissue after injury.

ALI, Acute lung injury; CCI, chronic constriction injury; CLP, cecal ligation and puncture sepsis model; DSS induced colitis, dextran sulphate sodium induced colitis; IL-10/NOX1dKO, spontaneous model of colitis; EAE, experimental autoimmune encephalomyelitis; ICH, intracerebral haemorrhage; MCAO, middle cerebral artery occlusion; SAE, sepsis associated encephalopathy; OX1R, orexin one receptor; OX2R, orexin two receptor; OxA, orexin A.

## 6 Discussion

### 6.1 Summary of current findings

The available evidence suggests that the orexin system may influence inflammatory processes through multiple mechanisms. Central mechanisms through neuroimmune regulation likely play an important role, particularly in neuroinflammatory models, where their contribution appears indisputable, as most studies use intracerebroventricular (i.c.v.) or intranasal (i.n.) administration, which limits the systemic exposure.

Although the science of neuroimmune interactions is still a developing field, two major mechanisms of central neuroimmune regulation have been proposed. The first involves neuroendocrine regulation, particularly activation of the HPA axis, which has been relatively well described in relation to the orexin system (Inutsuka and Yamanaka, 2013; Zhou et al., 2026; Date et al., 1999). This pathway may be activated directly by circulating cytokines reaching the paraventricular nucleus (PVN) of the hypothalamus (Zhou et al., 2026; Silverman et al., 2005), a region receiving dense projections from orexin neurons originating in the lateral hypothalamus (Dergacheva et al., 2017), or indirectly by the second neuroimmune mechanism involving autonomic regulation through the cholinergic anti-inflammatory pathway sending projections from nucleus tractus solitarius (NTS) to PVN (Olofsson et al., 2012; Martelli et al., 2014). As the primary brainstem hub for vagal afferent signaling involved in neuroimmune regulation, the NTS also appears to be influenced by the orexin system, as both orexin receptors are expressed in the NTS and NTS neurons have been shown to respond to OxA (Olofsson et al., 2012; Ohi et al., 2022; Yang and Ferguson, 2003; Yang et al., 2003; Wang et al., 2024b; Ben et al., 2026; Wang et al., 2021). Similar orexinergic projections are present in the dorsal motor nucleus of the vagus (DMV), the major efferent nucleus associated with the vagal cholinergic anti-inflammatory pathway (Bigalke et al., 2022; Pavlov et al., 2003). Autonomic neuroimmune regulation also involves sympathetic projections to immune organs such as the spleen, bone marrow, and lymph nodes (Schiller et al., 2021). These sympathetic pathways are known to modulate immune responses (Schiller et al., 2021; Bucsek et al., 2018; Pongratz and Straub, 2014), and orexin neurons from the lateral hypothalamus project to key sympathetic regulatory centres, including the rostral ventrolateral medulla (RVLM) (Geerling et al., 2003; Li and Nattie, 2014), where they modulate sympathetic nervous system activity in several physiological systems (Bigalke et al., 2022; Li and Nattie, 2014). While the parasympathetic nervous system is generally considered to exert predominantly anti-inflammatory effects, sympathetic regulation appears to be more context-dependent and may produce either anti- or pro-inflammatory outcomes depending on the duration and physiological context of activation (Pongratz and Straub, 2014). Interestingly, the spleen is primarily innervated by sympathetic fibers, it can still be influenced by vagally mediated anti-inflammatory reflexes (Martelli et al., 2014; Huston et al., 2006). It is also possible that additional orexin projections from the lateral hypothalamus, including those to the basal forebrain (Villano et al., 2022), ventral tegmental area (Villano et al., 2022; Wang Y. et al., 2025), amygdala (Wang Y. et al., 2025) or arcuate nucleus (Villano et al., 2022; Wang Y. et al., 2025) may contribute to neuroimmune regulation (Zhou et al., 2026; Schiller et al., 2021; Lehner et al., 2019).

While these mechanisms provide a plausible explanation for observed anti-inflammatory effects in some experimental models, they also raise important pharmacological concerns. Drugs targeting central orexin signaling inevitably affect sleep regulation, arousal, and metabolic homeostasis (Inutsuka and Yamanaka, 2013), which may limit their therapeutic applicability in inflammatory diseases. In contrast, clinical trials of drugs targeting the orexin system have, to date, not reported immune system–related safety concerns or adverse events (Jacobson et al., 2022; Mesens et al., 2025a; Dauvilliers et al., 2023; Dauvilliers et al., 2025; Karppa et al., 2020; Kishi et al., 2025; Mignot et al., 2022; Herring et al., 2017; McElroy et al., 2023; Schmidt et al., 2025; Mesens et al., 2025b; van Lemmen et al., 2025). However, immune-related outcomes have not been a primary focus of these studies, and therefore potential effects on immune function, particularly in patient subpopulations with autoimmune diseases, cannot be excluded. Moreover, currently approved therapies are primarily designed to target central nervous system orexin signaling and are typically characterized by relatively short half-lives (Jacobson et al., 2022; Chino et al., 2025; Uchiyama et al., 2026). As a result, their peripheral pharmacological effects are likely limited.

There are receptors for several other neurotransmitters—including acetylcholine, serotonin, and dopamine expressed on immune cells, which are also capable of synthesizing these signaling molecules themselves (Mashimo et al., 2022; Herr et al., 2017; Wang H. et al., 2025). Regarding the case of the peripheral anti-inflammatory effects of orexin system, although still emerging, available evidence supports the expression of orexin receptors in some immune-related cells (Jacobson et al., 2022; Xiong et al., 2013; Modi et al., 2017; Irving et al., 2002; Chen et al., 2017; Greco et al., 2018; Guo et al., 2024c) and their involvement in the regulation of inflammatory signaling (Messal et al., 2018; Guo et al., 2024b; Xu et al., 2021), as well as the presence of the orexin system in immune-associated tissues (Messal et al., 2018; Jacobson et al., 2022; The Human Protein Atlasa; The Human Protein Atlasb; The Human Protein Atlasc; Sun et al., 2018). Although evidence for prepro-orexin expression in these tissues is limited (The Human Protein Atlasa; The Human Protein Atlasb), a local paracrine role cannot be excluded; however, circulating orexins are likely to play a role. The origin and physiological significance of these circulating orexin peptides remain yet unresolved. While given OxA ability to cross BBB (Dhuria et al., 2009; Kastin and Akerstrom, 1999; Ogawa et al., 2016; Deadwyler et al., 2007; Van de Bittner et al., 2018) it seems possible that some of circulating OxA may derive from the CNS however there is good evidence that actually the majority may originate from peripheral sources (HM et al., 2021). Potential contributors include the gastrointestinal tract (Nakabayashi et al., 2003; Ehrstrom et al., 2005a; Wang et al., 2022; Khan and Ghia, 2010; Populin et al., 2021), bone marrow (Nakabayashi et al., 2003; The Human Protein Atlasa), lymphoid tissues (Nakabayashi et al., 2003; The Human Protein Atlasa), the reproductive tract (Nakabayashi et al., 2003; Ozkanli et al., 2018), Kidney (Lucey et al., 2023; Salvadore et al., 2020) and the adrenal gland (Randeva et al., 2001; Nakabayashi et al., 2003; Karteris et al., 2001). Since the PK profile of OxA appears favorable (Ehrstrom et al., 2005a; Ehrstrom et al., 2004; Dhuria et al., 2009), it raises the possibility that this circulating OxA could modulate immune responses in peripheral tissues. Moreover, given its ability to cross the BBB (Dhuria et al., 2009; Kastin and Akerstrom, 1999; Ogawa et al., 2016; Deadwyler et al., 2007; Van de Bittner et al., 2018), in theory, the peripheral tissues could through this circulating OxA also influence immune regulation within the CNS itself. Such central and peripheral interplay could be demonstrated on the case of HPA axis where the orexin system has been shown to regulate its activity not only *via* central mechanisms (Lawrence, 2017; Kok et al., 2002), but also through direct effects at the level of the adrenal gland (Mazzocchi et al., 2001a; Guo X. et al., 2024; Randeva et al., 2001; Malendowicz et al., 1999; Nanmoku et al., 2002; Ziolkowska et al., 2005; Soejima et al., 2023; Ramanjaneya et al., 2008; Chang et al., 2014; Wenzel et al., 2009; Ramanjaneya et al., 2009).

Physiologically, OxA-mediated immunomodulation is likely dominant over OxB. Firstly, OxA is more stable (Saitoh and Sakurai, 2023), has well-documented exposure with detectable concentrations in systemic circulation (HM et al., 2021), and also readily crosses the BBB and other physiological membranes (Kastin and Akerstrom, 1999). It binds equally to both OX1R and OX2R, whereas OxB preferentially binds to OX2R (Sakurai et al., 1998).

Secondly, studies of the HPA axis consistently show that only higher doses of OxB exerted comparable effects to OxA (2–10 times higher doses needed) (Kuru et al., 2000; Jaszberenyi et al., 2000; Malendowicz et al., 1999; Nowak et al., 2000). It is unclear whether this reflects the lower stability of OxB and thus diminished exposure, the decreased potency of OxB-OX2R complexes compared with OX1R activation, or both. Numerous investigations have identified OX1R as the primary receptor for direct OxA-induced corticosteroid secretion from adrenal glands (Mazzocchi et al., 2001a; Guo X. et al., 2024; Malendowicz et al., 1999; Nanmoku et al., 2002; Ziolkowska et al., 2005; Soejima et al., 2023; Ramanjaneya et al., 2008; Chang et al., 2014; Ramanjaneya et al., 2009).

Thirdly, six *in vitro* studies provide direct evidence pointing towards OX1R as observed anti-inflammatory effect transducer (Messal et al., 2018; Xu et al., 2021; Sun et al., 2018; Tunisi et al., 2019; Xiong et al., 2013; Duffy et al., 2015) although two other studies suggest that OX2R signalling could also contribute (Sun et al., 2018; Sokolowska et al., 2014).

Pre-clinical studies suggest involvement of both OX1R and OX2R on various inflammation related markers and endpoints. Totally four papers point to OX1R (Messal et al., 2018; Fatemi et al., 2016; Irving et al., 2002; Izgut-Uysal et al., 2011; Brzozowski et al., 2008) and four papers point towards OX2R (Guo et al., 2024b; Li et al., 2020; Wang et al., 2024a; Zhu Z. et al., 2024), although OxA have been used in most of the studies. In clinical studies, only OxA concentrations have been investigated and an isolated data correlated OX1R expressions with inflammation (Couvineau et al., 2021). While OxA is generally more potent and more frequently studied there is also emerging evidence that OxB or OX2R activation can contribute to these effects as well as documented particularly in models of neuropathic pain, sepsis, and neuroinflammation (Guo et al., 2024b; Li et al., 2020; Sun et al., 2018; Wang et al., 2024a; Sokolowska et al., 2014; Zhu Z. et al., 2024).

The available evidence is heterogenous with respect to methodology used, therefore species specificity, sex-differences or context-specific orexin functions cannot be ruled out. On top of that existence of new variants of orexin receptors in peripheral tissues is also plausible (Chen and Randeva, 2004; Chen et al., 2006; Chen and Randeva, 2010). If functional, these receptors could allow orexins to directly modulate immune cell activity, cytokine production, or inflammatory signaling pathways. Such mechanisms would significantly increase the pharmacological attractiveness of the orexin system as a therapeutic target, particularly if peripheral actions could be selectively modulated without substantial central nervous system effects.

**FIGURE 6:**
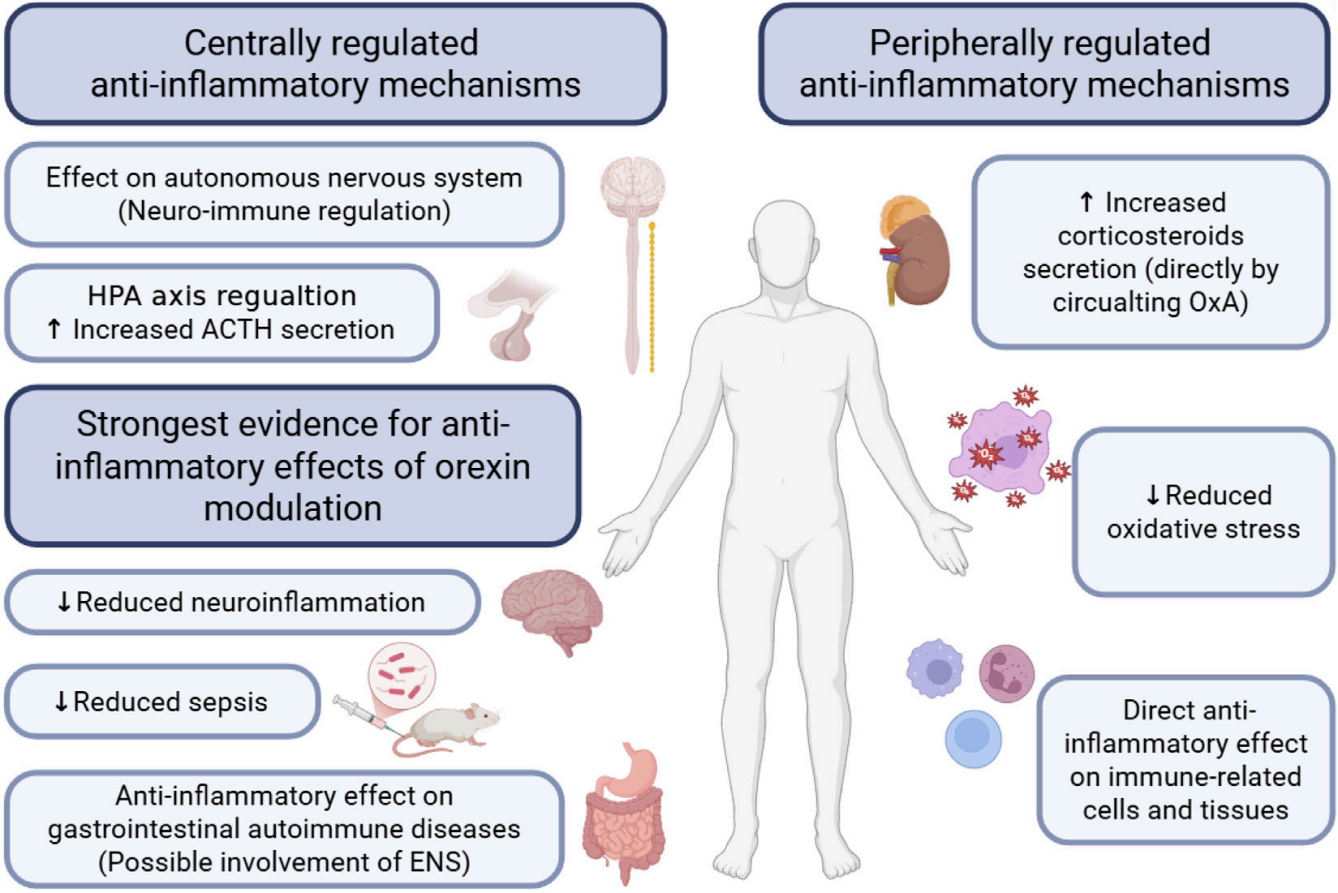
Summary of proposed immunomodulation effects of orexin system. Created in BioRender.com.

Proposed anti-inflammatory effect of orexins are summarized in Figure 6.

### 6.2 Identifying current research gaps and future perspectives

Based on the literature discussed above, several important aspects of orexin-mediated immunomodulation remain insufficiently understood:

- Uncertainty between central vs. peripheral regulation
- Unclear source of circulating orexin
- Uncertainty regarding true orexin peptide blood levels
- Conflicting data regarding receptor distribution in adrenal gland
- Incomplete knowledge of receptor distribution in immune tissues
- Lack of evidence for anti-inflammatory effect from clinical trials
- Lack of immune safety evaluation for current orexin therapeutics for specific subpopulations

Future research should therefore focus on several key areas. First, the distribution and functional relevance of orexin receptors in peripheral immune tissues require further clarification. Second, the existence and regulation of peripheral sources of orexin peptides should be systematically investigated. Third, potential immune-related effects of orexin-targeting therapies should receive greater attention, particularly in patient subpopulations with autoimmune diseases such as inflammatory bowel disease or neuroautoimmune disorders or patients with inflammatory conditions such as sepsis. Finally, the development of pharmacological agents capable of selectively targeting peripheral orexin signaling may represent a promising strategy to utilize the immunomodulatory properties of this system while minimizing central nervous system adverse effects.

## 7 Conclusion

Accumulating evidence indicates that the orexin system is actively involved in the regulation of inflammation although it is important to note that much of the current evidence is derived from *in vitro* experiments and animal models while evidence from human trials remains limited. These actions are believed to occur through several mechanisms both central and peripheral, including modulation of the autonomous nervous system and HPA axis, direct stimulation of corticosteroid secretion from the adrenal cortex, and direct effects on immune cells, particularly microglia in the central nervous system. It seems that the majority of the anti-inflammatory signalling is probably transduced primarily *via* OX1R, although both OX1R and OX2R may contribute depending on tissue context.

From a pharmacological perspective, these findings are increasingly relevant as orexin-targeting therapies, including receptor antagonists and emerging agonists, enter clinical practice. A better understanding of how modulation of the orexin system influences inflammatory pathways may therefore have implications for patients with inflammatory or autoimmune diseases. However, further research is required to clarify underlying mechanisms, validate findings in human systems, and determine whether any observed effects translate into clinically meaningful outcomes. If validated, these insights could open new direction for the development of selective therapeutic strategies targeting inflammation through orexin pathways.

## References

[B1] Abulaiti A. Xu P. R. Duo L. K. (2008). Diagnostic values of serum Orexin-a levels in children with obstructive sleep apnea-hypopnea syndrome. Zhonghua Er Ke Za Zhi 46 (4), 291–296. 10.3760/cma.j.issn.0578-1310.2008.04.113 19099733

[B2] Adam J. A. Menheere P. P. van Dielen F. M. Soeters P. B. Buurman W. A. Greve J. W. (2002). Decreased plasma Orexin-a levels in obese individuals. Int. J. Obes. Relat. Metab. Disord. 26 (2), 274–276. 10.1038/sj.ijo.0801868 11850761

[B3] Akaishi M. Hashiba E. Takekawa D. Kushikata T. Hirota K. (2022). Plasma orexin a does not reflect severity of illness in the intensive care units patients with systemic inflammation. JA Clin. Rep. 8 (1), 7. 10.1186/s40981-022-00498-4 35064847 PMC8783934

[B4] Akbulut G. Gezmen-Karadag M. Ertas Y. Uyar B. B. Yassibas E. Turkozu D. (2014). Plasma Orexin-a and ghrelin levels in patients with chronic obstructive pulmonary disease: interaction with nutritional status and body composition. Exp. Ther. Med. 7 (6), 1617–1624. 10.3892/etm.2014.1611 24926354 PMC4043582

[B5] Akca O. F. Uzun N. Kilinc I. (2020). Orexin a in adolescents with anxiety disorders. Int. J. Psychiatry Clin. Pract. 24 (2), 127–134. 10.1080/13651501.2019.1711425 31913740

[B6] Aksu K. Firat Guven S. Aksu F. Ciftci B. Ulukavak Ciftci T. Aksaray S. (2009). Obstructive sleep apnoea, cigarette smoking and plasma Orexin-a in a sleep clinic cohort. J. Int. Med. Res. 37 (2), 331–340. 10.1177/147323000903700207 19383226

[B7] Al-Barazanji K. A. Wilson S. Baker J. Jessop D. S. Harbuz M. S. (2001). Central Orexin-a activates hypothalamic-pituitary-adrenal axis and stimulates hypothalamic corticotropin releasing factor and arginine vasopressin neurones in conscious rats. J. Neuroendocrinol. 13 (5), 421–424. 10.1046/j.1365-2826.2001.00655.x 11328451

[B8] Alain C. Pascal N. Valerie G. Thierry V. (2021). Orexins/hypocretins and cancer: a neuropeptide as emerging target. Molecules 26 (16), 4849. 10.3390/molecules26164849 34443437 PMC8398691

[B9] Alexandre D. Hautot C. Mehio M. Jeandel L. Courel M. Voisin T. (2014). The orexin type 1 receptor is overexpressed in advanced prostate cancer with a neuroendocrine differentiation, and mediates apoptosis. Eur. J. Cancer 50 (12), 2126–2133. 10.1016/j.ejca.2014.05.008 24910418

[B10] Alkan A. A. Arslan B. Ozcan D. Tekin K. (2025). Serum neopterin and Orexin-a levels in different stages of diabetic retinopathy. Clin. Exp. Optom. 108 (5), 585–591. 10.1080/08164622.2024.2374875 39009974

[B11] Almasi N. Zengin H. Y. Koc N. Ucakturk S. A. Iskender Mazman D. Heidarzadeh Rad N. (2022). Leptin, ghrelin, Nesfatin-1, and Orexin-a plasma levels in girls with premature thelarche. J. Endocrinol. Invest 45 (11), 2097–2103. 10.1007/s40618-022-01841-3 35764868

[B12] Alvez M. B. Bergstrom S. Kenrick J. Johansson E. Aberg M. Akyildiz M. (2025). A human pan-disease blood Atlas of the circulating proteome. Science 390 (6779), eadx2678. 10.1126/science.adx2678 41066540

[B13] Amin R. Simakajornboon N. Szczesniak R. Inge T. (2017). Early improvement in obstructive sleep apnea and increase in orexin levels after bariatric surgery in adolescents and young adults. Surg. Obes. Relat. Dis. 13 (1), 95–100. 10.1016/j.soard.2016.05.023 27720196 PMC5130623

[B14] An H. Cho M. H. Kim D. H. Chung S. Yoon S. Y. (2017). Orexin impairs the phagocytosis and degradation of amyloid-beta fibrils by microglial cells. J. Alzheimers Dis. 58 (1), 253–261. 10.3233/JAD-170108 28387679

[B15] Ardeljan A. D. Hurezeanu R. (2021). Lemborexant. Amsterdam, Netherlands: Elsevier.

[B16] Arihara Z. Takahashi K. Murakami O. Totsune K. Sone M. Satoh F. (2000). Orexin-a in the human brain and tumor tissues of ganglioneuroblastoma and neuroblastoma. Peptides 21 (4), 565–570. 10.1016/s0196-9781(00)00184-4 10822113

[B17] Arihara Z. Takahashi K. Murakami O. Totsune K. Sone M. Satoh F. (2001). Immunoreactive Orexin-a in human plasma. Peptides 22 (1), 139–142. 10.1016/s0196-9781(00)00369-7 11179609

[B18] Arnett A. (2025). The race toward orexin agonists targeting narcolepsy’s root cause. Sleep. Rev. Available online at: https://sleepreviewmag.com/sleep-treatments/pharmaceuticals/emerging-compounds/race-toward-orexin-agonists-targeting-narcolepsys-root-cause/ (Accesed July 14, 2025).

[B19] Arnolds S. Kuglin B. Kapitza C. Heise T. (2010). How pharmacokinetic and pharmacodynamic principles pave the way for optimal basal insulin therapy in type 2 diabetes. Int. J. Clin. Pract. 64 (10), 1415–1424. 10.1111/j.1742-1241.2010.02470.x 20618882 PMC2984539

[B20] Arslan G. A. Saygi S. Bodur E. Cicek C. Tezer F. I. (2022). Relation between orexin a and epileptic seizures. Epilepsy Res. 184, 106972. 10.1016/j.eplepsyres.2022.106972 35772324

[B21] Atacan Y. M. Ayca S. Demirbilek V. Saltik S. Yalcinkaya C. Erdogan D. Y. (2023). Serum levels of neuropeptides in epileptic encephalopathy with spike-and-wave activation in sleep. Pediatr. Neurol. 144, 110–114. 10.1016/j.pediatrneurol.2023.04.017 37229878

[B22] Baccari M. C. (2010). Orexins and gastrointestinal functions. Curr. Protein Pept. Sci. 11 (2), 148–155. 10.2174/138920310790848377 20353399

[B23] BACHEM. Peptide Calculator. (2025). Available online at: https://www.Bachem.Com/Knowledge-Center/Peptide-Calculator/ (Accesed July 2, 2025).

[B24] Baier P. C. Weinhold S. L. Huth V. Gottwald B. Ferstl R. Hinze-Selch D. (2008). Olfactory dysfunction in patients with Narcolepsy with cataplexy is restored by intranasal orexin a (Hypocretin-1). Brain 131 (10), 2734–2741. 10.1093/brain/awn193 18718966

[B25] Baier P. C. Hallschmid M. Seeck-Hirschner M. Weinhold S. L. Burkert S. Diessner N. (2011). Effects of intranasal Hypocretin-1 (Orexin a) on sleep in Narcolepsy with cataplexy. Sleep. Med. 12 (10), 941–946. 10.1016/j.sleep.2011.06.015 22036605

[B26] Barateau L. Dauvilliers Y. (2019). Recent advances in treatment for Narcolepsy. Ther. Adv. Neurol. Disord. 12, 1756286419875622. 10.1177/1756286419875622 31632459 PMC6767718

[B27] Basar M. M. Han U. Cakan M. Alpcan S. Basar H. (2013). Orexin expression in different prostate histopathologic examinations: can it be a marker for prostate cancer? A preliminary result. Turk J. Urol. 39 (2), 78–83. 10.5152/tud.2013.023 26328085 PMC4548592

[B28] Basoglu C. Oner O. Gunes C. Semiz U. B. Ates A. M. Algul A. (2010). Plasma orexin a, ghrelin, cholecystokinin, visfatin, leptin and agouti-related protein levels during 6-week olanzapine treatment in first-episode male patients with psychosis. Int. Clin. Psychopharmacol. 25 (3), 165–171. 10.1097/YIC.0b013e3283377850 21811193

[B29] Baumann C. R. Clark E. L. Pedersen N. P. Hecht J. L. Scammell T. E. (2008). Do enteric neurons make hypocretin? Regul. Pept. 147 (1-3), 1–3. 10.1016/j.regpep.2007.11.006 18191238 PMC2276606

[B30] Baykal S. Albayrak Y. Durankus F. Guzel S. Abbak O. Potas N. (2019). Decreased serum orexin a levels in drug-naive children with attention deficit and hyperactivity disorder. Neurol. Sci. 40 (3), 593–602. 10.1007/s10072-018-3692-8 30617449

[B31] Becquet L. Abad C. Leclercq M. Miel C. Jean L. Riou G. (2019). Systemic administration of orexin a ameliorates established experimental autoimmune encephalomyelitis by diminishing neuroinflammation. J. Neuroinflammation 16 (1), 64. 10.1186/s12974-019-1447-y 30894198 PMC6425555

[B32] Ben M. R. Khodadadi-Mericle F. Kline D. D. Hasser E. M. Cummings K. J. (2026). Orexin facilitates the peripheral chemoreflex in the active phase *via* corticotropin-releasing hormone neurons that Project to the nucleus of the solitary tract. Funct. (Oxf) 7 (1), e0842025. 10.1152/function.084.2025 PMC12850509 41401425

[B33] Bigalke J. A. Shan Z. Carter J. R. (2022). Orexin, sleep, sympathetic neural activity, and cardiovascular function. Hypertension 79 (12), 2643–2655. 10.1161/HYPERTENSIONAHA.122.19796 36148653 PMC9649879

[B34] Bilir C. Engin H. Can M. Temi Y. B. Demirtas D. (2015). The prognostic role of inflammation and hormones in patients with metastatic cancer with Cachexia. Med. Oncol. 32 (3), 56. 10.1007/s12032-015-0497-y 25638467

[B35] Blais A. Lan A. Blachier F. Benamouzig R. Jouet P. Couvineau A. (2023). Efficiency of Orexin-a for inflammatory flare and mucosal healing in experimental colitis: comparison with the anti-Tnf alpha Infliximab. Int. J. Mol. Sci. 24 (11), 9554. 10.3390/ijms24119554 37298505 PMC10253642

[B36] Blanco M. Lopez M. GarcIa-Caballero T. Gallego R. Vazquez-Boquete A. Morel G. (2001). Cellular localization of orexin receptors in human pituitary. J. Clin. Endocrinol. Metab. 86 (7), 1616–1619. 10.1210/jc.86.4.1616 11297593

[B37] Blanco M. Garcia-Caballero T. Fraga M. Gallego R. Cuevas J. Forteza J. (2002). Cellular localization of orexin receptors in human adrenal gland, adrenocortical adenomas and pheochromocytomas. Regul. Pept. 104 (1-3), 161–165. 10.1016/s0167-0115(01)00359-7 11830291

[B38] Blanco M. Gallego R. Garcia-Caballero T. Dieguez C. Beiras A. (2003). Cellular localization of orexins in human anterior pituitary. Histochem Cell Biol. 120 (4), 259–264. 10.1007/s00418-003-0562-z 14574580

[B39] Bonvalet M. Ollila H. M. Ambati A. Mignot E. (2017). Autoimmunity in narcolepsy. Curr. Opin. Pulm. Med. 23 (6), 522–529. 10.1097/MCP.0000000000000426 28991006 PMC5773260

[B40] Bragg S. Benich J. J. Christian N. Visserman J. Freedy J. (2019). Updates in insomnia diagnosis and treatment. Int. J. Psychiatry Med. 54 (4-5), 275–289. 10.1177/0091217419860716 31269837

[B41] Bronsky J. Nedvidkova J. Zamrazilova H. Pechova M. Chada M. Kotaska K. (2007). Dynamic changes of orexin a and Leptin in obese children during body weight reduction. Physiol. Res. 56 (1), 89–96. 10.33549/physiolres.930860 16497092

[B42] Bronsky J. Nedvidkova J. Krasnicanova H. Vesela M. Schmidtova J. Koutek J. (2011). Changes of orexin a plasma levels in girls with anorexia nervosa during eight weeks of realimentation. Int. J. Eat. Disord. 44 (6), 547–552. 10.1002/eat.20857 21823139

[B43] Brunton P. J. Russell J. A. (2003). Hypothalamic-pituitary-adrenal responses to centrally administered Orexin-a are suppressed in pregnant rats. J. Neuroendocrinol. 15 (7), 633–637. 10.1046/j.1365-2826.2003.01045.x 12787047

[B44] Brzozowski T. Konturek P. C. Sliwowski Z. Drozdowicz D. Burnat G. Pajdo R. (2008). Gastroprotective action of Orexin-a against stress-induced gastric damage is mediated by endogenous prostaglandins, sensory afferent neuropeptides and nitric oxide. Regul. Pept. 148 (1-3), 6–20. 10.1016/j.regpep.2008.02.003 18378017

[B45] Bucsek M. J. Giridharan T. MacDonald C. R. Hylander B. L. Repasky E. A. (2018). An overview of the role of sympathetic regulation of immune responses in infectious disease and autoimmunity. Int. J. Hyperth. 34 (2), 135–143. 10.1080/02656736.2017.1411621 29498310 PMC6309867

[B46] Bulbul M. Tan R. Gemici B. Ongut G. Izgut-Uysal V. N. (2008). Effect of Orexin-a on ischemia-reperfusion-induced gastric damage in rats. J. Gastroenterol. 43 (3), 202–207. 10.1007/s00535-007-2148-3 18373162

[B47] Busquets X. Barbe F. Barcelo A. de la Pena M. Sigritz N. Mayoralas L. R. (2004). Decreased plasma levels of Orexin-a in sleep apnea. Respiration 71 (6), 575–579. 10.1159/000081757 15627867

[B48] Butterick T. A. Nixon J. P. Billington C. J. Kotz C. M. (2012). Orexin a decreases lipid peroxidation and apoptosis in a novel hypothalamic cell model. Neurosci. Lett. 524 (1), 30–34. 10.1016/j.neulet.2012.07.002 22796468 PMC4467811

[B49] Calva C. B. Fayyaz H. Fadel J. R. (2018). Increased acetylcholine and glutamate efflux in the prefrontal cortex following intranasal Orexin-a (Hypocretin-1). J. Neurochem. 145 (3), 232–244. 10.1111/jnc.14279 29250792 PMC5924451

[B50] Calva C. B. Fayyaz H. Fadel J. R. (2019). Effects of intranasal Orexin-a (Hypocretin-1) administration on neuronal activation, neurochemistry, and attention in aged rats. Front. Aging Neurosci. 11, 362. 10.3389/fnagi.2019.00362 32038222 PMC6987046

[B51] Caproni S. Corbelli I. Pini L. A. Cupini M. L. Calabresi P. Sarchielli P. (2011). Migraine preventive drug-induced weight gain may be mediated by effects on hypothalamic peptides: the results of a pilot Study. Cephalalgia 31 (5), 543–549. 10.1177/0333102410392605 21216871

[B52] Carpi M. Mercuri N. B. Liguori C. (2024). Orexin receptor antagonists for the prevention and treatment of Alzheimer’s disease and associated sleep disorders. Drugs 84 (11), 1365–1378. 10.1007/s40265-024-02096-3 39365407 PMC11602839

[B53] Chang H. Saito T. Ohiwa N. Tateoka M. Deocaris C. C. Fujikawa T. (2007). Inhibitory effects of an Orexin-2 receptor antagonist on orexin a- and stress-induced acth responses in conscious rats. Neurosci. Res. 57 (3), 462–466. 10.1016/j.neures.2006.11.009 17188385

[B54] Chang X. Zhao Y. Ju S. Guo L. (2014). Orexin-a stimulates 3beta-hydroxysteroid dehydrogenase expression and cortisol production in H295r human adrenocortical cells through the Akt pathway. Int. J. Mol. Med. 34 (6), 1523–1528. 10.3892/ijmm.2014.1959 25319929

[B55] Chang X. Suo L. Xu N. Zhao Y. (2019). Orexin-a stimulates insulin secretion through the activation of the Ox1 receptor and mammalian target of rapamycin in rat insulinoma cells. Pancreas 48 (4), 568–573. 10.1097/MPA.0000000000001280 30946236

[B56] Chen J. Randeva H. S. (2004). Genomic organization of mouse orexin receptors: characterization of two novel tissue-specific splice variants. Mol. Endocrinol. 18 (11), 2790–2804. 10.1210/me.2004-0167 15256537

[B57] Chen J. Randeva H. S. (2010). Genomic organization and regulation of the human orexin (Hypocretin) receptor 2 gene: identification of alternative promoters. Biochem. J. 427 (3), 377–390. 10.1042/BJ20091755 20156195

[B58] Chen J. Karteris E. Collins D. Randeva H. S. (2006). Differential expression of mouse orexin receptor Type-2 (Ox2r) variants in the mouse brain. Brain Res. 1103 (1), 20–24. 10.1016/j.brainres.2006.05.054 16806113

[B59] Chen J. Zhang R. Chen X. Wang C. Cai X. Liu H. (2015). Heterodimerization of human orexin receptor 1 and Kappa opioid receptor promotes protein kinase a/Camp-response element binding protein signaling *via* a galphas-mediated mechanism. Cell Signal 27 (7), 1426–1438. 10.1016/j.cellsig.2015.03.027 25866368

[B60] Chen W. Y. Kao C. F. Chen P. Y. Lin S. K. Huang M. C. (2016). Orexin-a level elevation in recently abstinent Male methamphetamine abusers. Psychiatry Res. 239, 9–11. 10.1016/j.psychres.2016.02.059 27137956

[B61] Chen Z. Q. Mou R. T. Feng D. X. Wang Z. Chen G. (2017). The role of nitric oxide in stroke. Med. Gas. Res. 7 (3), 194–203. 10.4103/2045-9912.215750 29152213 PMC5674658

[B62] Chen P. Y. Chen C. H. Chang C. K. Kao C. F. Lu M. L. Lin S. K. (2019). Orexin-a levels in relation to the risk of metabolic syndrome in patients with schizophrenia taking antipsychotics. Int. J. Neuropsychopharmacol. 22 (1), 28–36. 10.1093/ijnp/pyy075 30204875 PMC6313111

[B63] Chen P. Y. Chang C. K. Chen C. H. Fang S. C. Mondelli V. Chiu C. C. (2022). Orexin-a elevation in antipsychotic-treated compared to drug-free patients with schizophrenia: a medication effect independent of Metabolic syndrome. J. Formos. Med. Assoc. 121 (11), 2172–2181. 10.1016/j.jfma.2022.03.008 35396156

[B64] Chen C. L. Syahirah R. Ravala S. K. Yen Y. C. Klose T. Deng Q. (2024). Molecular basis for Gβγ-mediated activation of phosphoinositide 3-kinase γ. Nat. Struct. Mol. Biol. 31, 1198–1207. 10.1038/s41594-024-01265-y 38565696 PMC11329362

[B65] Chien Y. L. Liu C. M. Shan J. C. Lee H. J. Hsieh M. H. Hwu H. G. (2015). Elevated plasma orexin a levels in a subgroup of patients with schizophrenia associated with fewer negative and disorganized symptoms. Psychoneuroendocrinology 53, 1–9. 10.1016/j.psyneuen.2014.12.012 25560205

[B66] Chino Y. Sugiyama H. Chaki S. Sato Y. (2025). Current and novel dual orexin receptor antagonists for the treatment of insomnia: the emergence of vornorexant. Int. J. Neuropsychopharmacol. 28 (12), pyaf077. 10.1093/ijnp/pyaf077 41273062 PMC13223731

[B67] Cho G. J. Han S. W. Shin J. H. Kim T. (2017). Effects of intensive training on menstrual function and certain serum hormones and peptides related to the female reproductive system. Med. Baltim. 96 (21), e6876. 10.1097/MD.0000000000006876 28538378 PMC5457858

[B68] Choi M. R. Cho H. Chun J. W. Yoo J. H. Kim D. J. (2020). Increase of orexin a in the peripheral blood of adolescents with internet gaming disorder. J. Behav. Addict. 9 (1), 93–104. 10.1556/2006.8.2019.65 31957460 PMC8935190

[B69] Cintron D. Beckman J. P. Bailey K. R. Lahr B. D. Jayachandran M. Miller V. M. (2017). Plasma orexin a levels in recently menopausal women during and 3 years following use of hormone therapy. Maturitas 99, 59–65. 10.1016/j.maturitas.2017.01.016 28364870 PMC5588667

[B70] Cioffi I. Gambino R. Rosato R. Properzi B. Regaldo G. Ponzo V. (2020). Acute assessment of subjective appetite and implicated hormones after a hypnosis-induced hallucinated meal: a randomized cross-over pilot trial. Rev. Endocr. Metab. Disord. 21 (3), 411–420. 10.1007/s11154-020-09559-4 32418064

[B71] Coban A. Bilgic B. Lohmann E. Kucukali C. I. Benbir G. Karadeniz D. (2013). Reduced Orexin-a levels in frontotemporal dementia: possible association with sleep disturbance. Am. J. Alzheimers Dis. Other Demen 28 (6), 606–611. 10.1177/1533317513494453 23813609 PMC10852656

[B72] Costagliola A. Montano L. Langella E. Lombardi R. Squillacioti C. Mirabella N. (2024). A comparative analysis of orexins in the physio-pathological processes of the Male genital tract: new challenges? A review. Vet. Sci. 11 (3), 131. 10.3390/vetsci11030131 38535865 PMC10975741

[B73] Costello A. Linning-Duffy K. Shi J. Lonstein J. S. Yan L. (2026). Central Hypocretin/Orexin administration alleviates Sleep/Wake disturbances, Anhedonia, and neuroinflammation in an animal model of seasonal affective disorder. Ann. Med. 58 (1), 2611202. 10.1080/07853890.2025.2611202 41508567 PMC12794707

[B74] Couvineau A. Voisin T. Nicole P. Gratio V. Blais A. (2021). Orexins: a promising target to digestive cancers, inflammation, obesity and metabolism dysfunctions. World J. Gastroenterol. 27 (44), 7582–7596. 10.3748/wjg.v27.i44.7582 34908800 PMC8641057

[B75] Couvineau A. Nicole P. Gratio V. Voisin T. (2022). The orexin receptors: structural and anti-tumoral properties. Front. Endocrinol. (Lausanne) 13, 931970. 10.3389/fendo.2022.931970 35966051 PMC9365956

[B76] Dalal M. A. Schuld A. Haack M. Uhr M. Geisler P. Eisensehr I. (2001). Normal plasma levels of orexin a (Hypocretin-1) in narcoleptic patients. Neurology 56 (12), 1749–1751. 10.1212/wnl.56.12.1749 11425946

[B77] Dale N. C. Hoyer D. Jacobson L. H. Pfleger K. D. G. Johnstone E. K. M. (2022). Orexin signaling: a complex, multifaceted process. Front. Cell Neurosci. 16, 812359. 10.3389/fncel.2022.812359 35496914 PMC9044999

[B78] Dale N. C. Purbrick H. G. Pfleger K. D. Johnstone E. K. (2026). Orexin receptor 2 (Ox(2)R) exhibits ligand-dependent spatio-temporal pharmacology through Galpha(I):Beta-Arrestin dynamics in response to Orexin-a and Orexin-B. Biochem. Pharmacol. 243 (Pt 2), 117517. 10.1016/j.bcp.2025.117517 41203035

[B79] Date Y. Ueta Y. Yamashita H. Yamaguchi H. Matsukura S. Kangawa K. (1999). Orexins, orexigenic hypothalamic peptides, interact with autonomic, neuroendocrine and neuroregulatory systems. Proc. Natl. Acad. Sci. U. S. A. 96 (2), 748–753. 10.1073/pnas.96.2.748 9892705 PMC15208

[B80] Dauvilliers Y. Mignot E. Del Rio Villegas R. Du Y. Hanson E. Inoue Y. (2023). Oral Orexin receptor 2 agonist in narcolepsy type 1. N. Engl. J. Med. 389 (4), 309–321. 10.1056/NEJMoa2301940 37494485

[B81] Dauvilliers Y. Plazzi G. Mignot E. Lammers G. J. Del Rio Villegas R. Khatami R. (2025). Oveporexton, an oral orexin receptor 2-Selective agonist, in Narcolepsy type 1. N. Engl. J. Med. 392 (19), 1905–1916. 10.1056/NEJMoa2405847 40367374

[B82] Dayot S. Speisky D. Couvelard A. Bourgoin P. Gratio V. Cros J. (2018). *In vitro,*, *in vivo* and *Ex Vivo* demonstration of the antitumoral role of Hypocretin-1/Orexin-a and almorexant in pancreatic ductal adenocarcinoma. Oncotarget 9 (6), 6952–6967. 10.18632/oncotarget.24084 29467942 PMC5805528

[B83] de Jonge W. J. (2013). The gut's little brain in control of intestinal immunity. ISRN Gastroenterol. 2013, 630159. 10.1155/2013/630159 23691339 PMC3649343

[B84] de Lecea L. Kilduff T. S. Peyron C. Gao X. Foye P. E. Danielson P. E. (1998). The hypocretins: hypothalamus-specific peptides with neuroexcitatory activity. Proc. Natl. Acad. Sci. U. S. A. 95 (1), 322–327. 10.1073/pnas.95.1.322 9419374 PMC18213

[B85] Deadwyler S. A. Porrino L. Siegel J. M. Hampson R. E. (2007). Systemic and nasal delivery of Orexin-a (Hypocretin-1) reduces the effects of sleep deprivation on cognitive performance in nonhuman primates. J. Neurosci. 27 (52), 14239–14247. 10.1523/JNEUROSCI.3878-07.2007 18160631 PMC6673447

[B86] Dehan P. Canon C. Trooskens G. Rehli M. Munaut C. Van Criekinge W. (2013). Expression of type 2 orexin receptor in human endometrium and its epigenetic silencing in endometrial cancer. J. Clin. Endocrinol. Metab. 98 (4), 1549–1557. 10.1210/jc.2012-3263 23482607

[B87] Deloumeau A. Bayard S. Coquerel Q. Dechelotte P. Bole-Feysot C. Carlander B. (2010). Increased immune complexes of hypocretin autoantibodies in Narcolepsy. PLoS One 5 (10), e13320. 10.1371/journal.pone.0013320 20967199 PMC2954157

[B88] Dergacheva O. Yamanaka A. Schwartz A. R. Polotsky V. Y. Mendelowitz D. (2017). Optogenetic identification of hypothalamic Orexin neuron projections to paraventricular spinally projecting neurons. Am. J. Physiol. Heart Circ. Physiol. 312 (4), H808–H817. 10.1152/ajpheart.00572.2016 28159808 PMC5407165

[B89] Deutschman C. S. Raj N. R. McGuire E. O. Kelz M. B. (2013). Orexinergic activity modulates altered vital signs and pituitary hormone secretion in experimental sepsis. Crit. Care Med. 41 (11), e368–e375. 10.1097/CCM.0b013e31828e9843 24105451 PMC6880745

[B90] Dhuria S. V. Hanson L. R. Frey W. H. 2nd (2009). Intranasal drug targeting of hypocretin-1 (Orexin-a) to the central nervous system. J. Pharm. Sci. 98 (7), 2501–2515. 10.1002/jps.21604 19025760

[B91] Dhuria S. V. Fine J. M. Bingham D. Svitak A. L. Burns R. B. Baillargeon A. M. (2016). Food consumption and activity levels increase in rats following intranasal hypocretin-1. Neurosci. Lett. 627, 155–159. 10.1016/j.neulet.2016.05.053 27264485

[B92] Dobrzyn K. Smolinska N. Kiezun M. Szeszko K. Rytelewska E. Kisielewska K. (2018). The *in vitro* effect of progesterone on the orexin system in porcine uterine tissues during early pregnancy. Acta Vet. Scand. 60 (1), 76. 10.1186/s13028-018-0430-4 30477546 PMC6258494

[B93] Dong J. Zhang J. Cheng S. Qin B. Jin K. Chen B. (2024). A high-fat diet induced depression-like phenotype *via* hypocretin-Hcrtr1 mediated inflammation activation. Food Funct. 15 (17), 8661–8673. 10.1039/d4fo00210e 39056112

[B94] Duffy C. M. Yuan C. Wisdorf L. E. Billington C. J. Kotz C. M. Nixon J. P. (2015). Role of orexin a signaling in dietary palmitic acid-activated microglial cells. Neurosci. Lett. 606, 140–144. 10.1016/j.neulet.2015.08.033 26306651 PMC4811357

[B95] Duffy C. M. Nixon J. P. Butterick T. A. (2016). Orexin a attenuates palmitic acid-induced hypothalamic cell death. Mol. Cell Neurosci. 75, 93–100. 10.1016/j.mcn.2016.07.003 27449757 PMC5399885

[B96] Duffy C. M. Hofmeister J. J. Nixon J. P. Butterick T. A. (2019). High fat diet increases cognitive decline and neuroinflammation in a model of orexin loss. Neurobiol. Learn Mem. 157, 41–47. 10.1016/j.nlm.2018.11.008 30471346 PMC6547825

[B97] Durairaja A. Pandey S. Kahl E. Fendt M. (2023). Nasal administration of orexin a partially rescues dizocilpine-induced cognitive impairments in female C57bl/6 J mice. Behav. Brain Res. 450, 114491. 10.1016/j.bbr.2023.114491 37172740

[B98] Ehrstrom M. Naslund E. Levin F. Kaur R. Kirchgessner A. L. Theodorsson E. (2004). Pharmacokinetic profile of orexin a and effects on plasma insulin and glucagon in the rat. Regul. Pept. 119 (3), 209–212. 10.1016/j.regpep.2004.02.004 15120482

[B99] Ehrstrom M. Gustafsson T. Finn A. Kirchgessner A. Gryback P. Jacobsson H. (2005a). Inhibitory effect of exogenous orexin a on gastric emptying, plasma leptin, and the distribution of orexin and orexin receptors in the gut and pancreas in man. J. Clin. Endocrinol. Metab. 90 (4), 2370–2377. 10.1210/jc.2004-1408 15671114

[B100] El-Sedeek M. Korish A. A. Deef M. M. (2010). Plasma Orexin-a levels in postmenopausal women: possible interaction with estrogen and correlation with cardiovascular risk status. BJOG 117 (4), 488–492. 10.1111/j.1471-0528.2009.02474.x 20105164

[B101] Ellis J. Pediani J. D. Canals M. Milasta S. Milligan G. (2006). Orexin-1 receptor-cannabinoid Cb1 receptor Heterodimerization results in both ligand-dependent and -Independent coordinated alterations of receptor localization and function. J. Biol. Chem. 281 (50), 38812–38824. 10.1074/jbc.M602494200 17015451

[B102] EMA. Union register of medicinal products for human use. (2025). Available online at: https://ec.europa.eu/health/documents/community-register/html/h1638.htm (Accesed March 2, 2025).

[B103] Fatemi I. Shamsizadeh A. Ayoobi F. Taghipour Z. Sanati M. H. Roohbakhsh A. (2016). Role of Orexin-a in experimental autoimmune encephalomyelitis. J. Neuroimmunol. 291, 101–109. 10.1016/j.jneuroim.2016.01.001 26857503

[B104] Fernandez-Rilo A. C. Forte N. Palomba L. Tunisi L. Piscitelli F. Imperatore R. (2023). Orexin induces the production of an endocannabinoid-derived lysophosphatidic acid eliciting hypothalamic synaptic loss in obesity. Mol. Metab. 72, 101713. 10.1016/j.molmet.2023.101713 36977433 PMC10122056

[B105] Forte N. Fernandez-Rilo A. C. Palomba L. Marfella B. Piscitelli F. De Girolamo P. (2022). Positive association between plasmatic levels of orexin a and the endocannabinoid-derived 2-Arachidonoyl lysophosphatidic acid in Alzheimer’s disease. Front. Aging Neurosci. 14, 1004002. 10.3389/fnagi.2022.1004002 36466600 PMC9710385

[B106] Frick M. A. Woodruff J. L. Caudillo Y. M. Pikel K. E. Rehm J. V. Maciejewska N. (2025). Orexin/hypocretin modulates neuroinflammatory response to Lps in a sex and brain-region specific manner in young rats. J. Neurochem. 169 (8), e70175. 10.1111/jnc.70175 40728035 PMC12305754

[B107] Gabelle A. Jaussent I. Bouallegue F. B. Lehmann S. Lopez R. Barateau L. (2019). Reduced brain amyloid burden in elderly patients with Narcolepsy type 1. Ann. Neurol. 85 (1), 74–83. 10.1002/ana.25373 30387527

[B108] Gan J. Liu S. Chen Z. Yang Y. Ma L. Meng Q. (2022). Elevated plasma Orexin-a levels in prodromal dementia with lewy bodies. J. Alzheimers Dis. 88 (3), 1037–1048. 10.3233/JAD-220082 35723094

[B109] Geerling J. C. Mettenleiter T. C. Loewy A. D. (2003). Orexin neurons project to diverse sympathetic outflow systems. Neuroscience 122 (2), 541–550. 10.1016/j.neuroscience.2003.07.008 14614918

[B110] Gencer M. Akbayir E. Sen M. Arsoy E. Yilmaz V. Bulut N. (2019). Serum Orexin-a levels are associated with disease progression and motor impairment in multiple sclerosis. Neurol. Sci. 40 (5), 1067–1070. 10.1007/s10072-019-3708-z 30645749

[B111] Gerashchenko D. Shiromani P. J. (2004). Effects of inflammation produced by chronic Lipopolysaccharide administration on the survival of hypocretin neurons and sleep. Brain Res. 1019 (1-2), 162–169. 10.1016/j.brainres.2004.06.016 15306250

[B112] Gratio V. Dragan P. Garcia L. Saveanu L. Nicole P. Voisin T. (2025). Pharmacodynamics of the orexin type 1 (Ox(1)) receptor in Colon cancer cell models: a two-sided nature of antagonistic ligands resulting from partial dissociation of Gq. Br. J. Pharmacol. 182 (7), 1528–1545. 10.1111/bph.17422 39675769

[B113] Greco R. Demartini C. Zanaboni A. M. Blandini F. Amantea D. Tassorelli C. (2018). Endothelial nitric oxide synthase inhibition triggers inflammatory responses in the brain of Male rats exposed to ischemia-reperfusion injury. J. Neurosci. Res. 96 (1), 151–159. 10.1002/jnr.24101 28609584

[B114] Grossberg A. J. Zhu X. Leinninger G. M. Levasseur P. R. Braun T. P. Myers M. G. Jr. (2011). Inflammation-induced lethargy is mediated by suppression of orexin neuron activity. J. Neurosci. 31 (31), 11376–11386. 10.1523/JNEUROSCI.2311-11.2011 21813697 PMC3155688

[B115] Gultuna S. Basa Akdogan B. Gonul M. Aydin F. N. Unal S. Erkek G. N. (2024). Sleep quality in patients with chronic spontaneous urticaria and relation with Orexin-a, Leptin, and ghrelin. Allergy Asthma Proc. 45 (4), e38–e45. 10.2500/aap.2024.45.240023 38982607

[B116] Guo J. Zhu T. Luo L. Y. Huang Y. Sunkavalli R. G. Chen C. Y. (2009). Pi3k acts in synergy with loss of pkc to elicit apoptosis *via* the upr. J. Cell Biochem. 107 (1), 76–85. 10.1002/jcb.22102 19241442 PMC3545399

[B117] Guo L. Hu A. Zhao X. Xiang X. (2022). Reduction of Orexin-a is associated with anxiety and the level of depression of Male methamphetamine users during the initial withdrawal period. Front. Psychiatry 13, 900135. 10.3389/fpsyt.2022.900135 35859609 PMC9289462

[B118] Guo X. Wen J. Gao Q. Zhao Y. Zhao Y. Wang C. (2024a). Orexin-a/Ox1r is involved in regulation of autophagy to promote cortisol secretion in adrenocortical cell. Biochim. Biophys. Acta Mol. Basis Dis. 1870 (1), 166844. 10.1016/j.bbadis.2023.166844 37572990

[B119] Guo J. Kong Z. Yang S. Da J. Chu L. Han G. (2024b). Therapeutic effects of Orexin-a in sepsis-associated encephalopathy in mice. J. Neuroinflammation 21 (1), 131. 10.1186/s12974-024-03111-w 38760784 PMC11102217

[B120] Guo J. Kong D. Luo J. Xiong T. Wang F. Deng M. (2024c). Orexin-a attenuates the inflammatory response in sepsis-associated encephalopathy by modulating oxidative stress and inhibiting the Erk/Nf-Kappab signaling pathway in microglia and astrocytes. CNS Neurosci. Ther. 30 (11), e70096. 10.1111/cns.70096 39508266 PMC11541240

[B121] Gupta A. Miegueu P. Lapointe M. Poirier P. Martin J. Bastien M. (2014). Acute post-bariatric surgery increase in orexin levels associates with preferential lipid profile improvement. PLoS One 9 (1), e84803. 10.1371/journal.pone.0084803 24400115 PMC3882247

[B122] Haass-Koffler C. L. Perciballi R. Brown Z. E. Lee M. R. Zywiak W. H. Kurtis J. (2021). Brief report: relationship between cotinine levels and peripheral endogenous concentrations of oxytocin, beta-endorphin, and orexin in individuals with both alcohol and nicotine use disorders. Am. J. Addict. 30 (1), 88–91. 10.1111/ajad.13064 32488890 PMC7944578

[B123] Hada K. Murata Y. Ohi Y. Hashimoto S. Watanabe H. Ozeki K. (2026). Involvement of orexin nerves in early stage of Alzheimer’s disease model mice and preventive effect of orexin receptor antagonists. J. Alzheimers Dis. 110 (2), 588–604. 10.1177/13872877261415630 41815067

[B124] Hao Y. Y. Yuan H. W. Fang P. H. Zhang Y. Liao Y. X. Shen C. (2017). Plasma Orexin-a level associated with physical activity in Obese people. Eat. Weight Disord. 22 (1), 69–77. 10.1007/s40519-016-0271-y 27038345

[B125] Hartmann F. J. Bernard-Valnet R. Queriault C. Mrdjen D. Weber L. M. Galli E. (2016). High-dimensional single-cell analysis reveals the immune signature of narcolepsy. J. Exp. Med. 213 (12), 2621–2633. 10.1084/jem.20160897 27821550 PMC5110028

[B126] Heinonen M. V. Purhonen A. K. Miettinen P. Paakkonen M. Pirinen E. Alhava E. (2005). Apelin, Orexin-a and leptin plasma levels in morbid obesity and effect of gastric banding. Regul. Pept. 130 (1-2), 7–13. 10.1016/j.regpep.2005.05.003 15970339

[B127] Herr N. Bode C. Duerschmied D. (2017). The effects of serotonin in immune cells. Front. Cardiovasc Med. 4, 48. 10.3389/fcvm.2017.00048 28775986 PMC5517399

[B128] Herring W. J. Connor K. M. Snyder E. Snavely D. B. Zhang Y. Hutzelmann J. (2017). Suvorexant in elderly patients with insomnia: pooled analyses of data from phase iii randomized controlled clinical trials. Am. J. Geriatr. Psychiatry 25 (7), 791–802. 10.1016/j.jagp.2017.03.004 28427826

[B129] Higuchi S. Usui A. Murasaki M. Matsushita S. Nishioka N. Yoshino A. (2002). Plasma Orexin-a is lower in patients with narcolepsy. Neurosci. Lett. 318 (2), 61–64. 10.1016/s0304-3940(01)02476-4 11796186

[B130] Hilairet S. Bouaboula M. Carriere D. Le Fur G. Casellas P. (2003). Hypersensitization of the orexin 1 receptor by the Cb1 receptor: evidence for cross-talk blocked by the specific Cb1 antagonist, Sr141716. J. Biol. Chem. 278 (26), 23731–23737. 10.1074/jbc.M212369200 12690115

[B131] Hm A. E. Kruse L. Christensen G. L. Lorentzen M. P. Jorgensen N. R. Moresco M. (2021). Pre-treatment of blood samples reveal normal blood hypocretin/orexin signal in narcolepsy type 1. Brain Commun. 3 (2), fcab050. 10.1093/braincomms/fcab050 33977264 PMC8100001

[B132] Hoch M. van Gorsel H. van Gerven J. Dingemanse J. (2014). Entry-into-humans study with Act-462206, a novel dual orexin receptor antagonist, comparing its pharmacodynamics with almorexant. J. Clin. Pharmacol. 54 (9), 979–986. 10.1002/jcph.297 24691844

[B133] Holth J. K. Fritschi S. K. Wang C. Pedersen N. P. Cirrito J. R. Mahan T. E. (2019). The sleep-wake cycle regulates brain interstitial fluid tau in mice and Csf tau in humans. Science 363 (6429), 880–884. 10.1126/science.aav2546 30679382 PMC6410369

[B134] Hopf F. W. (2020). Recent perspectives on Orexin/Hypocretin promotion of addiction-related behaviors. Neuropharmacology 168, 108013. 10.1016/j.neuropharm.2020.108013 32092435

[B135] Horiuchi D. Irukayama-Tomobe Y. Kim J. D. Kasuya Y. Nemoto T. Suzuki T. (2026). Orexin receptor antagonism improves sleep quality and mitigates lipopolysaccharide-induced inflammatory responses in a mouse model. FASEB J. 40 (1), e71408. 10.1096/fj.202502960R 41511173 PMC12785484

[B136] Hu S. Niu J. Zhang R. Li X. Luo M. Sang T. (2020). Orexin a associates with inflammation by interacting with Ox1r/Ox2r receptor and activating prepro-orexin in cancer tissues of gastric cancer patients. Gastroenterol. Hepatol. 43 (5), 240–247. 10.1016/j.gastrohep.2019.10.006 31983458

[B137] Hu S. Ren L. Wang Y. Lei Z. Cai J. Pan S. (2023). The Association between serum orexin a and short-term neurological improvement in patients with mild to moderate acute ischemic stroke. Brain Behav. 13 (1), e2845. 10.1002/brb3.2845 36573700 PMC9847589

[B138] Huang S. Zhao Z. Ma J. Hu S. Li L. Wang Z. (2021). Increased plasma Orexin-a concentrations are associated with the non-motor symptoms in Parkinson’s disease patients. Neurosci. Lett. 741, 135480. 10.1016/j.neulet.2020.135480 33161104

[B139] Hui H. Farilla L. Merkel P. Perfetti R. (2002). The short half-life of glucagon-like peptide-1 in plasma does not reflect its long-lasting beneficial effects. Eur. J. Endocrinol. 146 (6), 863–869. 10.1530/eje.0.1460863 12039708

[B140] Huston J. M. Ochani M. Rosas-Ballina M. Liao H. Ochani K. Pavlov V. A. (2006). Splenectomy inactivates the cholinergic antiinflammatory pathway during lethal endotoxemia and polymicrobial sepsis. J. Exp. Med. 203 (7), 1623–1628. 10.1084/jem.20052362 16785311 PMC2118357

[B141] Ida T. Nakahara K. Murakami T. Hanada R. Nakazato M. Murakami N. (2000). Possible involvement of orexin in the stress reaction in rats. Biochem. Biophys. Res. Commun. 270 (1), 318–323. 10.1006/bbrc.2000.2412 10733946

[B142] Igarashi N. Tatsumi K. Nakamura A. Sakao S. Takiguchi Y. Nishikawa T. (2003). Plasma Orexin-a levels in obstructive sleep apnea-hypopnea syndrome. Chest 124 (4), 1381–1385. 10.1378/chest.124.4.1381 14555569

[B143] Igarashi S. Nozu T. Ishioh M. Kumei S. Saito T. Toki Y. (2020). Centrally administered orexin prevents lipopolysaccharide and colchicine induced lethality *via* the vagal cholinergic pathway in a sepsis model in rats. Biochem. Pharmacol. 182, 114262. 10.1016/j.bcp.2020.114262 33035510

[B144] Inutsuka A. Yamanaka A. (2013). The physiological role of orexin/hypocretin neurons in the regulation of sleep/Wakefulness and neuroendocrine functions. Front. Endocrinol. (Lausanne) 4, 18. 10.3389/fendo.2013.00018 23508038 PMC3589707

[B145] Irving E. A. Harrison D. C. Babbs A. J. Mayes A. C. Campbell C. A. Hunter A. J. (2002). Increased cortical expression of the Orexin-1 receptor following permanent middle cerebral artery occlusion in the rat. Neurosci. Lett. 324 (1), 53–56. 10.1016/s0304-3940(02)00176-3 11983293

[B146] Ito N. Yabe T. Gamo Y. Nagai T. Oikawa T. Yamada H. (2008). Administration of Orexin-a induces an antidepressive-like effect through hippocampal cell proliferation. Neuroscience 157 (4), 720–732. 10.1016/j.neuroscience.2008.09.042 18952152

[B147] Izgut-Uysal V. N. Gemici B. Tan R. (2011). Effect of Orexin-a on phagocytic activity of peritoneal macrophage in starved rats. Cell Immunol. 271 (1), 85–88. 10.1016/j.cellimm.2011.06.005 21741630

[B148] Jacobson L. H. Hoyer D. de Lecea L. (2022). Hypocretins (orexins): the ultimate translational neuropeptides. J. Intern. Med. 291 (5), 533–556. 10.1111/joim.13406 35043499

[B149] Jaeger W. C. Seeber R. M. Eidne K. A. Pfleger K. D. (2014). Molecular determinants of orexin receptor-arrestin-ubiquitin complex formation. Br. J. Pharmacol. 171 (2), 364–374. 10.1111/bph.12481 24206104 PMC3904257

[B150] Jantti M. H. Mandrika I. Kukkonen J. P. (2014). Human orexin/hypocretin receptors form constitutive homo- and heteromeric complexes with each other and with human Cb1 cannabinoid receptors. Biochem. Biophys. Res. Commun. 445 (2), 486–490. 10.1016/j.bbrc.2014.02.026 24530395

[B151] Jaszberenyi M. Bujdoso E. Pataki I. Telegdy G. (2000). Effects of orexins on the hypothalamic-pituitary-adrenal system. J. Neuroendocrinol. 12 (12), 1174–1178. 10.1046/j.1365-2826.2000.00572.x 11106974

[B152] Jha M. K. (2022). Selective orexin receptor antagonists as novel augmentation treatments for major depressive disorder: evidence for safety and efficacy from a phase 2b study of seltorexant. Int. J. Neuropsychopharmacol. 25 (1), 85–88. 10.1093/ijnp/pyab078 34791262 PMC8756081

[B153] Jin K. Lu J. Yu Z. Shen Z. Li H. Mou T. (2020). Linking peripheral Il-6, Il-1beta and Hypocretin-1 with cognitive impairment from major depression. J. Affect Disord. 277, 204–211. 10.1016/j.jad.2020.08.024 32829196

[B154] Johren O. Bruggemann N. Dendorfer A. Dominiak P. (2003). Gonadal steroids differentially regulate the messenger ribonucleic acid expression of pituitary orexin type 1 receptors and adrenal orexin type 2 receptors. Endocrinology 144 (4), 1219–1225. 10.1210/en.2002-0030 12639903

[B155] Kacinski M. Budziszewska B. Lason W. Zajac A. Skowronek-Bala B. Leskiewicz M. (2012). Level of S100b protein, neuron specific enolase, orexin a, adiponectin and insulin-like growth factor in serum of pediatric patients suffering from sleep disorders with or without epilepsy. Pharmacol. Rep. 64 (6), 1427–1433. 10.1016/s1734-1140(12)70940-4 23406753

[B156] Kagerer S. M. Johren O. (2010). Interactions of orexins/hypocretins with adrenocortical functions. Acta Physiol. (Oxf) 198 (3), 361–371. 10.1111/j.1748-1716.2009.02034.x 19719797

[B157] Kaminski T. Smolinska N. (2012). Expression of orexin receptors in the pituitary. Vitam. Horm. 89, 61–73. 10.1016/B978-0-12-394623-2.00004-4 22640608

[B158] Kanbayashi T. Shimohata T. Nakashima I. Yaguchi H. Yabe I. Nishizawa M. (2009). Symptomatic narcolepsy in patients with neuromyelitis optica and multiple sclerosis: new neurochemical and immunological implications. Arch. Neurol. 66 (12), 1563–1566. 10.1001/archneurol.2009.264 20008665

[B159] Karlsson M. Zhang C. Mear L. Zhong W. Digre A. Katona B. (2021). A single-cell type transcriptomics map of human tissues. Sci. Adv. 7 (31), eabh2169. 10.1126/sciadv.abh2169 34321199 PMC8318366

[B160] Karppa M. Yardley J. Pinner K. Filippov G. Zammit G. Moline M. (2020). Long-term efficacy and tolerability of lemborexant compared with placebo in adults with insomnia disorder: results from the phase 3 randomized clinical trial sunrise 2. Sleep 43 (9), zsaa123. 10.1093/sleep/zsaa123 32585700 PMC7487867

[B161] Karteris E. Randeva H. S. Grammatopoulos D. K. Jaffe R. B. Hillhouse E. W. (2001). Expression and coupling characteristics of the crh and orexin type 2 receptors in human fetal adrenals. J. Clin. Endocrinol. Metab. 86 (9), 4512–4519. 10.1210/jcem.86.9.7849 11549701

[B162] Karteris E. Chen J. Randeva H. S. (2004). Expression of human prepro-orexin and signaling characteristics of orexin receptors in the Male reproductive system. J. Clin. Endocrinol. Metab. 89 (4), 1957–1962. 10.1210/jc.2003-031778 15070969

[B163] Kastin A. J. Akerstrom V. (1999). Orexin a but not orexin B rapidly enters brain from blood by simple diffusion. J. Pharmacol. Exp. Ther. 289 (1), 219–223. 10.1016/s0022-3565(24)38126-1 10087007

[B164] Kawada Y. Ueno S. Asayama K. Tsutsui M. Utsunomiya K. Toyohira Y. (2003). Stimulation of catecholamine synthesis by Orexin-a in bovine adrenal medullary cells through orexin receptor 1. Biochem. Pharmacol. 66 (1), 141–147. 10.1016/s0006-2952(03)00236-3 12818374

[B165] Kawada Y. Hayashibe H. Asayama K. Dobashi K. Kodera K. Uchida N. (2004). Plasma levels of Orexin-a and leptin in obese children. Clin. Pediatr. Endocrinol. 13 (1), 47–53. 10.1297/cpe.13.47 24790297 PMC4004913

[B166] Khan W. I. Ghia J. E. (2010). Gut hormones: emerging role in immune activation and inflammation. Clin. Exp. Immunol. 161 (1), 19–27. 10.1111/j.1365-2249.2010.04150.x 20408856 PMC2940144

[B167] Kim W. J. Kim H. S. (2024). Emerging and upcoming therapies in insomnia. Transl. Clin. Pharmacol. 32 (1), 1–17. 10.12793/tcp.2024.32.e5 38586124 PMC10990727

[B168] Kim H. Y. Hong E. Kim J. I. Lee W. (2004). Solution structure of human Orexin-A: regulator of appetite and wakefulness. J. Biochem. Mol. Biol. 37 (5), 565–573. 10.5483/bmbrep.2004.37.5.565 15479620

[B169] Kirchgessner A. L. (2002). Orexins in the brain-gut axis. Endocr. Rev. 23 (1), 1–15. 10.1210/edrv.23.1.0454 11844742

[B170] Kishi T. Ikuta T. Citrome L. Sakuma K. Hatano M. Hamanaka S. (2025). Comparative efficacy and safety of daridorexant, lemborexant, and suvorexant for insomnia: a systematic review and network meta-analysis. Transl. Psychiatry 15 (1), 211. 10.1038/s41398-025-03439-8 40555730 PMC12187915

[B171] Kitamura E. Hamada J. Kanazawa N. Yonekura J. Masuda R. Sakai F. (2010). The effect of Orexin-a on the pathological mechanism in the rat focal Cerebral ischemia. Neurosci. Res. 68 (2), 154–157. 10.1016/j.neures.2010.06.010 20600373

[B172] Kobylinska L. Ghita M. A. Caruntu C. Gabreanu G. Tataru C. P. Badescu S. V. (2017). Preliminary insights in oxytocin association with the onset of diabetic neuropathy. Acta Endocrinol. (Buchar) 13 (2), 249–253. 10.4183/aeb.2017.249 31149183 PMC6516456

[B173] Kohler A. Julke E. M. Stichel J. Beck-Sickinger A. G. (2024). Comparison of protocols to test peptide stability in blood plasma and cell culture supernatants. ACS Pharmacol. Transl. Sci. 7 (11), 3618–3625. 10.1021/acsptsci.4c00503 39539263 PMC11555501

[B174] Kok S. W. Roelfsema F. Overeem S. Lammers G. J. Strijers R. L. Frolich M. (2002). Dynamics of the pituitary-adrenal ensemble in hypocretin-deficient narcoleptic humans: blunted basal adrenocorticotropin release and evidence for normal time-keeping by the master pacemaker. J. Clin. Endocrinol. Metab. 87 (11), 5085–5091. 10.1210/jc.2002-020638 12414876

[B175] Kok S. W. Roelfsema F. Overeem S. Lammers G. J. Frolich M. Meinders A. E. (2004). Pulsatile Lh release is diminished, whereas fsh secretion is normal, in hypocretin-deficient narcoleptic men. Am. J. Physiol. Endocrinol. Metab. 287 (4), E630–E636. 10.1152/ajpendo.00060.2004 15172887

[B176] Kolodziejski P. A. Pruszynska-Oszmalek E. Korek E. Sassek M. Szczepankiewicz D. Kaczmarek P. (2018). Serum levels of spexin and kisspeptin negatively correlate with obesity and insulin resistance in women. Physiol. Res. 67 (1), 45–56. 10.33549/physiolres.933467 29137471

[B177] Korim W. S. Bou Farah L. McMullan S. Verberne A. J. (2014). Orexinergic activation of medullary premotor neurons modulates the adrenal sympathoexcitation to hypothalamic glucoprivation. Diabetes 63 (6), 1895–1906. 10.2337/db13-1073 24550189

[B178] Kucukali C. I. Haytural H. Benbir G. Coban A. Ulusoy C. Giris M. (2014). Reduced serum Orexin-a levels in autoimmune encephalitis and neuromyelitis optica patients. J. Neurol. Sci. 346 (1-2), 353–355. 10.1016/j.jns.2014.08.041 25218417

[B179] Kukkonen J. P. Leonard C. S. (2014). Orexin/hypocretin receptor signalling cascades. Br. J. Pharmacol. 171 (2), 314–331. 10.1111/bph.12324 23902572 PMC3904254

[B180] Kukkonen J. P. Turunen P. M. (2021). Cellular signaling mechanisms of hypocretin/orexin. Front. Neurol. Neurosci. 45, 91–102. 10.1159/000514962 34052812

[B181] Kukkonen J. P. Jacobson L. H. Hoyer D. Rinne M. K. Borgland S. L. (2024). International union of basic and clinical pharmacology cxiv: orexin receptor function, nomenclature and pharmacology. Pharmacol. Rev. 76 (5), 625–688. 10.1124/pharmrev.123.000953 38902035

[B182] Kuru M. Ueta Y. Serino R. Nakazato M. Yamamoto Y. Shibuya I. (2000). Centrally administered orexin/hypocretin activates hpa axis in rats. Neuroreport 11 (9), 1977–1980. 10.1097/00001756-200006260-00034 10884055

[B183] Lai K. Y. Li C. J. Tsai C. S. Chou W. J. Huang W. T. You H. L. (2024). Appetite hormones, neuropsychological function and methylphenidate use in children with attention-deficit/hyperactivity disorder. Psychoneuroendocrinology 170, 107169. 10.1016/j.psyneuen.2024.107169 39226626

[B184] Lanuza F. Reyes M. Blanco E. Burrows R. Peirano P. Algarin C. (2022). Association of fasting Orexin-a levels with energy intake at breakfast and subsequent snack in Chilean adolescents. Psychoneuroendocrinology 138, 105679. 10.1016/j.psyneuen.2022.105679 35182924

[B185] Larsson K. P. Peltonen H. M. Bart G. Louhivuori L. M. Penttonen A. Antikainen M. (2005). Orexin-a-induced Ca2+ entry: evidence for involvement of trpc channels and protein kinase C regulation. J. Biol. Chem. 280 (3), 1771–1781. 10.1074/jbc.M406073200 15537648

[B186] Lawrence A. J. (2017). Behavioral Neuroscience of Orexin/Hypocretin.

[B187] Lee J. H. Bang E. Chae K. J. Kim J. Y. Lee D. W. Lee W. (1999). Solution structure of a new hypothalamic neuropeptide, human hypocretin-2/Orexin-B. Eur. J. Biochem. 266 (3), 831–839. 10.1046/j.1432-1327.1999.00911.x 10583376

[B188] Lee W. C. Chen P. Y. Kao C. F. Huang M. C. (2021). Differences in serum Orexin-a levels between the acute and subacute withdrawal phases in individuals who use methamphetamine. Exp. Clin. Psychopharmacol. 29 (6), 573–579. 10.1037/pha0000395 32757597

[B189] Lehner K. R. Silverman H. A. Addorisio M. E. Roy A. Al-Onaizi M. A. Levine Y. (2019). Forebrain cholinergic signaling regulates innate immune responses and inflammation. Front. Immunol. 10, 585. 10.3389/fimmu.2019.00585 31024522 PMC6455130

[B190] Lengvenyte A. Cognasse F. Hamzeh-Cognasse H. Seneque M. Strumila R. Olie E. (2024). Baseline circulating biomarkers, their changes, and subsequent suicidal ideation and depression severity at 6 months: a prospective analysis in patients with mood disorders. Psychoneuroendocrinology 168, 107119. 10.1016/j.psyneuen.2024.107119 39003840

[B191] Li A. Nattie E. (2014). Orexin, cardio-respiratory function, and hypertension. Front. Neurosci. 8, 22. 10.3389/fnins.2014.00022 24574958 PMC3921571

[B192] Li T. Xu W. Ouyang J. Lu X. Sherchan P. Lenahan C. (2020). Orexin a alleviates neuroinflammation *via* Oxr2/Camkkbeta/Ampk signaling pathway after Ich in mice. J. Neuroinflammation 17 (1), 187. 10.1186/s12974-020-01841-1 32539736 PMC7294616

[B193] Li H. Lu J. Li S. Huang B. Shi G. Mou T. (2021). Increased hypocretin (Orexin) plasma level in depression, bipolar disorder patients. Front. Psychiatry 12, 676336. 10.3389/fpsyt.2021.676336 34135789 PMC8200484

[B194] Li J. Wang Q. Qian J. Chen X. Li D. Song C. (2023). Correlation of serum Orexin-a level with cognitive function and serum inflammatory cytokines in epileptic patients. Am. J. Transl. Res. 15 (6), 4110–4117. 37434836 PMC10331673

[B195] Liao X. M. Yu Q. (2005). The changes and implications of plasma Orexin-a levels in patients with obstructive sleep apnea-hypopnea syndrome. Zhonghua Jie He He Hu Xi Za Zhi 28 (6), 368–371. 10.3760/cma.j.issn.1001-0939.2005.06.104 16008971

[B196] Liu Y. Zhao Y. Ju S. Guo L. (2015). Orexin a upregulates the protein expression of Ox1r and enhances the proliferation of Sgc-7901 gastric cancer cells through the erk signaling pathway. Int. J. Mol. Med. 35 (2), 539–545. 10.3892/ijmm.2014.2038 25515760

[B197] Liu Y. Zhao Y. Guo L. (2016). Effects of orexin a on glucose metabolism in human hepatocellular carcinoma *in vitro via* Pi3k/Akt/Mtor-Dependent and -independent mechanism. Mol. Cell Endocrinol. 420, 208–216. 10.1016/j.mce.2015.11.002 26549689

[B198] Liu Z. Zhang Y. Zhao T. Wang J. Xia L. Zhong Y. (2020). A higher body mass index in Chinese inpatients with chronic schizophrenia is associated with elevated plasma Orexin-a levels and fewer negative symptoms. Nord. J. Psychiatry 74 (7), 525–532. 10.1080/08039488.2020.1755995 32363986

[B199] Lopez M. Senaris R. Gallego R. Garcia-Caballero T. Lago F. Seoane L. (1999). Orexin receptors are expressed in the adrenal medulla of the rat. Endocrinology 140 (12), 5991–5994. 10.1210/endo.140.12.7287 10579367

[B200] Lopez M. Seoane L. Senaris R. M. Dieguez C. (2001). Prepro-Orexin mrna levels in the rat hypothalamus, and orexin receptors mrna levels in the rat hypothalamus and adrenal gland are not influenced by the thyroid status. Neurosci. Lett. 300 (3), 171–175. 10.1016/s0304-3940(01)01569-5 11226638

[B201] Lu J. Huang M. L. Li J. H. Jin K. Y. Li H. M. Mou T. T. (2021). Changes of hypocretin (Orexin) system in schizophrenia: from plasma to brain. Schizophr. Bull. 47 (5), 1310–1319. 10.1093/schbul/sbab042 33974073 PMC8379539

[B202] Lucey B. P. Hicks T. J. McLeland J. S. Toedebusch C. D. Boyd J. Elbert D. L. (2018). Effect of sleep on overnight cerebrospinal fluid amyloid beta kinetics. Ann. Neurol. 83 (1), 197–204. 10.1002/ana.25117 29220873 PMC5876097

[B203] Lucey B. P. Liu H. Toedebusch C. D. Freund D. Redrick T. Chahin S. L. (2023). Suvorexant acutely decreases tau phosphorylation and abeta in the human cns. Ann. Neurol. 94 (1), 27–40. 10.1002/ana.26641 36897120 PMC10330114

[B204] Majmudar D. East P. Martinez S. Blanco E. Lozoff B. Burrows R. (2022). Associations between adverse home environments and appetite hormones, adipokines, and adiposity among Chilean adolescents. Clin. Obes. 12 (1), e12488. 10.1111/cob.12488 34569164

[B205] Makela K. A. Karhu T. Jurado Acosta A. Vakkuri O. Leppaluoto J. Herzig K. H. (2018). Plasma Orexin-a levels do not undergo circadian rhythm in young healthy Male subjects. Front. Endocrinol. (Lausanne) 9, 710. 10.3389/fendo.2018.00710 30568633 PMC6289979

[B206] Malendowicz L. K. Tortorella C. Nussdorfer G. G. (1999). Orexins stimulate corticosterone secretion of rat adrenocortical cells, through the activation of the adenylate cyclase-dependent signaling Cascade. J. Steroid Biochem. Mol. Biol. 70 (4-6), 185–188. 10.1016/s0960-0760(99)00110-7 10622406

[B207] Malendowicz L. K. Hochol A. Ziolkowska A. Nowak M. Gottardo L. Nussdorfer G. G. (2001a). Prolonged orexin administration stimulates steroid-hormone secretion, acting directly on the rat adrenal gland. Int. J. Mol. Med. 7 (4), 401–404. 10.3892/ijmm.7.4.401 11254881

[B208] Malendowicz L. K. Jedrzejczak N. Belloni A. S. Trejter M. Hochól A. Nussdorfer G. G. (2001b). Effects of orexins a and B on the secretory and proliferative activity of immature and regenerating rat adrenal glands. Histol. Histopathol. 16 (3), 713–717. 10.14670/HH-16.713 11510960

[B209] Malendowicz W. Szyszka M. Ziolkowska A. Rucinski M. Kwias Z. (2011). Elevated expression of orexin receptor 2 (Hcrtr2) in benign prostatic hyperplasia is accompanied by lowered serum orexin a concentrations. Int. J. Mol. Med. 27 (3), 377–383. 10.3892/ijmm.2010.590 21186399

[B210] Manzardo A. M. Johnson L. Miller J. L. Driscoll D. J. Butler M. G. (2016). Higher plasma orexin a levels in children with Prader-Willi syndrome compared with healthy unrelated sibling controls. Am. J. Med. Genet. A 170 (8), 2097–2102. 10.1002/ajmg.a.37749 27214028

[B211] Martelli D. McKinley M. J. McAllen R. M. (2014). The cholinergic anti-inflammatory pathway: a critical review. Auton. Neurosci. 182, 65–69. 10.1016/j.autneu.2013.12.007 24411268

[B212] Mashimo M. Kawashima K. Fujii T. (2022). Non-neuronal cholinergic muscarinic acetylcholine receptors in the regulation of immune function. Biol. Pharm. Bull. 45 (6), 675–683. 10.1248/bpb.b21-01005 35650095

[B213] Mazzocchi G. Malendowicz L. K. Gottardo L. Aragona F. Nussdorfer G. G. (2001a). Orexin a stimulates cortisol secretion from human adrenocortical cells through activation of the adenylate cyclase-dependent signaling Cascade. J. Clin. Endocrinol. Metab. 86 (2), 778–782. 10.1210/jcem.86.2.7233 11158046

[B214] Mazzocchi G. Malendowicz L. K. Aragona F. Rebuffat P. Gottardo L. Nussdorfer G. G. (2001b). Human Pheochromocytomas express orexin receptor type 2 gene and display an *in vitro* secretory response to orexins a and B. J. Clin. Endocrinol. Metab. 86 (10), 4818–4821. 10.1210/jcem.86.10.7929 11600547

[B215] McElroy S. L. Coloma P. M. Berger B. Guerdjikova A. I. Joyce J. M. Liebowitz M. R. (2023). Efficacy, safety, and tolerability of nivasorexant in adults with binge-eating disorder: a randomized, phase Ii proof of concept trial. Int. J. Eat. Disord. 56 (11), 2120–2130. 10.1002/eat.24039 37584285

[B216] Merck. Belsomra dosing administration. (2025). Available online at: https://www.merckconnect.com/belsomra/dosing-administration/ (Accesed April 4, 2025).

[B217] Mesens S. Krystal A. D. Melkote R. Xu H. Pandina G. Saoud J. B. (2025a). Efficacy and safety of seltorexant in insomnia disorder: a randomized clinical trial. JAMA Psychiatry 82 (10), 967–976. 10.1001/jamapsychiatry.2025.1999 40802194 PMC12351464

[B218] Mesens S. Kezic I. Van Der Ark P. Etropolski M. Pandina G. Benes H. (2025b). Treatment effect and safety of seltorexant as monotherapy for patients with major depressive disorder: a randomized, placebo-controlled clinical trial. Mol. Psychiatry 30 (6), 2427–2435. 10.1038/s41380-024-02846-5 39663378 PMC12092288

[B219] Messal N. Fernandez N. Dayot S. Gratio V. Nicole P. Prochasson C. (2018). Ectopic expression of Ox1r in ulcerative colitis mediates anti-inflammatory effect of Orexin-A. Biochim. Biophys. Acta Mol. Basis Dis. 1864 (11), 3618–3628. 10.1016/j.bbadis.2018.08.023 30251681

[B220] Messina A. De Fusco C. Monda V. Esposito M. Moscatelli F. Valenzano A. (2016a). Role of the orexin system on the hypothalamus-pituitary-thyroid axis. Front. Neural Circuits 10, 66. 10.3389/fncir.2016.00066 27610076 PMC4997012

[B221] Messina G. Di Bernardo G. Viggiano A. De Luca V. Monda V. Messina A. (2016b). Exercise increases the level of plasma orexin a in humans. J. Basic Clin. Physiol. Pharmacol. 27 (6), 611–616. 10.1515/jbcpp-2015-0133 27665420

[B222] Meusel M. Voss J. Krapalis A. Machleidt F. Vonthein R. Hallschmid M. (2022). Intranasal orexin a modulates sympathetic vascular tone: a pilot study in healthy Male humans. J. Neurophysiol. 127 (2), 548–558. 10.1152/jn.00452.2021 35044844

[B223] Mignot E. Mayleben D. Fietze I. Leger D. Zammit G. Bassetti C. L. A. (2022). Safety and efficacy of daridorexant in patients with insomnia disorder: results from two multicentre, randomised, double-blind, placebo-controlled, phase 3 trials. Lancet Neurol. 21 (2), 125–139. 10.1016/S1474-4422(21)00436-1 35065036

[B224] Mishra S. Gupta V. Mishra S. Sachan R. Asthana A. (2017). Serum level of Orexin-a, leptin, adiponectin and insulin in north Indian Obese women. Diabetes Metab. Syndr. 11 (Suppl. 2), S1041–S1043. 10.1016/j.dsx.2017.07.037 28755843

[B225] Mitsukawa K. Kimura H. (2022). Orexin 2 receptor (Ox2r) protein distribution measured by autoradiography using radiolabeled Ox2r-Selective antagonist empa in rodent brain and peripheral tissues. Sci. Rep. 12 (1), 8473. 10.1038/s41598-022-12601-x 35589803 PMC9120030

[B226] Modi H. R. Wang Q. Gd S. Sherman D. Greenwald E. Savonenko A. V. (2017). Intranasal post-cardiac arrest treatment with Orexin-a facilitates arousal from coma and ameliorates neuroinflammation. PLoS One 12 (9), e0182707. 10.1371/journal.pone.0182707 28957432 PMC5619710

[B227] Mohamed A. R. El-Hadidy W. F. (2014). Effect of Orexin-a (Hypocretin-1) on hyperalgesic and cachectic manifestations of experimentally induced rheumatoid arthritis in rats. Can. J. Physiol. Pharmacol. 92 (10), 813–820. 10.1139/cjpp-2014-0258 25211278

[B228] Moharami S. Nourazarian A. Nikanfar M. Laghousi D. Shademan B. Joodi Khanghah O. (2022). Investigation of serum levels of Orexin-a, transforming growth factor beta, and leptin in patients with multiple sclerosis. J. Clin. Lab. Anal. 36 (1), e24170. 10.1002/jcla.24170 34894407 PMC8761413

[B229] Monda V. Carotenuto M. Precenzano F. Iacono D. Messina A. Salerno M. (2020a). Neuropeptides' hypothalamic regulation of sleep control in children affected by functional non-retentive fecal incontinence. Brain Sci. 10 (3), 129. 10.3390/brainsci10030129 32106434 PMC7139357

[B230] Monda V. Sessa F. Ruberto M. Carotenuto M. Marsala G. Monda M. (2020b). Aerobic exercise and metabolic syndrome: the role of sympathetic activity and the redox system. Diabetes Metab. Syndr. Obes. 13, 2433–2442. 10.2147/DMSO.S257687 32753926 PMC7354914

[B231] Moreno G. Perello M. Gaillard R. C. Spinedi E. (2005). Orexin a stimulates hypothalamic-pituitary-adrenal (Hpa) axis function, but not food intake, in the absence of full hypothalamic Npy-Ergic activity. Endocrine 26 (2), 99–106. 10.1385/ENDO:26:2:099 15888921

[B232] Mostefai H. A. Agouni A. Carusio N. Mastronardi M. L. Heymes C. Henrion D. (2008). Phosphatidylinositol 3-kinase and xanthine oxidase regulate nitric oxide and reactive oxygen species productions by apoptotic lymphocyte microparticles in endothelial cells. J. Immunol. 180 (7), 5028–5035. 10.4049/jimmunol.180.7.5028 18354228

[B233] Muehlan C. Vaillant C. Zenklusen I. Kraehenbuehl S. Dingemanse J. (2020). Clinical pharmacology, efficacy, and safety of orexin receptor antagonists for the treatment of insomnia disorders. Expert Opin. Drug Metab. Toxicol. 16 (11), 1063–1078. 10.1080/17425255.2020.1817380 32901578

[B234] Murakami M. Yamamoto K. Miki Y. Murase R. Sato H. Taketomi Y. (2016). The roles of the secreted phospholipase a(2) gene family in immunology. Adv. Immunol. 132, 91–134. 10.1016/bs.ai.2016.05.001 27769509 PMC7112020

[B235] Nakabayashi M. Suzuki T. Takahashi K. Totsune K. Muramatsu Y. Kaneko C. (2003). Orexin-a expression in human peripheral tissues. Mol. Cell Endocrinol. 205 (1-2), 43–50. 10.1016/s0303-7207(03)00206-5 12890566

[B236] Nakashima H. Umegaki H. Yanagawa M. Komiya H. Watanabe K. Kuzuya M. (2019a). Plasma Orexin-a-like immunoreactivity levels and renal function in patients in a geriatric ward. Peptides 118, 170092. 10.1016/j.peptides.2019.170092 31163198

[B237] Nakashima H. Umegaki H. Yanagawa M. Komiya H. Watanabe K. Kuzuya M. (2019b). Plasma Orexin-a levels in patients with delirium. Psychogeriatrics 19 (6), 628–630. 10.1111/psyg.12444 30884029 PMC6899688

[B238] Nanmoku T. Isobe K. Sakurai T. Yamanaka A. Takekoshi K. Kawakami Y. (2000). Orexins suppress catecholamine synthesis and secretion in cultured Pc12 cells. Biochem. Biophys. Res. Commun. 274 (2), 310–315. 10.1006/bbrc.2000.3137 10913336

[B239] Nanmoku T. Isobe K. Sakurai T. Yamanaka A. Takekoshi K. Kawakami Y. (2002). Effects of orexin on cultured porcine adrenal medullary and cortex cells. Regul. Pept. 104 (1-3), 125–130. 10.1016/s0167-0115(01)00356-1 11830287

[B240] Nasman J. Bart G. Larsson K. Louhivuori L. Peltonen H. Akerman K. E. (2006). The orexin Ox1 receptor regulates Ca2+ entry *via* diacylglycerol-activated channels in differentiated neuroblastoma cells. J. Neurosci. 26 (42), 10658–10666. 10.1523/JNEUROSCI.2609-06.2006 17050705 PMC6674737

[B241] Neumann K. Tiegs G. (2021). Immune regulation in renal inflammation. Cell Tissue Res. 385 (2), 305–322. 10.1007/s00441-020-03351-1 33496881 PMC8523435

[B242] Nigro E. D'Arco D. Moscatelli F. Pisani A. Amicone M. Riccio E. (2024). Increased expression of Orexin-a in patients affected by polycystic kidney disease. Int. J. Mol. Sci. 25 (11), 6243. 10.3390/ijms25116243 38892431 PMC11172798

[B243] Nishijima T. Sakurai S. Arihara Z. Takahashi K. (2003). Plasma Orexin-a-like immunoreactivity in patients with sleep apnea hypopnea syndrome. Peptides 24 (3), 407–411. 10.1016/s0196-9781(03)00055-x 12732338

[B244] Nowak M. Hochol A. Tortorella C. Jedrzejczak N. Ziolkowska A. Nussdorfer G. G. (2000). Modulatory effects of orexins on the function of Rat pituitary-adrenocortical axis under basal and stressful conditions. Biomed. Res-Tokyo 21 (2), 89–93. 10.2220/biomedres.21.89

[B245] Ogawa Y. Irukayama-Tomobe Y. Murakoshi N. Kiyama M. Ishikawa Y. Hosokawa N. (2016). Peripherally administered orexin improves survival of mice with endotoxin shock. Elife 5, 5. 10.7554/eLife.21055 28035899 PMC5245965

[B246] Ogawa Y. Ezaki S. Shimojo N. Kawano S. (2021). Case report: reduced csf orexin levels in a patient with sepsis. Front. Neurosci. 15, 739323. 10.3389/fnins.2021.739323 34690677 PMC8526783

[B247] Ogawa Y. Shimojo N. Ishii A. Tamaoka A. Kawano S. Inoue Y. (2022). Reduced csf orexin levels in rats and patients with systemic inflammation: a preliminary study. BMC Res. Notes 15 (1), 221. 10.1186/s13104-022-06121-0 35752867 PMC9233848

[B248] Ohi Y. Asai Y. Kodama D. (2022). Distinct effects of orexin a on spontaneous and evoked synaptic currents in the rat nucleus tractus solitarius. J. Pharmacol. Sci. 150 (4), 244–250. 10.1016/j.jphs.2022.09.008 36344046

[B249] Olofsson P. S. Rosas-Ballina M. Levine Y. A. Tracey K. J. (2012). Rethinking inflammation: neural circuits in the regulation of immunity. Immunol. Rev. 248 (1), 188–204. 10.1111/j.1600-065X.2012.01138.x 22725962 PMC4536565

[B250] Orum D. Korkmaz S. Ilhan N. Orum M. H. Atmaca M. (2024). Leptin, Nesfatin-1, Orexin-a, and total ghrelin levels in drug-naive panic disorder. Psychiatry Investig. 21 (2), 142–150. 10.30773/pi.2023.0309 38433413 PMC10910167

[B251] Ozkanli S. Basar M. M. Selimoglu S. Erol B. Ozkanli O. Nurili F. (2018). The ghrelin and orexin activity in testicular tissues of patients with idiopathic non-obstructive azoospermia. Kaohsiung J. Med. Sci. 34 (10), 564–568. 10.1016/j.kjms.2018.04.001 30309484 PMC11915662

[B252] Ozsoy S. Olguner E. O. Abdulrezzak U. Esel E. (2017). Relationship between orexin a and childhood maltreatment in female patients with depression and anxiety. Soc. Neurosci. 12 (3), 330–336. 10.1080/17470919.2016.1169216 27043067

[B253] Ozturk O. Cebeci D. Sahin U. Tuluceoglu E. E. Calapoglu N. S. Gonca T. (2022). Circulating levels of ghrelin, galanin, and Orexin-a orexigenic neuropeptides in obstructive sleep apnea syndrome. Sleep. Breath. 26 (3), 1209–1218. 10.1007/s11325-021-02514-w 34689311

[B254] Pan J. Chen G. Shan P. Chen C. Jiang D. Wang L. (2021). Plasma orexin levels related to altered brain activity during abstinence in patients with alcohol dependence. Psychiatry Clin. Psychopharmacol. 31 (3), 286–291. 10.5152/pcp.2021.20011 38765947 PMC11079708

[B255] Parenti A. De Logu F. Geppetti P. Benemei S. (2016). What is the evidence for the role of trp channels in inflammatory and immune cells? Br. J. Pharmacol 173 (6), 953–969. 10.1111/bph.13392 26603538 PMC5341240

[B256] Parhizkar S. Bao X. Chen W. Rensing N. Chen Y. Kipnis M. (2025). Lemborexant ameliorates tau-mediated sleep loss and neurodegeneration in males in a mouse model of tauopathy. Nat. Neurosci. 28 (7), 1460–1472. 10.1038/s41593-025-01966-7 40425791 PMC12277058

[B257] Park J. H. Shim H. M. Na A. Y. Bae J. H. Im S. S. Song D. K. (2015). Orexin a regulates plasma insulin and Leptin levels in a time-dependent manner following a glucose load in mice. Diabetologia 58 (7), 1542–1550. 10.1007/s00125-015-3573-0 25813215

[B258] Park T. H. Lee H. J. Kwon R. W. Lee I. H. Lee S. J. Park J. I. (2022). Effects of caffeine ingestion and thermotherapy on blood orexin circulation in humans. Food Sci. Biotechnol. 31 (9), 1207–1212. 10.1007/s10068-022-01094-z 35615306 PMC9122480

[B259] Patel V. H. Karteris E. Chen J. Kyrou I. Mattu H. S. Dimitriadis G. K. (2018). Functional Cardiac orexin receptors: Role of Orexin-B/Orexin 2 receptor in myocardial protection. Clin. Sci. (Lond) 132 (24), 2547–2564. 10.1042/CS20180150 30467191 PMC6365625

[B260] Pavlov V. A. Wang H. Czura C. J. Friedman S. G. Tracey K. J. (2003). The cholinergic anti-inflammatory pathway: a missing link in neuroimmunomodulation. Mol. Med. 9 (5-8), 125–134. 14571320 PMC1430829

[B261] Pongratz G. Straub R. H. (2014). The sympathetic nervous response in inflammation. Arthritis Res. Ther. 16 (6), 504. 10.1186/s13075-014-0504-2 25789375 PMC4396833

[B262] Populin L. Stebbing M. J. Furness J. B. (2021). Neuronal regulation of the gut immune system and neuromodulation for treating inflammatory bowel disease. FASEB Bioadv 3 (11), 953–966. 10.1096/fba.2021-00070 34761177 PMC8565205

[B263] Qin L. Luo Y. Chang H. Zhang H. Zhu Z. Du Y. (2023). The association between serum Orexin-a levels and sleep quality in pregnant women. Sleep. Med. 101, 93–98. 10.1016/j.sleep.2022.10.019 36368074

[B264] Rahaman O. Ganguly D. (2021). Endocannabinoids in immune regulation and immunopathologies. Immunology 164 (2), 242–252. 10.1111/imm.13378 34053085 PMC8442232

[B265] Raich I. Rebassa J. B. Lillo J. Cordomi A. Rivas-Santisteban R. Lillo A. (2022). Antagonization of Ox(1) receptor potentiates Cb(2) receptor function in microglia from App(Sw/Ind) mice model. Int. J. Mol. Sci. 23 (21), 12801. 10.3390/ijms232112801 36361598 PMC9656664

[B266] Ramanjaneya M. Conner A. C. Chen J. Stanfield P. R. Randeva H. S. (2008). Orexins stimulate steroidogenic acute regulatory protein expression through multiple signaling pathways in human adrenal H295r cells. Endocrinology 149 (8), 4106–4115. 10.1210/en.2007-1739 18450961 PMC2488249

[B267] Ramanjaneya M. Conner A. C. Chen J. Kumar P. Brown J. E. Johren O. (2009). Orexin-stimulated map kinase cascades are activated through multiple G-Protein signalling pathways in human H295r adrenocortical cells: diverse roles for orexins a and B. J. Endocrinol. 202 (2), 249–261. 10.1677/JOE-08-0536 19460850

[B268] Randeva H. S. Karteris E. Grammatopoulos D. Hillhouse E. W. (2001). Expression of Orexin-a and functional orexin type 2 receptors in the human adult adrenals: implications for adrenal function and energy homeostasis. J. Clin. Endocrinol. Metab. 86 (10), 4808–4813. 10.1210/jcem.86.10.7921 11600545

[B269] Razavinia F. Tehranian N. Tatari F. T. Bidhendi Yarandi R. Ramezani Tehrani F. (2019). The postpartum marital satisfaction, maternal serum concentration of Orexin-a and mode of delivery. J. Sex. Marital Ther. 45 (6), 488–496. 10.1080/0092623X.2019.1566947 30640582

[B270] Razavinia F. Tehranian N. Sadatmahalleh S. J. Kazemnejad A. Khajetash S. Daryasari S. R. F. (2021). The influence of mode of delivery, anthropometric indices, and infant’s sex on the maternal and cord blood Orexin-a levels: a cohort study. J. Obstet. Gynaecol. Res. 47 (7), 2363–2370. 10.1111/jog.14758 33870593

[B271] Ren J. Chen Y. Fang X. Wang D. Wang Y. Yu L. (2022). Correlation of Orexin-a and brain-derived neurotrophic factor levels in metabolic syndrome and cognitive impairment in schizophrenia treated with clozapine. Neurosci. Lett. 782, 136695. 10.1016/j.neulet.2022.136695 35618081

[B272] Riemann D. Baglioni C. Bassetti C. Bjorvatn B. Dolenc Groselj L. Ellis J. G. (2017). European guideline for the diagnosis and treatment of insomnia. J. Sleep. Res. 26 (6), 675–700. 10.1111/jsr.12594 28875581

[B273] Rouet-Benzineb P. Rouyer-Fessard C. Jarry A. Avondo V. Pouzet C. Yanagisawa M. (2004). Orexins acting at native Ox(1) receptor in Colon cancer and neuroblastoma cells or at recombinant Ox(1) receptor suppress cell growth by inducing apoptosis. J. Biol. Chem. 279 (44), 45875–45886. 10.1074/jbc.M404136200 15310763

[B274] Russell S. H. Small C. J. Dakin C. L. Abbott C. R. Morgan D. G. Ghatei M. A. (2001). The central effects of Orexin-a in the hypothalamic-pituitary-adrenal axis *in vivo* and *in vitro* in Male rats. J. Neuroendocrinol. 13 (6), 561–566. 10.1046/j.1365-2826.2001.00672.x 11412343

[B275] Saitoh T. Sakurai T. (2023). The present and future of synthetic orexin receptor agonists. Peptides 167, 171051. 10.1016/j.peptides.2023.171051 37422012

[B276] Sakurai T. Amemiya A. Ishii M. Matsuzaki I. Chemelli R. M. Tanaka H. (1998). Orexins and orexin receptors: a family of hypothalamic neuropeptides and G protein-coupled receptors that regulate feeding behavior. Cell 92 (4), 573–585. 10.1016/s0092-8674(00)80949-6 9491897

[B277] Sakurai S. Nishijima T. Takahashi S. Yamauchi K. Arihara Z. Takahashi K. (2005). Low plasma Orexin-a levels were improved by continuous positive airway pressure treatment in patients with severe obstructive sleep apnea-hypopnea syndrome. Chest 127 (3), 731–737. 10.1378/chest.127.3.731 15764751

[B278] Salvadore G. Bonaventure P. Shekhar A. Johnson P. L. Lord B. Shireman B. T. (2020). Translational evaluation of novel selective Orexin-1 receptor antagonist Jnj-61393215 in an experimental model for panic in rodents and humans. Transl. Psychiatry 10 (1), 308. 10.1038/s41398-020-00937-9 32895369 PMC7477545

[B279] Sanchez-de-la-Torre M. Barcelo A. Pierola J. Esquinas C. de la Pena M. Duran-Cantolla J. (2011). Plasma levels of neuropeptides and metabolic hormones, and sleepiness in obstructive sleep apnea. Respir. Med. 105 (12), 1954–1960. 10.1016/j.rmed.2011.08.014 21889324

[B280] Sandikci S. C. Gultuna S. Ozisler C. Aydin F. N. (2024). The role of orexin in fatigue and sleep quality in patients with primary Sjogren's syndrome. Z Rheumatol. 83 (Suppl. 1), 242–247. 10.1007/s00393-023-01462-y 38108866

[B281] Saruhan E. Korkmaz M. Altiparmak B. Tosun K. Kutlu G. (2022). Comparison of Orexin-a and neurofilament light chain levels in patients with relapsing-remitting multiple sclerosis: a pilot study. Ideggyogy Sz. 75 (7-08), 223–230. 10.18071/isz.75.0223 35916608

[B282] Schiller M. Ben-Shaanan T. L. Rolls A. (2021). Neuronal regulation of immunity: why, how and where? Nat. Rev. Immunol. 21 (1), 20–36. 10.1038/s41577-020-0387-1 32811994

[B283] Schmidt M. E. Moyer J. A. Kezic I. Zhou X. Samtani M. N. Bleys C. (2025). Efficacy, safety, and tolerability of Jnj-61393215 (Tebideutorexant), a selective Orexin-1 receptor antagonist, as adjunctive treatment for major depressive disorder with anxious distress: a double-blind, placebo-controlled, randomized phase 2a study. Eur. Neuropsychopharmacol. 95, 14–23. 10.1016/j.euroneuro.2025.03.007 40215570

[B284] Scott A. J. Flowers O. Rowse G. (2020). A comparative study of the nature and magnitude of problems sleeping in inflammatory bowel disease (Ibd) compared to healthy controls. Psychol. Health Med. 25 (8), 958–968. 10.1080/13548506.2019.1707240 31899953

[B285] Sherman D. L. Williams A. Gd S. Modi H. R. Wang Q. Thakor N. V. (2021). Intranasal orexin after cardiac arrest leads to increased electroencephalographic gamma activity and enhanced neurologic recovery in rats. Crit. Care Explor 3 (2), e0349. 10.1097/CCE.0000000000000349 33634267 PMC7901796

[B286] Shi Y. Feng Y. Kang J. Liu C. Li Z. Li D. (2007). Critical regulation of Cd4+ T cell survival and autoimmunity by beta-arrestin 1. Nat. Immunol. 8 (8), 817–824. 10.1038/ni1489 17618287

[B287] Shin Y. O. Lee J. B. Min Y. K. Yang H. M. (2013). Heat acclimation affects circulating levels of prostaglandin E2, Cox-2 and orexin in humans. Neurosci. Lett. 542, 17–20. 10.1016/j.neulet.2013.03.017 23523649

[B288] Silverman M. N. Pearce B. D. Biron C. A. Miller A. H. (2005). Immune modulation of the hypothalamic-pituitary-adrenal (Hpa) axis during viral infection. Viral Immunol. 18 (1), 41–78. 10.1089/vim.2005.18.41 15802953 PMC1224723

[B289] Snow A. Gozal E. Malhotra A. Tiosano D. Perlman R. Vega C. (2002). Severe hypersomnolence after pituitary/hypothalamic surgery in adolescents: clinical characteristics and potential mechanisms. Pediatrics 110 (6), e74. 10.1542/peds.110.6.e74 12456941

[B290] Soejima Y. Iwata N. Nishioka R. Honda M. Nakano Y. Yamamoto K. (2023). Interaction of orexin and bone morphogenetic proteins in steroidogenesis by human adrenocortical cells. Int. J. Mol. Sci. 24 (16), 12559. 10.3390/ijms241612559 37628739 PMC10454954

[B291] Sokolowska P. Urbanska A. Bieganska K. Wagner W. Ciszewski W. Namiecinska M. (2014). Orexins protect neuronal cell cultures against hypoxic stress: an involvement of Akt signaling. J. Mol. Neurosci. 52 (1), 48–55. 10.1007/s12031-013-0165-7 24243084 PMC3929148

[B292] Soya S. Sakurai T. (2020). Evolution of orexin neuropeptide system: structure and function. Front. Neurosci. 14, 691. 10.3389/fnins.2020.00691 32754010 PMC7365868

[B293] Spinazzi R. Rucinski M. Neri G. Malendowicz L. K. Nussdorfer G. G. (2005a). Preproorexin and orexin receptors are expressed in cortisol-secreting adrenocortical adenomas, and orexins stimulate *in vitro* cortisol secretion and growth of tumor cells. J. Clin. Endocrinol. Metab. 90 (6), 3544–3549. 10.1210/jc.2004-2385 15797953

[B294] Spinazzi R. Ziolkowska A. Neri G. Nowak M. Rebuffat P. Nussdorfer G. G. (2005b). Orexins modulate the growth of cultured rat adrenocortical cells, acting through type 1 and type 2 receptors coupled to the mapk P42/P44-and P38-dependent cascades. Int. J. Mol. Med. 15 (5), 847–852. 10.3892/ijmm.15.5.847 15806308

[B295] Stawerska R. Czkwianianc E. Matusiak A. Smyczynska J. Hilczer M. Chmiela M. (2016). Assessment of Ghrelin, Leptin, orexin a and Alpha-Msh serum concentrations and the levels of the autoantibodies against the aforementioned peptides in relation to Helicobacter Pylori infections and Candida Albicans colonization in children with short stature. Pediatr. Endocrinol. Diabetes Metab. 21 (3), 102–110. 10.18544/PEDM-21.03.0031 27275765

[B296] Steward T. Mestre-Bach G. Granero R. Sanchez I. Riesco N. Vintro-Alcaraz C. (2019). Reduced plasma Orexin-a concentrations are associated with cognitive deficits in anorexia nervosa. Sci. Rep. 9 (1), 7910. 10.1038/s41598-019-44450-6 31133733 PMC6536521

[B297] Sun G. Tian Z. Yao Y. Li H. Higuchi T. (2006). Central and/or peripheral immunoreactivity of Orexin-a in pregnant rats and women. J. Mol. Endocrinol. 36 (1), 131–138. 10.1677/jme.1.01818 16461933

[B298] Sun M. Wang W. Li Q. Yuan T. Weng W. (2018). Orexin a may suppress inflammatory response in fibroblast-like synoviocytes. Biomed. Pharmacother. 107, 763–768. 10.1016/j.biopha.2018.07.159 30142537

[B299] Suo L. Chang X. Zhao Y. (2018). The Orexin-a-regulated Akt/Mtor pathway promotes cell proliferation through inhibiting apoptosis in pancreatic cancer cells. Front. Endocrinol. (Lausanne) 9, 647. 10.3389/fendo.2018.00647 30429828 PMC6220114

[B300] Suzuki K. Miyamoto M. Miyamoto T. Matsubara T. Inoue Y. Iijima M. (2018). Cerebrospinal fluid Orexin-a levels in systemic lupus erythematosus patients presenting with excessive daytime sleepiness. Lupus 27 (11), 1847–1853. 10.1177/0961203318778767 29848165

[B301] Synchikova A. P. Korneva E. A. (2023). Morphofunctional features of microglial cells during the administration of orexin A. Bull. Exp. Biol. Med. 174 (5), 685–688. 10.1007/s10517-023-05770-w 37043064

[B302] Szlachcic A. Majka J. Strzalka M. Szmyd J. Pajdo R. Ptak-Belowska A. (2013). Experimental healing of preexisting gastric ulcers induced by hormones controlling food intake ghrelin, Orexin-a and Nesfatin-1 is impaired under diabetic conditions. A key to understanding the diabetic gastropathy? J. Physiol. Pharmacol. 64 (5), 625–637. 24304576

[B303] Szyszka M. Paschke L. Tyczewska M. Rucinski M. Grabowska P. Malendowicz L. K. (2015). Lack of expression of preproorexin and orexin receptors genes in human normal and prostate cancer cell lines. Folia Histochem Cytobiol. 53 (4), 333–341. 10.5603/fhc.a2015.0035 26714447

[B304] Takahashi K. Arihara Z. Suzuki T. Sone M. Kikuchi K. Sasano H. (2006). Expression of Orexin-a and orexin receptors in the kidney and the presence of Orexin-a-like immunoreactivity in human urine. Peptides 27 (4), 871–877. 10.1016/j.peptides.2005.08.008 16202475

[B305] Takai T. Takaya T. Nakano M. Akutsu H. Nakagawa A. Aimoto S. (2006). Orexin-a is composed of a highly conserved C-Terminal and a specific, hydrophilic N-Terminal region, revealing the structural basis of specific recognition by the Orexin-1 receptor. J. Pept. Sci. 12 (7), 443–454. 10.1002/psc.747 16429482

[B306] Takekawa D. Kushikata T. Akaishi M. Nikaido Y. Hirota K. (2019). Influence of orexinergic system on survival in septic rats. Neuropsychobiology 77 (1), 45–48. 10.1159/000493739 30326465

[B307] Tanaka A. Furubayashi T. Arai M. Inoue D. Kimura S. Kiriyama A. (2018). Delivery of Oxytocin to the brain for the treatment of autism spectrum disorder by nasal application. Mol. Pharm. 15 (3), 1105–1111. 10.1021/acs.molpharmaceut.7b00991 29338251

[B308] Tang S. Huang W. Lu S. Lu L. Li G. Chen X. (2017). Increased plasma Orexin-a levels in patients with insomnia disorder are not associated with prepro-orexin or orexin receptor gene polymorphisms. Peptides 88, 55–61. 10.1016/j.peptides.2016.12.008 27988352

[B309] Taximaimaiti R. Abuliken X. Maihemuti M. Abudujilile D. Abudulimu H. (2016). Elevated expression of Ox2r in cervical cancers and placentas of Uyghur women in Xinjiang, China. Asian Pac J. Cancer Prev. 17 (11), 4959–4963. 10.22034/APJCP.2016.17.11.4959 28032723 PMC5454703

[B310] The Human Protein Atlas. Hcrt. Available online at: https://www.proteinatlas.org/ensg00000161610-hcrt/tissue (Accesed January 2, 2026).

[B311] The Human Protein Atlas. Hcrtr2. Available online at: https://www.proteinatlas.org/ensg00000137252-hcrtr2/tissue (Accesed January 2, 2026).

[B312] The Human Protein Atlas. Hcrtr1. Available online at: https://www.proteinatlas.org/ensg00000121764-hcrtr1/tissue (Accesed January 2, 2026).

[B313] Thorpy M. J. (2020). Recently approved and upcoming treatments for Narcolepsy. CNS Drugs 34 (1), 9–27. 10.1007/s40263-019-00689-1 31953791 PMC6982634

[B314] Tohma Y. Akturk M. Altinova A. Yassibas E. Cerit E. T. Gulbahar O. (2015). Circulating levels of Orexin-a, Nesfatin-1, agouti-related peptide, and neuropeptide Y in patients with hyperthyroidism. Thyroid 25 (7), 776–783. 10.1089/thy.2014.0515 25915725

[B315] Tomasik P. J. Sztefko K. (2009). The effect of enteral and parenteral feeding on secretion of orexigenic peptides in infants. BMC Gastroenterol. 9, 92. 10.1186/1471-230X-9-92 20003268 PMC2803482

[B316] Tomasik P. J. Spodaryk M. Sztefko K. (2004). Plasma concentrations of orexins in children. Ann. Nutr. Metab. 48 (4), 215–220. 10.1159/000080453 15331880

[B317] Tsai M. C. Huang T. L. (2018). Orexin a in men with heroin use disorder undergoing methadone maintenance treatment. Psychiatry Res. 264, 412–415. 10.1016/j.psychres.2018.04.010 29680730

[B318] Tsuchimine S. Hattori K. Ota M. Hidese S. Teraishi T. Sasayama D. (2019). Reduced plasma Orexin-a levels in patients with bipolar disorder. Neuropsychiatr. Dis. Treat. 15, 2221–2230. 10.2147/NDT.S209023 31496705 PMC6689769

[B319] Tsuneki H. Murata S. Anzawa Y. Soeda Y. Tokai E. Wada T. (2008). Age-Related insulin resistance in hypothalamus and peripheral tissues of orexin knockout mice. Diabetologia 51 (4), 657–667. 10.1007/s00125-008-0929-8 18256806

[B320] Tsuneki H. Wada T. Sasaoka T. (2012). Role of orexin in the central regulation of glucose and energy homeostasis. Endocr. J. 59 (5), 365–374. 10.1507/endocrj.ej12-0030 22293586

[B321] Tunisi L. Forte N. Fernandez-Rilo A. C. Mavaro I. Capasso R. D’Angelo L. (2019). Orexin-a prevents lipopolysaccharide-induced neuroinflammation at the level of the intestinal barrier. Front. Endocrinol. (Lausanne) 10, 219. 10.3389/fendo.2019.00219 31024456 PMC6467935

[B322] Turcotte C. Blanchet M. R. Laviolette M. Flamand N. (2016). The Cb(2) receptor and its role as a regulator of inflammation. Cell Mol. Life Sci. 73 (23), 4449–4470. 10.1007/s00018-016-2300-4 27402121 PMC5075023

[B323] Turkoglu R. Benbir G. Ozyurt S. Arsoy E. Akbayir E. Turan S. (2020). Sleep disturbance and cognitive decline in multiple sclerosis patients with isolated optic neuritis as the first demyelinating event. Int. Ophthalmol. 40 (1), 151–158. 10.1007/s10792-019-01157-x 31432354

[B324] Turunen P. M. Ekholm M. E. Somerharju P. Kukkonen J. P. (2010). Arachidonic acid release mediated by Ox1 orexin receptors. Br. J. Pharmacol. 159 (1), 212–221. 10.1111/j.1476-5381.2009.00535.x 20002100 PMC2823366

[B325] Uchiyama M. Kambe D. Hasegawa S. Imadera Y. Yamasaki H. Uchimura N. (2026). Efficacy and safety of vornorexant in Japanese patients with insomnia: a randomized, placebo-controlled phase 3 pivotal study. Sleep 49 (3), zsaf291. 10.1093/sleep/zsaf291 41001841 PMC13017578

[B326] Uhlen M. Fagerberg L. Hallstrom B. M. Lindskog C. Oksvold P. Mardinoglu A. (2015). Proteomics. Tissue-based map of the human proteome. Science 347 (6220), 1260419. 10.1126/science.1260419 25613900

[B327] Uhlen M. Karlsson M. J. Zhong W. Tebani A. Pou C. Mikes J. (2019). A genome-wide transcriptomic analysis of protein-coding genes in human blood cells. Science 366 (6472), aax9198. 10.1126/science.aax9198 31857451

[B328] Unler M. Ekmekci Ertek I. Afandiyeva N. Kavutcu M. Yuksel N. (2022). The role of neuropeptide Y, Orexin-a, and ghrelin in differentiating unipolar and bipolar depression: a preliminary Study. Nord. J. Psychiatry 76 (3), 162–169. 10.1080/08039488.2022.2048887 35282777

[B329] Ustabas Kahraman F. Vehapoglu A. Ozgen I. T. Terzioglu S. Cesur Y. Dundaroz R. (2015). Correlation of brain neuropeptide (Nesfatin-1 and Orexin-a) concentrations with anthropometric and biochemical parameters in malnourished children. J. Clin. Res. Pediatr. Endocrinol. 7 (3), 197–202. 10.4274/jcrpe.1930 26831553 PMC4677554

[B330] Valiante S. Liguori G. Tafuri S. Pavone L. M. Campese R. Monaco R. (2015). Expression and potential role of the peptide Orexin-a in prostate cancer. Biochem. Biophys. Res. Commun. 464 (4), 1290–1296. 10.1016/j.bbrc.2015.07.124 26220343

[B331] Van de Bittner G. C. Van de Bittner K. C. Wey H. Y. Rowe W. Dharanipragada R. Ying X. (2018). Positron emission tomography assessment of the intranasal delivery route for orexin A. ACS Chem. Neurosci. 9 (2), 358–368. 10.1021/acschemneuro.7b00357 29035509

[B332] van Lemmen M. Dahan A. Hang Y. Jansen S. C. Lu H. Naylor M. (2025). Tak-925 (Danavorexton), an orexin receptor 2 agonist, reduces opioid-induced respiratory depression and sedation without affecting Analgesia in healthy men. Anesthesiology 142 (4), 628–638. 10.1097/ALN.0000000000005375 39804333 PMC11892998

[B333] Vilke G. M. Mash D. C. Pardo M. Bozeman W. Hall C. Sloane C. (2019). Excitation Study: unexplained in-Custody deaths: evaluating biomarkers of stress and agitation. J. Forensic Leg. Med. 66, 100–106. 10.1016/j.jflm.2019.06.009 31252195

[B334] Villano I. La Marra M. Di Maio G. Monda V. Chieffi S. Guatteo E. (2022). Physiological role of orexinergic system for health. Int. J. Environ. Res. Public Health 19 (14), 8353. 10.3390/ijerph19148353 35886210 PMC9323672

[B335] Voisin T. El Firar A. Fasseu M. Rouyer-Fessard C. Descatoire V. Walker F. (2011). Aberrant expression of Ox1 receptors for orexins in Colon cancers and liver metastases: an openable gate to apoptosis. Cancer Res. 71 (9), 3341–3351. 10.1158/0008-5472.CAN-10-3473 21415167

[B336] Voisin T. Nicole P. Gratio V. Chassac A. Mansour D. Rebours V. (2022). The Orexin-a/Ox1r system induces cell death in pancreatic cancer cells resistant to gemcitabine and nab-paclitaxel treatment. Front. Oncol. 12, 904327. 10.3389/fonc.2022.904327 35747788 PMC9209740

[B337] Wan X. Liu Y. Zhao Y. Sun X. Fan D. Guo L. (2017). Orexin a affects Hepg2 human hepatocellular carcinoma cells glucose metabolism *via* Hif-1alpha-dependent and -Independent mechanism. PLoS One 12 (9), e0184213. 10.1371/journal.pone.0184213 28886081 PMC5590901

[B338] Wan L. Xu F. Liu C. Ji B. Zhang R. Wang P. (2020). Transmembrane peptide 4 and 5 of Apj are essential for its heterodimerization with Ox1r. Biochem. Biophys. Res. Commun. 521 (2), 408–413. 10.1016/j.bbrc.2019.10.146 31668922

[B339] Wang Z. Liu S. Kakizaki M. Hirose Y. Ishikawa Y. Funato H. (2014a). Orexin/hypocretin activates mtor complex 1 (Mtorc1) *via* an Erk/Akt-Independent and calcium-stimulated lysosome V-Atpase pathway. J. Biol. Chem. 289 (46), 31950–31959. 10.1074/jbc.M114.600015 25278019 PMC4231673

[B340] Wang Z. H. Ni X. L. Li J. N. Xiao Z. Y. Wang C. Zhang L. N. (2014b). Changes in plasma Orexin-a levels in sevoflurane-remifentanil anesthesia in young and elderly patients undergoing elective lumbar surgery. Anesth. Analg. 118 (4), 818–822. 10.1213/ANE.0000000000000109 24651236

[B341] Wang C. Pan Y. Zhang R. Bai B. Chen J. Randeva H. S. (2014c). Heterodimerization of mouse orexin type 2 receptor variants and the effects on signal transduction. Biochim. Biophys. Acta 1843 (3), 652–663. 10.1016/j.bbamcr.2013.12.010 24368186

[B342] Wang C. Wang Q. Ji B. Pan Y. Xu C. Cheng B. (2018). The Orexin/Receptor System: molecular mechanism and therapeutic potential for neurological diseases. Front. Mol. Neurosci. 11, 220. 10.3389/fnmol.2018.00220 30002617 PMC6031739

[B343] Wang L. He T. Wan B. Wang X. Zhang L. (2019). Orexin a ameliorates Hbv X protein-induced cytotoxicity and inflammatory response in human hepatocytes. Artif. Cells Nanomed Biotechnol. 47 (1), 2003–2009. 10.1080/21691401.2019.1614014 31106596

[B344] Wang X. Guan R. Zhao X. Chen J. Zhu D. Shen L. (2021). Task1 and Task3 in Orexin Neuron of lateral hypothalamus contribute to respiratory chemoreflex by projecting to nucleus tractus solitarius. FASEB J. 35 (5), e21532. 10.1096/fj.202002189R 33817828

[B345] Wang H. Foong J. P. P. Harris N. L. Bornstein J. C. (2022). Enteric neuroimmune interactions coordinate intestinal responses in health and disease. Mucosal Immunol. 15 (1), 27–39. 10.1038/s41385-021-00443-1 34471248 PMC8732275

[B346] Wang L. Wang R. Song M. Lu W. Li N. Gao Y. (2023). Association between peripheral orexin a/B levels and depression with childhood trauma. J. Affect Disord. 340, 592–597. 10.1016/j.jad.2023.06.060 37385389

[B347] Wang Y. Wan X. Li Y. (2024a). Orexin B alleviates sepsis-associated lung injury through the attenuation of pulmonary endothelial barrier dysfunction by regulating the rho-associated coiled-coil containing protein kinase 2/Zonula Occludens-1 (Rock2/Zo-1) axis. Biotechnol. Appl. Biochem. 72, 883–896. 10.1002/bab.2703 39623599

[B348] Wang Y. Deng T. Zhao X. Shao L. Chen J. Fu C. (2024b). Control of breathing by orexinergic signaling in the nucleus tractus solitarii. Sci. Rep. 14 (1), 7473. 10.1038/s41598-024-58075-x 38553555 PMC10980752

[B349] Wang Y. Fu S. Mao J. Cui K. Jiang H. (2025a). Hypocretin: a promising target for the regulation of homeostasis. Front. Neurosci. 19, 1638270. 10.3389/fnins.2025.1638270 40927422 PMC12415059

[B350] Wang H. Kang R. Tang D. (2025b). Dopamine as an endogenous regulator of innate immunity in sepsis. Front. Immunol. 16, 1625368. 10.3389/fimmu.2025.1625368 40703506 PMC12283268

[B351] Ward R. J. Pediani J. D. Milligan G. (2011). Heteromultimerization of cannabinoid Cb(1) receptor and orexin Ox(1) receptor generates a unique complex in which both protomers are regulated by orexin A. J. Biol. Chem. 286 (43), 37414–37428. 10.1074/jbc.M111.287649 21908614 PMC3199489

[B352] Waters K. (2022). Review of the efficacy and safety of lemborexant, a dual receptor orexin antagonist (Dora), in the treatment of adults with insomnia disorder. Ann. Pharmacother. 56 (2), 213–221. 10.1177/10600280211008492 34078141

[B353] Weber C. Furuta M. Plump A. (2024). Fy2024 Q2 Earnings Announcement.

[B354] Weinhold S. L. Seeck-Hirschner M. Nowak A. Hallschmid M. Goder R. Baier P. C. (2014). The effect of intranasal Orexin-a (Hypocretin-1) on sleep, wakefulness and attention in Narcolepsy with cataplexy. Behav. Brain Res. 262, 8–13. 10.1016/j.bbr.2013.12.045 24406723

[B355] Wen J. Zhao Y. Shen Y. Guo L. (2015). Effect of orexin a on apoptosis in Bgc-823 gastric cancer cells *via* Ox1r through the akt signaling pathway. Mol. Med. Rep. 11 (5), 3439–3444. 10.3892/mmr.2015.3190 25586545

[B356] Wenzel J. Grabinski N. Knopp C. A. Dendorfer A. Ramanjaneya M. Randeva H. S. (2009). Hypocretin/Orexin increases the expression of steroidogenic enzymes in Human adrenocortical Nci H295r cells. Am. J. Physiol. Regul. Integr. Comp. Physiol. 297 (5), R1601–R1609. 10.1152/ajpregu.91034.2008 19793950

[B357] Williams J. T. Bolli M. H. Brotschi C. Sifferlen T. Steiner M. A. Treiber A. (2024). Discovery of Nivasorexant (Act-539313): the first selective Orexin-1 receptor antagonist (So1ra) investigated in clinical trials. J. Med. Chem. 67 (4), 2337–2348. 10.1021/acs.jmedchem.3c01894 38331429

[B358] Xie S. Chen M. Yan B. He X. Chen X. Li D. (2014). Identification of a role for the Pi3k/Akt/Mtor signaling pathway in innate immune cells. PLoS One 9 (4), e94496. 10.1371/journal.pone.0094496 24718556 PMC3981814

[B359] Xiong X. White R. E. Xu L. Yang L. Sun X. Zou B. (2013). Mitigation of Murine Focal cerebral ischemia by the Hypocretin/Orexin system is associated with reduced inflammation. Stroke 44 (3), 764–770. 10.1161/STROKEAHA.112.681700 23349191 PMC3638929

[B360] Xu D. Kong T. Shao Z. Liu M. Zhang R. Zhang S. (2021). Orexin-a alleviates astrocytic apoptosis and inflammation *via* inhibiting Ox1r-Mediated Nf-Kappab and mapk signaling pathways in cerebral ischemia/reperfusion Injury. Biochim. Biophys. Acta Mol. Basis Dis. 1867 (11), 166230. 10.1016/j.bbadis.2021.166230 34358627

[B361] Yadav A. Singh S. K. Sarkar D. (2023). Localization and expression of orexin B (Oxb) and its type 2 receptor (Ox2r) in mouse Testis during postnatal development. Peptides 164, 170979. 10.1016/j.peptides.2023.170979 36841281

[B362] Yang B. Ferguson A. V. (2003). Orexin-a depolarizes nucleus tractus solitarius neurons through effects on nonselective cationic and K+ conductances. J. Neurophysiol. 89 (4), 2167–2175. 10.1152/jn.01088.2002 12611968

[B363] Yang B. Samson W. K. Ferguson A. V. (2003). Excitatory effects of Orexin-a on nucleus tractus solitarius neurons are mediated by phospholipase C and protein kinase C. J. Neurosci. 23 (15), 6215–6222. 10.1523/JNEUROSCI.23-15-06215.2003 12867505 PMC6740536

[B364] Yang X. A. Song C. G. Yuan F. Zhao J. J. Jiang Y. L. Yang F. (2020). Prognostic roles of sleep electroencephalography pattern and circadian rhythm biomarkers in the recovery of consciousness in patients with coma: a prospective cohort study. Sleep. Med. 69, 204–212. 10.1016/j.sleep.2020.01.026 32143064

[B365] Yang M. Gan J. Liu S. Yang Y. Han J. Meng Q. (2024). Associations between plasma Orexin-a level and constipation in cognitive impairment. J. Alzheimers Dis. 97 (1), 409–419. 10.3233/JAD-230625 38143347

[B366] Yilmaz E. Celik O. Celik N. Celik E. Turkcuoglu I. Simsek Y. (2013a). Maternal and fetal serum Orexin-a levels in gestational diabetes mellitus. J. Obstet. Gynaecol. Res. 39 (1), 139–145. 10.1111/j.1447-0756.2012.01955.x 22889404

[B367] Yilmaz E. Celik O. Celik N. Simsek Y. Celik E. Yildirim E. (2013b). Serum Orexin-a (Oxa) level decreases in polycystic ovarian syndrome. Gynecol. Endocrinol. 29 (4), 388–390. 10.3109/09513590.2012.754874 23350701

[B368] Yilmaz M. Aksin S. Balsak D. Aboalhasan Y. Batmaz I. (2024). Comparison of orexigenic and anorexigenic neuropeptide levels in hyperemesis gravidarum patients with normal pregnant women: a prospective cohort study. Med. Baltim. 103 (42), e40069. 10.1097/MD.0000000000040069 39432622 PMC11495732

[B369] Yin J. Kang Y. McGrath A. P. Chapman K. Sjodt M. Kimura E. (2022). Molecular mechanism of the wake-promoting agent Tak-925. Nat. Commun. 13 (1), 2902. 10.1038/s41467-022-30601-3 35614071 PMC9133036

[B370] You W. R. Lin L. C. Lin W. C. Tsai M. C. (2022). Differences in Orexin-a level in the functional brain network of Hud patients undergoing harm reduction therapy. Med. Baltim. 101 (33), e30093. 10.1097/MD.0000000000030093 PMC9387983 35984180

[B371] Yuan L. B. Dong H. L. Zhang H. P. Zhao R. N. Gong G. Chen X. M. (2011). Neuroprotective effect of Orexin-a is mediated by an increase of hypoxia-inducible Factor-1 activity in rat. Anesthesiology 114 (2), 340–354. 10.1097/ALN.0b013e318206ff6f 21239965

[B372] Yun S. Wennerholm M. Shelton J. E. Bonaventure P. Letavic M. A. Shireman B. T. (2017). Selective inhibition of Orexin-2 receptors prevents stress-induced acth release in mice. Front. Behav. Neurosci. 11, 83. 10.3389/fnbeh.2017.00083 28533747 PMC5420581

[B373] Yurt S. Erman H. Korkmaz G. G. Kosar A. F. Uysal P. Gelisgen R. (2013). The role of feed regulating peptides on weight loss in patients with pulmonary tuberculosis. Clin. Biochem. 46 (1-2), 40–44. 10.1016/j.clinbiochem.2012.09.008 23000316

[B374] Zhang S. Blache D. Vercoe P. E. Adam C. L. Blackberry M. A. Findlay P. A. (2005). Expression of orexin receptors in the brain and peripheral tissues of the Male sheep. Regul. Pept. 124 (1-3), 81–87. 10.1016/j.regpep.2004.07.010 15544844

[B375] Zhang C. Abdukerim M. Abilailieti M. Tang L. Ling Y. Pan S. (2019). The protective effects of orexin a against high glucose-induced activation of Nlrp3 inflammasome in human vascular endothelial cells. Arch. Biochem. Biophys. 672, 108052. 10.1016/j.abb.2019.07.017 31351069

[B376] Zhang R. Li D. Mao H. Wei X. Xu M. Zhang S. (2022). Disruption of 5-Hydroxytryptamine 1a receptor and orexin receptor 1 heterodimer Formation affects novel G protein-dependent signaling pathways and has antidepressant effects *in vivo* . Transl. Psychiatry 12 (1), 122. 10.1038/s41398-022-01886-1 35338110 PMC8956632

[B377] Zhou F. Yan X. D. Wang C. He Y. X. Li Y. Y. Zhang J. (2020). Suvorexant ameliorates cognitive impairments and pathology in App/Ps1 transgenic mice. Neurobiol. Aging 91, 66–75. 10.1016/j.neurobiolaging.2020.02.020 32224066

[B378] Zhou X. Tang Y. Chen H. Huang W. Qiu H. (2026). Brain-region-mediated neuroimmune modulation in sepsis: research advances and therapeutic prospects. J. Inflamm. Res. 19, 570541. 10.2147/JIR.S570541 41709971 PMC12912014

[B379] Zhu L. Y. Summah H. Jiang H. N. Qu J. M. (2011). Plasma Orexin-a levels in copd patients with hypercapnic respiratory failure. Mediat. Inflamm. 2011, 754847. 10.1155/2011/754847 21765621 PMC3134321

[B380] Zhu Y. He J. Shi J. Wang Y. Ma L. Gou H. (2020). Significance of determining plasma orexin levels and analysis of related factors for the diagnosis of patients with Narcolepsy. Sleep. Med. 74, 141–144. 10.1016/j.sleep.2020.06.023 32858274

[B381] Zhu J. Zeng Z. Xiong M. Mo H. Jin M. Hu K. (2022). Associations between daytime and nighttime plasma orexin a levels and cognitive function in patients with obstructive sleep apnea. Sleep. Biol. Rhythms 20 (3), 421–429. 10.1007/s41105-022-00387-4 38469416 PMC10900028

[B382] Zhu M. Li X. Guo J. Zhang Z. Guo X. Li Z. (2024a). Orexin a protects against cerebral ischemia-reperfusion injury by enhancing reperfusion in ischemic cortex *via* Hif-1alpha-Et-1/Enos pathway. Brain Res. Bull. 218, 111105. 10.1016/j.brainresbull.2024.111105 39442584

[B383] Zhu Z. Chen G. He J. Xu Y. (2024b). The protective effects of orexin B in neuropathic pain by suppressing inflammatory response. Neuropeptides 108, 102458. 10.1016/j.npep.2024.102458 39255695

[B384] Zhu M. Guo X. Guo J. Zhang Z. Zhang K. Lei Y. (2025). The role of orexin a in the pathogenesis of ischaemic stroke at high altitude. Exp. Physiol. 111, 257–269. 10.1113/EP092314 40448667 PMC12756877

[B385] Zhu M. Zhang Z. Fu J. Guo J. Zhan H. Guo X. (2026). Orexin a ameliorates high altitude induced memory retrieval impairment *via* Bdnf/Trkb and Pi3k/Akt/Mtor signaling pathways. Behav. Brain Res. 496, 115866. 10.1016/j.bbr.2025.115866 41072850

[B386] Ziolkowska A. Spinazzi R. Albertin G. Nowak M. Malendowicz L. K. Tortorella C. (2005). Orexins stimulate glucocorticoid secretion from cultured rat and human adrenocortical cells, exclusively acting *via* the Ox1 receptor. J. Steroid Biochem. Mol. Biol. 96 (5), 423–429. 10.1016/j.jsbmb.2005.05.003 16157481

[B387] Ziolkowski M. Czarnecki D. Budzynski J. Rosinska Z. Zekanowska E. Goralczyk B. (2016). Orexin in patients with alcohol dependence treated for relapse prevention: a pilot Study. Alcohol Alcohol 51 (4), 416–421. 10.1093/alcalc/agv129 26597795

