# Supplementary material for: The orexin system as a pharmacological target in inflammation: peripheral mechanisms and safety implications
Source: Front Pharmacol. 2026 Aug 3;17:1831913. doi: 10.3389/fphar.2026.1831913 (PMC13478096; doi:10.3389/fphar.2026.1831913)
Supplement: Supplementary file 1 [file Supplementaryfile1.docx]

Supplementary Material

| "Orexin plasma" | | | | "Orexin serum" | | "Orexin blood" | |
| --- | --- | --- | --- | --- | --- | --- | --- |
| 1 | 10.1002/ajmg.a.37749 | 44 | 10.3233/JAD-220082 | 56 | 10.1002/brb3.2845 | 97 | 10.1093/alcalc/agv129 |
| 2 | 10.1002/eat.20857 | 45 | 10.3233/JAD-230625 | 57 | 10.1002/jcla.24170 | 98 | 10.1007/s11154-020-09559-4 |
| 3 | 10.1007/s40519-016-0271-y | 46 | 10.33549/physiolres.930860 | 58 | 10.1007/s00393-023-01462-y | 99 | 10.1007/s12032-015-0497-y |
| 4 | 10.1007/s40618-022-01841-3 | 47 | 10.3389/fendo.2018.00710 | 59 | 10.1007/s10068-022-01094-z | 100 | 10.1016/j.clinbiochem.2012.09.008 |
| 5 | 10.1007/s41105-022-00387-4 | 48 | 10.3389/fnagi.2022.1004002 | 60 | 10.1007/s10072-018-3692-8 | 101 | 10.1016/j.jflm.2019.06.009 |
| 6 | 10.1016/j.jad.2020.08.024 | 49 | 10.3389/fpsyt.2021.676336 | 61 | 10.1007/s10072-019-3708-z | 102 | 10.1016/j.jfma.2022.03.008 |
| 7 | 10.1016/j.jad.2023.06.060 | 50 | 10.3389/fpsyt.2022.900135 | 62 | 10.1007/s10792-019-01157-x | 103 | 10.1016/j.neulet.2013.03.017 |
| 8 | 10.1016/j.maturitas.2017.01.016 | 51 | 10.3390/brainsci10030129 | 63 | 10.1007/s11325-021-02514-w | 104 | 10.1016/j.psyneuen.2022.105679 |
| 9 | 10.1016/j.neulet.2020.135480 | 52 | 10.3892/etm.2014.1611 | 64 | 10.1016/j.dsx.2017.07.037 | 105 | 10.1016/j.psyneuen.2024.107119 |
| 10 | 10.1016/j.neulet.2022.136695 | 53 | 10.4183/aeb.2017.249 | 65 | 10.1016/j.eplepsyres.2022.106972 | 106 | 10.1016/j.soard.2016.05.023 |
| 11 | 10.1016/j.peptides.2016.12.008 | 54 | 10.5152/pcp.2021.20011 | 66 | 10.1016/j.jns.2014.08.041 | 107 | 10.1080/08039488.2022.2048887 |
| 12 | 10.1016/j.peptides.2019.170092 | 55 | PMID: 16008971 | 67 | 10.1016/j.molmet.2023.101713 | 108 | 10.1089/thy.2014.0515 |
| 13 | 10.1016/j.psyneuen.2014.12.012 |  | 10.1081/ias-120027225 | 68 | 10.1016/j.pediatrneurol.2023.04.017 | 109 | 10.1111/cob.12488 |
| 14 | 10.1016/j.regpep.2005.05.003 |  | 10.1159/000054724 | 69 | 10.1016/j.psychres.2016.02.059 | 110 | 10.1371/journal.pone.0084803 |
| 15 | 10.1016/j.rmed.2011.08.014 |  | 10.1159/000079643 | 70 | 10.1016/j.psychres.2018.04.010 | 111 | 10.1542/peds.110.6.e74 |
| 16 | 10.1016/j.sleep.2020.06.023 |  | 10.1186/s40981-022-00498-4 | 71 | 10.1016/j.psyneuen.2024.107169 | 112 | 10.1556/2006.8.2019.65 |
| 17 | 10.1016/s0196-9781(00)00369-7 |  | 10.1378/chest.125.5.1963 | 72 | 10.1016/j.sleep.2020.01.026 | 113 | 10.33549/physiolres.933467 |
| 18 | 10.1016/s0196-9781(03)00055-x |  | PMID: 16135994 | 73 | 10.1016/j.sleep.2022.10.019 |  | 10.1177/02698811231225610 |
| 19 | 10.1016/s0304-3940(01)02476-4 |  | PMID: 18058738 | 74 | 10.1016/s1734-1140(12)70940-4 |  | 10.1212/wnl.58.3.498 |
| 20 | 10.1038/s41598-019-44450-6 |  | PMID: 22805624 | 75 | 10.1037/pha0000395 |  |  |
| 21 | 10.1038/sj.ijo.0801868 |  | PMID: 23339004 | 76 | 10.1080/0092623X.2019.1566947 |  |  |
| 22 | 10.1080/08039488.2020.1755995 |  | PMID: 23748284 | 77 | 10.1080/08164622.2024.2374875 |  |  |
| 23 | 10.1093/braincomms/fcab050 |  | 10.1210/jc.2004-1408 | 78 | 10.1080/13651501.2019.1711425 |  |  |
| 24 | 10.1093/ijnp/pyy075 |  |  | 79 | 10.1080/17470919.2016.1169216 |  |  |
| 25 | 10.1093/schbul/sbab042 |  |  | 80 | 10.1097/MD.0000000000006876 |  |  |
| 26 | 10.1097/YIC.0b013e3283377850 |  |  | 81 | 10.1097/MD.0000000000030093 |  |  |
| 27 | 10.1111/j.1471-0528.2009.02474.x |  |  | 82 | 10.1097/MD.0000000000040069 |  |  |
| 28 | 10.1111/psyg.12444 |  |  | 83 | 10.1111/ajad.13064 |  |  |
| 29 | 10.1155/2011/754847 |  |  | 84 | 10.1111/j.1447-0756.2012.01955.x |  |  |
| 30 | 10.1159/000080453 |  |  | 85 | 10.1111/jog.14758 |  |  |
| 31 | 10.1159/000081757 |  |  | 86 | 10.1677/jme.1.01818 |  |  |
| 32 | 10.1177/0333102410392605 |  |  | 87 | 10.18071/isz.75.0223 |  |  |
| 33 | 10.1177/147323000903700207 |  |  | 88 | 10.18544/PEDM-21.03.0031 |  |  |
| 34 | 10.1177/1533317513494453 |  |  | 89 | 10.2500/aap.2024.45.240023 |  |  |
| 35 | 10.1186/1471-230X-9-92 |  |  | 90 | 10.30773/pi.2023.0309 |  |  |
| 36 | 10.1212/wnl.56.12.1749 |  |  | 91 | 10.3109/09513590.2012.754874 |  |  |
| 37 | 10.1213/ANE.0000000000000109 |  |  | 92 | 10.3390/ijms25116243 |  |  |
| 38 | 10.1297/cpe.13.47 |  |  | 93 | 10.3892/ijmm.2010.590 |  |  |
| 39 | 10.1378/chest.124.4.1381 |  |  | 94 | 10.4274/jcrpe.1930 |  |  |
| 40 | 10.1378/chest.127.3.731 |  |  | 95 | PMCID: PMC10331673 |  |  |
| 41 | 10.1515/jbcpp-2015-0133 |  |  | 96 | PMID: 19099733 |  |  |
| 42 | 10.2147/DMSO.S257687 |  |  |  | 10.1016/j.gastrohep.2019.10.006 |  |  |
| 43 | 10.2147/NDT.S209023 |  |  |  | 10.13703/j.0255-2930.2018.10.003 |  |  |

**Supplementary Table 1.** Articles identified through a systematic PubMed search on circulating orexin concentrations in humans using the keyword combinations “orexin plasma,” “orexin serum,” and “orexin blood.” Articles highlighted in red were excluded from the review (see main text).

**Supplementary Table 2:** The distribution of individual studies across reported OxA concentration ranges.

Sex: B – Both Sexes; F – Female; M – Male.

Manufacturer: 1 - Peninsula Laboratories; 2 - USCN Life Science; 3 - Phoenix Pharmaceuticals; 4 – CUSABIO; 5 – RayBiotech; 6 – MyBioscource; 7 - R&D Systems; 8 - Elabscience Biotechnology; 9 - Abbexa; 10 - YH Biosearch Laboratory; 11 – Millipore; 12- Shanghai Sunred Biological Technology; 13 - YL Biotech; 14 - ELK Biotechnology; 15 - BT-laboratory; 16 - Wuhan Cloud-Clone Corp; 17 - Zell Bio GmbH; 18 – Bostonchem; 19 - Jiangsu Meimian Industry; 20 - Huinuo Biological Technology; IHD – In-House Developed Methodology; HNMP – Human Neuropeptide Magnetic Bead Panel.

Method: E – ELISA; MX – Multiplex; R – RIA. Time of collection: I - Immediate Processing of Samples. Sample type: P – Plasma; S – Serum. NS – not specified.

| **Range ng/mL** | **Sex** | **Age**  **(years)** | **Manufacturer**  **(Catalogue Number)** | **Method** | **Preanalytical Handling** | | | | | **Year** | **DOI** |
| --- | --- | --- | --- | --- | --- | --- | --- | --- | --- | --- | --- |
|  |  |  |  |  | Extraction | Acidification | Protease Inhibitor | Time of collection | Plasma/Serum |  |  |
| 1000-10000 | F | 40-65 | 1 | R | NS | NS | NS | NS | P | 2010 | 10.1111/j.1471-0528.2009.02474.x |
| 10-100 | M | 40-65 | 2 | E | NS | NS | NS | NS | S | 2016 | 10.1093/alcalc/agv129 |
|  | M | 18-40 | 3 | E | NS | NS | NS | NS | S | 2022 | 10.1007/s10068-022-01094-z |
|  | B | 1-10 | 4 (CSB-E08859h) | E | NS | NS | NS | NS | S | 2024 | 10.1016/j.psyneuen.2024.107169 |
|  | B | 40-65 | 3 | E | NS | NS | YES | NS | P | 2009 | 10.1177/147323000903700207 |
|  | F | 18-40 | 1 | E | NS | NS | NS | NS | S | 2013 | 10.3109/09513590.2012.754874 |
|  | M | >65 | 3 | E | NS | NS | NS | NS | P | 2022 | 10.3389/fnagi.2022.1004002 |
|  | M | >65 | 1 (S-1374) | E | NS | NS | NS | NS | S | 2011 | 10.3892/ijmm.2010.590 |
| 1-10 | B | 40-65 | 3 | E | NS | NS | NS | I | P | 2011 | 10.1016/j.rmed.2011.08.014 |
|  | B | 1-10 | 8 | E | NS | NS | NS | NS | S | 2019 | 10.1007/s10072-018-3692-8 |
|  | B | 40-65 | 10 | E | NS | NS | NS | NS | S | 2019 | 10.1007/s10072-019-3708-z |
|  | B | 40-65 | 10 | E | NS | NS | NS | NS | S | 2020 | 10.1007/s10792-019-01157-x |
|  | B | 40-65 | 4 | E | NS | NS | NS | NS | S | 2015 | 10.1007/s12032-015-0497-y |
|  | B | 40-65 | 1 | E | NS | NS | NS | NS | S | 2013 | 10.1016/j.clinbio1-10yem.2012.09.008 |
|  | B | 18-40 | 9 (abNS252876) | E | NS | NS | NS | NS | S | 2022 | 10.1016/j.eplepsyres.2022.106972 |
|  | B | 40-65 | 7 (NBP2-80230) | E | NS | NS | NS | NS | P | 2023 | 10.1016/j.jad.2023.06.060 |
|  | B | 40-65 | 3 (EKE-003-30) | E | NS | NS | NS | NS | S | 2022 | 10.1016/j.jfma.2022.03.008 |
|  | F | 40-65 | 1 (S-1374) | E | NS | NS | NS | I | P | 2017 | 10.1016/j.maturitas.2017.01.016 |
|  | M | 18-40 | 3 | E | NS | NS | NS | NS | S | 2013 | 10.1016/j.neulet.2013.03.017 |
|  | B | >65 | 3 (EK-003-30) | E | NS | NS | NS | I | P | 2021 | 10.1016/j.neulet.2020.135480 |
|  | B | >65 | 7 | E | NS | NS | NS | NS | P | 2022 | 10.1016/j.neulet.2022.136695 |
|  | B | 1-10 | 8 | E | NS | NS | NS | NS | S | 2023 | 10.1016/j.pediatrneurol.2023.04.017 |
|  | M | 40-65 | 3 | E | NS | NS | NS | NS | S | 2016 | 10.1016/j.psy1-10yres.2016.02.059 |
|  | F | 18-40 | 3 | E | NS | NS | NS | NS | P | 2019 | 10.1038/s41598-019-44450-6 |
|  | B | 40-65 | 4 (CSB-E08859h) | E | NS | NS | NS | NS | P | 2020 | 10.1080/08039488.2020.1755995 |
|  | B | 40-65 | 3 | E | NS | NS | NS | NS | S | 2015 | 10.1089/thy.2014.0515 |
|  | B | 40-65 | 3 (EKE-003-30) | E | NS | NS | NS | I | S | 2019 | 10.1093/ijnp/pyy075 |
|  | B | 40-65 | 6 (MBS263266) | E | NS | NS | NS | NS | P | 2019 | 10.1093/ijnp/pyy075 |
|  | F | 40-65 | NS | E | NS | NS | NS | NS | S | 2012 | 10.1111/j.1447-0756.2012.01955.x |
|  | B | <1 | 3 | E | NS | NS | YES | I | P | 2009 | 10.1186/1471-230X-9-92 |
|  | B | 40-65 | 5 (EIA‑ORA‑1) | E | NS | NS | NS | NS | S | 2014 | 10.1371/journal.pone.0084803 |
|  | B | 1-10 | 3 | E | NS | NS | NS | NS | S | 2016 | 10.18544/PEDM-21.03.0031 |
|  | B | >65 | 3 | E | NS | NS | YES | NS | P | 2014 | 10.3892/etm.2014.1611 |
|  | B | 1-10 | 2 (CEA607Hu) | E | NS | NS | NS | NS | S | 2015 | 10.4274/jcrpe.1930 |
|  | M | 40-65 | 3 | E | YES | NS | YES | NS | P | 2020 | 10.2147/DMSO.S257687 |
|  | M | 18-40 | 3 | E | YES | NS | YES | I | P | 2015 | 10.1515/jbcpp-2015-0133 |
|  | B | NS | NS | NS | NS | NS | NS | NS | P | 2022 | 10.3233/JAD-220082 |
|  | NS | NS | NS | NS | NS | NS | NS | NS | P | 2023 | 10.3233/JAD-230625 |
| 0.1-1 | B | 40-65 | 4 (CSB‐E08859h) | E | NS | NS | NS | NS | S | 2022 | 10.1002/jcla.24170 |
|  | B | 40-65 | 2 | E | NS | NS | NS | NS | S | 2024 | 10.1007/s00393-023-01462-y |
|  | B | 40-65 | 8 | E | NS | NS | NS | NS | S | 2022 | 10.1007/s11325-021-02514-w |
|  | M | 40-65 | 6 | E | NS | NS | NS | NS | S | 2018 | 10.1016/j.psy1-10yres.2018.04.010 |
|  | F | 18-40 | 4 | E | NS | NS | NS | NS | S | 2023 | 10.1016/j.sleep.2022.10.019 |
|  | B | NS | NS | E | NS | NS | NS | NS | S | 2021 | 10.1037/pha0000395 |
|  | F | 18-40 | 17 | E | NS | NS | NS | NS | S | 2019 | 10.1080/0092623X.2019.1566947 |
|  | B | 40-65 | 13 (YLA1769HU) | E | NS | NS | NS | NS | S | 2022 | 10.1080/08039488.2022.2048887 |
|  | B | 10-18 | 6 | E | NS | NS | NS | NS | S | 2020 | 10.1080/13651501.2019.1711425 |
|  | B | 40-65 | 6 | E | NS | NS | NS | NS | S | 2022 | 10.1097/MD.0000000000030093 |
|  | B | 40-65 | 18 (BLS-1465Hu) | E | NS | NS | NS | NS | S | 2024 | 10.1097/MD.0000000000040069 |
|  | F | 40-65 | 17 | E | NS | NS | NS | NS | S | 2021 | 10.1111/jog.14758 |
|  | B | 40-65 | 1 | E | NS | NS | NS | NS | P | 2013 | 10.1177/1533317513494453 |
|  | B | 40-65 | 15 (E1296Hu) | E | NS | NS | NS | NS | S | 2022 | 10.18071/isz.75.0223 |
|  | B | 40-65 | 12 (201-12-1297) | E | NS | NS | NS | NS | S | 2024 | 10.30773/pi.2023.0309 |
|  | M | 40-65 | 16 (CEA607Hu) | E | NS | NS | NS | NS | P | 2020 | 10.3389/fpsyt.2022.900135 |
|  | B | 40-65 | 14 | E | NS | NS | NS | NS | S | 2024 | 10.3390/ijms25116243 |
|  | NS | NS | NS | E | NS | NS | NS | NS | S | 2008 | PMID: 19099733 |
|  | B | 1-10 | 3 (EK-003-30) | E | YES | NS | NS | NS | S | 2012 | 10.1016/s1734-1140(12)70940-4 |
|  | B | 1-10 | 1 | E | YES | YES | YES | I | P | 2004 | 10.1159/000080453 |
|  | B | 1-10 | 3 | E | YES | NS | YES | I | P | 2020 | 10.3390/brainsci10030129 |
|  | B | 1-10 | 11 (HNMP) | MX | NS | NS | NS | I | P | 2016 | 10.1002/ajmg.a.37749 |
|  | B | 40-65 | 11 | MX | NS | NS | NS | NS | S | 2021 | 10.1111/ajad.13064 |
|  | B | 10-18 | 11 (HNCSMAG-35K) | MX | NS | NS | NS | NS | S | 2020 | 10.1556/2006.8.2019.65 |
|  | B | 18-40 | 11 (HNMP) | MX | NS | NS | NS | NS | P | 2017 | 10.4183/aeb.2017.249 |
|  | B | 40-65 | 3 (RK-003–30) | R | YES | YES | NS | I | P | 2021 | 10.1093/braincomms/fcab050 |
|  | B | 40-65 | 3 | R | YES | NS | YES | I | P | 2001 | 10.1212/wnl.56.12.1749 |
|  | B | 40-65 | 1 | NS | NS | NS | NS | NS | S | 2014 | 10.1016/j.jns.2014.08.041 |
|  | F | 18-40 | NS | NS | NS | NS | NS | NS | S | 2017 | 10.1097/MD.0000000000006876 |
|  | B | 40-65 | NS | NS | NS | NS | NS | NS | S | 2024 | 10.2500/aap.2024.45.240023 |
| 0.01-0.1 | M | 18-40 | 3 | E | NS | NS | YES | NS | P | 2010 | 10.1097/YIC.0b013e3283377850 |
|  | B | >65 | 3 | E | YES | NS | NS | NS | S | 2023 | 10.1016/j.molmet.2023.101713 |
|  | B | >65 | 20 | E | NS | NS | NS | NS | S | 2022 | 10.1002/brb3.2845 |
|  | F | 40-65 | 9 | E | NS | NS | YES | I | S | 2020 | 10.1007/s11154-020-09559-4 |
|  | B | 40-65 | 4 | E | NS | NS | NS | I | P | 2017 | 10.1007/s40519-016-0271-y |
|  | F | 1-10 | 16 (CEA607Hu) | E | NS | NS | NS | NS | P | 2022 | 10.1007/s40618-022-01841-3 |
|  | B | 40-65 | 19 | E | NS | NS | NS | NS | P | 2022 | 10.1007/s41105-022-00387-4 |
|  | F | 18-40 | 3 (EK-003-30) | E | NS | NS | NS | NS | S | 2017 | 10.1016/j.dsx.2017.07.037 |
|  | B | 18-40 | NS | E | NS | NS | NS | NS | P | 2020 | 10.1016/j.sleep.2020.06.023 |
|  | B | 40-65 | 3 (FKE-003-30) | E | NS | NS | NS | NS | P | 2019 | 10.2147/NDT.S209023 |
|  | F | 40-65 | 3 (EKE-003-30) | E | NS | NS | NS | NS | S | 2018 | 10.33549/physiolres.933467 |
|  | B | 18-40 | 3 (EK-003-30) | E | YES | YES | YES | NS | P | 2021 | 10.3389/fpsyt.2021.676336 |
|  | B | 18-40 | 3 | E | YES | YES | NS | NS | P | 2020 | 10.1016/j.jad.2020.08.024 |
|  | B | 40-65 | 3 | E | YES | YES | YES | NS | P | 2017 | 10.1016/j.peptides.2016.12.008 |
|  | B | 40-65 | 3 | E | YES | NS | YES | I | P | 2005 | 10.1016/j.regpep.2005.05.003 |
|  | M | 40-65 | 1 | R | NS | NS | NS | NS | P | 2003 | 10.1378/1-10yest.124.4.1381 |
|  | B | 10-18 | 3 | R | NS | NS | NS | NS | S | 2022 | 10.1016/j.psyneuen.2022.105679 |
|  | B | 40-65 | 3 (RK-003-30) | R | NS | NS | NS | NS | S | 2020 | 10.1016/j.sleep.2020.01.026 |
|  | F | 40-65 | 3 | R | NS | NS | NS | NS | S | 2017 | 10.1080/17470919.2016.1169216 |
|  | B | 1-10 | 1 | R | NS | NS | NS | NS | P | 2004 | 10.1297/cpe.13.47 |
|  | F | NS | 3 | R | NS | NS | NS | NS | S | 2006 | 10.1677/jme.1.01818 |
|  | F | 10-18 | 3 | R | YES | YES | YES | I | P | 2010 | 10.1002/eat.20857 |
|  | B | >65 | 1 | R | YES | YES | YES | NS | P | 2019 | 10.1016/j.peptides.2019.170092 |
|  | B | 40-65 | 3 | R | YES | YES | YES | NS | P | 2015 | 10.1016/j.psyneuen.2014.12.012 |
|  | M | 40-65 | IHD | R | YES | YES | NS | NS | P | 2003 | 10.1016/s0196-9781(03)00055-x |
|  | B | 40-65 | 1 (RIK9600) | R | YES | NS | NS | I | P | 2002 | 10.1016/s0304-3940(01)02476-4 |
|  | B | NS | 3 | R | YES | YES | NS | I | P | 2002 | 10.1038/sj.ijo.0801868 |
|  | B | >65 | 3 | R | YES | YES | NS | NS | P | 2021 | 10.1093/s1-10ybul/sbab042 |
|  | B | >65 | 1 | R | YES | YES | YES | NS | P | 2019 | 10.1111/psyg.12444 |
|  | B | >65 | 3 | R | YES | NS | YES | NS | P | 2011 | 10.1155/2011/754847 |
|  | B | 40-65 | 3 | R | YES | NS | NS | I | P | 2004 | 10.1159/000081757 |
|  | B | 40-65 | 1 | R | YES | NS | NS | I | P | 2011 | 10.1177/0333102410392605 |
|  | B | >65 | 1 | R | YES | NS | NS | NS | P | 2014 | 10.1213/ANE.0000000000000109 |
|  | B | 40-65 | IHD | R | YES | YES | NS | NS | P | 2005 | 10.1378/1-10yest.127.3.731 |
|  | B | 10-18 | 3 | R | YES | NS | YES | NS | S | 2002 | 10.1542/peds.110.6.e74 |
|  | F | 1-10 | 3 | R | YES | YES | YES | I | P | 2007 | 10.33549/physiolres.930860 |
|  | M | 18-40 | 1 | R | YES | YES | NS | NS | P | 2021 | 10.5152/pcp.2021.20011 |
|  | B | 10-18 | NS | NS | NS | NS | NS | NS | S | 2017 | 10.1016/j.soard.2016.05.023 |
|  | B | 1-10 | NS | NS | NS | NS | NS | NS | S | 2022 | 10.1111/cob.12488 |
| 0.001-0.01 | B | >65 | 2 | E | NS | NS | NS | NS | S | 2024 | 10.1080/08164622.2024.2374875 |
|  | B | 40-65 | 8 (E-EL-H1015c) | E | NS | NS | NS | NS | S | 2023 | PMCID: PMC10331673 |
|  | M | 18-40 | IHD | R | YES | YES | NS | I | P | 2018 | 10.3389/fendo.2018.00710 |
|  | B | 40-65 | 3 | R | YES | YES | YES | I | P | 2001 | 10.1016/s0196-9781(00)00369-7 |
|  | B | 40-65 | 3 | R | YES | YES | YES | NS | P | 2005 | PMID: 16008971 |
| 0.0001-0.001 | B | 40-65 | 11 (HNPMAG-35K) | MX | NS | NS | NS | NS | S | 2024 | 10.1016/j.psyneuen.2024.107119 |

**Supplementary Calculations**

1. **Ehrström et al. 2005a**

Estimated steady-state concentration ~200pmol/L

Estimated time of reaching steady state ~100 minutes

*Original reported figure (modified): Mean ± sem plasma OXA concentrations after a solid meal during saline and iv OXA infusion (10 pmol/kg·min) in six healthy men. The infusion was started at −10 min and the meal consumed at 0 min.*

Based on the reaching of steady-state after approximately 100 minutes in the reported figure, the elimination half-life (t₁/₂) of OxA can be estimated at ~20 minutes, applying the principle of five half-lives to steady state.

Using the relationship between infusion rate (Q) and steady-state concentration (Css) systemic clearance (CL) can be approximated – Css (200pmol/L) = Q (10 pmol/kg·min)/CL. CL = Q/Css. CL = ~50 mL/min/kg.

Using clearance and t₁/₂, the apparent volume of distribution (Vd) can be calculated as (t_1/2_*CL)/0.693 = ~1.5 L/kg.

2. **Ehrström et al. 2004**

~175pmol/L

~350pmol/L

*Original reported figure (modified): Mean±S.D. plasma concentration–time profile of orexin A as calculated according to a third-degree polynomial. An IV infusion of orexin A 100 pmol kg−1min−1 was given for time periods of 0, 10, 20, 30 min and for 10, 20, 30 min after ceasing a 30-min infusion (n=4 for each time period).*

Ke = 0.693/t_1/2_ (20 ~30min) = 0.035 ~ 0.023

Vd = Dose/Concentration, adjusted for infusion duration and elimination using an infusion correction factor  $(*\frac{{1-e}^{-ke*infusion duration}}{ke*infusion duration})$

Vd = (100 pmol/kg/min*10~30min)/150~350pmol/L $*\frac{{1-e}^{-0.023\sim0.035*10\sim30min}}{0.023\sim0.035*10\sim30min}$ = 4.2~6.4 L/kg

Clearance = (Vd*0.693)/t₁/₂

Clearance = ((4.2~6.4 L/kg)*0.693)/(20 ~30min) = 100~200 mL/min/kg
